# Amides, Isoquinoline Alkaloids and Dipeptides from the Aerial Parts of *Piper mullesua*

**DOI:** 10.1007/s13659-018-0180-z

**Published:** 2018-08-02

**Authors:** Meng-Yuan Xia, Jun Yang, Pan-Hua Zhang, Xiao-Nian Li, Ji-Feng Luo, Chun-Lin Long, Yue-Hu Wang

**Affiliations:** 1 Key Laboratory of Economic Plants and Biotechnology and the Yunnan Key Laboratory for Wild Plant Resources, Kunming Institute of Botany, Chinese Academy of Sciences, Kunming, 650201 People’s Republic of China; 2 Southeast Asia Biodiversity Research Institute, Chinese Academy of Sciences, Yezin, Nay Pyi Taw, 05282 Myanmar; 3 State Key Laboratory of Phytochemistry and Plant Resources in West China, Kunming Institute of Botany, Chinese Academy of Sciences, Kunming, 650201 People’s Republic of China; 4 College of Life and Environmental Sciences, Minzu University of China, Beijing, 100081 People’s Republic of China; 5 Key Laboratory of Ethnomedicine (Minzu University of China), Ministry of Education, Beijing, 100081 People’s Republic of China

**Keywords:** *Piper mullesua*, Piperaceae, Antiplatelet, Amides, Isoquinoline alkaloids

## Abstract

**Abstract:**

One undescribed amide, pipermullesine A, two undescribed isoquinoline alkaloids, pipermullesines B and C, and six undescribed dipeptides, pipermullamides A–F, along with 28 known compounds, were isolated from the aerial parts of *Piper mullesua*. The structures of the undescribed compounds were elucidated based on the analysis of 1D and 2D NMR and MS data. Furthermore, the structures of pipermullesines A–C were confirmed by single crystal X-ray diffraction analysis. All isolates were evaluated for inhibitory activity against platelet aggregation induced by thrombin (IIa) or platelet-activating factor (PAF). (-)-Mangochinine, pellitorine, and (2*E*,4*E*)-*N*-isobutyl-2,4-dodecadienamide showed weak inhibitory activity against rabbit platelet aggregation induced by PAF, with IC_50_ values of 470.3 µg/mL, 614.9 µg/mL, and 579.7 µg/mL, respectively.

**Graphical Abstract:**

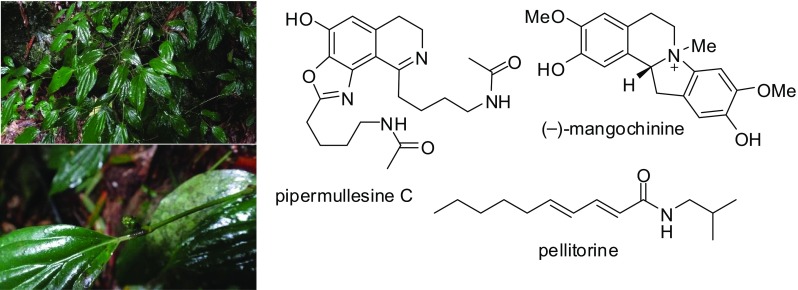

**Electronic supplementary material:**

The online version of this article (10.1007/s13659-018-0180-z) contains supplementary material, which is available to authorized users.

## Introduction

Traditional Chinese medicines with the functions of promoting blood circulation (“Huoxue” in Chinese) and/or removing blood stasis (“Huayu” in Chinese) are claimed to be useful in antiplatelet therapies and the treatment of thrombotic diseases [1]. For example, antiplatelet compounds have been found in a Huoxue herb *Selaginella moellendorffii* Hieron. (Selaginellaceae) [2, 3].

The genus *Piper* (Piperaceae) is a medicinally important group of plants consisting of approximately 2000 species worldwide. There are approximately 60 species distributed in the tropical areas of the People’s Republic of China, of which approximately 30 species have been used as traditional Chinese medicines [4]. Some *Piper* species are used for promoting blood circulation, while *Piper mullesua* Buch.-Ham. ex D. Don and *P. yunnanense* Tseng are used for removing blood stasis [5]. As a folk medicine in China with the Chinese name of Duan-Jv (短蒟), the whole plants of *P. mullesua* are also used to treat bleeding, bone fractures, injuries from falls, rheumatoid arthritis, rheumatic arthralgia, acroanesthesia, asthma, colds, stomach aches, abdominal pain, toothaches, swelling and pain of furuncles, dysmenorrhea, menoxenia, empyrosis, and snake and insect bites [5, 6].

Alcoholic extracts of *P. mullesua* showed the activity against rabbit platelet aggregation induced by 7.2 nM of the platelet-activating factor (PAF) with an IC_50_ value of 64.43 μg/mL [7]. Amides including retrofractamide A, chingchengenamide A [6], *N*-isobutyl-16-phenylhexadeca-2*E*,4*E*-dienamide, and *N*-isobutyldeca-2*E*,4*E*-dienamide [8], lignans including (-)-nectandrin A, nectandrin B, galgravin [6], asarinin, fargesin, and sesamin with antifeedant activity [8, 9], a phenylpropanoid myristicin with insecticidal activity [9, 10], and several arylalkenyl carboxylic acid esters [10, 11] have been isolated from the plants. However, the active constituents of *P. mullesua* responsible for the antiplatelet aggregation remain unclear. In continuing efforts to search for antiplatelet compounds from *Piper* plants [12, 13], we herein present the results of the analysis of compounds from the aerial parts of *P. mullesua* and the bioactivity of these compounds.

## Results and Discussion

### Structure Elucidation

**Fig. 1:**
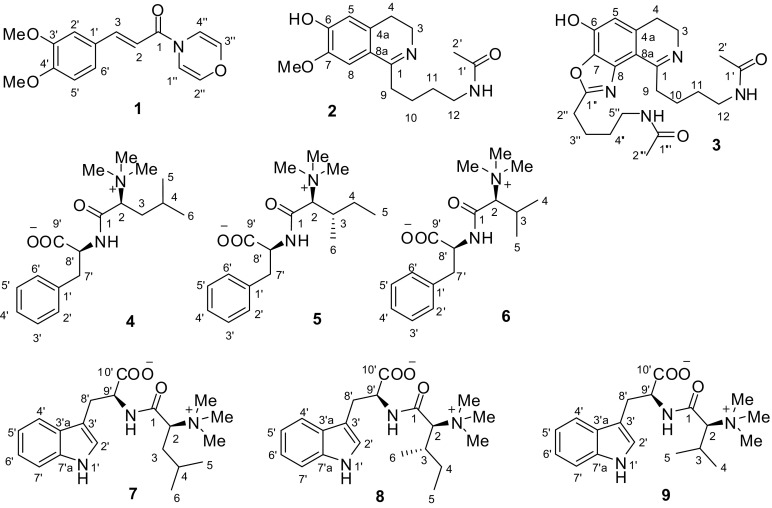
Structures of undescribed compounds (**1**–**9**) from *Piper mullesua*

Nine undescribed compounds (**1**–**9**, Fig. 1) and 28 known ones (**10**–**37**) were isolated from the methanolic extracts of *P. mullesua* by silica gel, D101 resin and Sephadex LH-20 column chromatography and semipreparative HPLC.

Pipermullesine A (**1**) had the molecular formula C_15_H_15_NO_4_ based on ^13^C NMR (Table 1) and HREIMS data. Its IR spectrum showed absorption peaks for a tertiary amide at 1643 cm^−1^ and a phenyl ring at 1595, 1513, and 1461 cm^−1^. The ^1^H NMR data (Table 1) indicated a 1,2,4-trisubstituted phenyl ring [*δ*_H_ 7.12 (1H, dd, *J *= 8.3, 1.8 Hz), 7.01 (1H, d, *J *= 1.8 Hz), and 6.86 (1H, d, *J *= 8.3 Hz)], an *E* double bond [*δ*_H_ 7.67 (1H, d, *J *= 15.3, 1.8 Hz) and 6.49 (1H, d, *J *= 15.3 Hz)], a 1,4-oxazine ring [*δ*_H_ 6.61 (1H, dd, *J *= 5.1, 1.9 Hz), 6.10 (1H, dd *J *= 5.1, 1.9 Hz), 5.84 (1H, d, *J *= 5.1 Hz), and 5.70 (1H, d, *J *= 5.1 Hz)] [14], and two methoxy groups [*δ*_H_ 3.92 (3H, s) and 3.91 (3H, s)]. The above NMR characteristic signals implied that compound **1** might be a cinnamamide derivative.

**Table 1:** ^1^H (500 MHz) and ^13^C NMR (125 MHz) NMR Data of **1** in CDCl_3_

No.	*δ*_H_ (*J* in Hz)	*δ*_C_, type
1		158.3, C
2	6.49, d (15.3)	112.0, CH
3	7.67, d (15.3)	144.4, CH
1′		128.0, C
2′	7.01, d (1.8)	110.0, CH
3′		149.3, C
4′		151.1, C
5′	6.86, d (8.3)	111.2, CH
6′	7.12, dd (8.3, 1.8)	122.4, CH
1″	6.61, dd (5.1, 1.9)	108.7, CH
2″	5.84, d (5.1)	132.7, CH
3″	5.70, d (5.1)	130.5, CH
4″	6.10, dd (5.1, 1.9)	109.4, CH
3′-OMe	3.92, s	56.1, CH_3_
4′-OMe	3.91, s	56.1, CH_3_

**Fig. 2:**
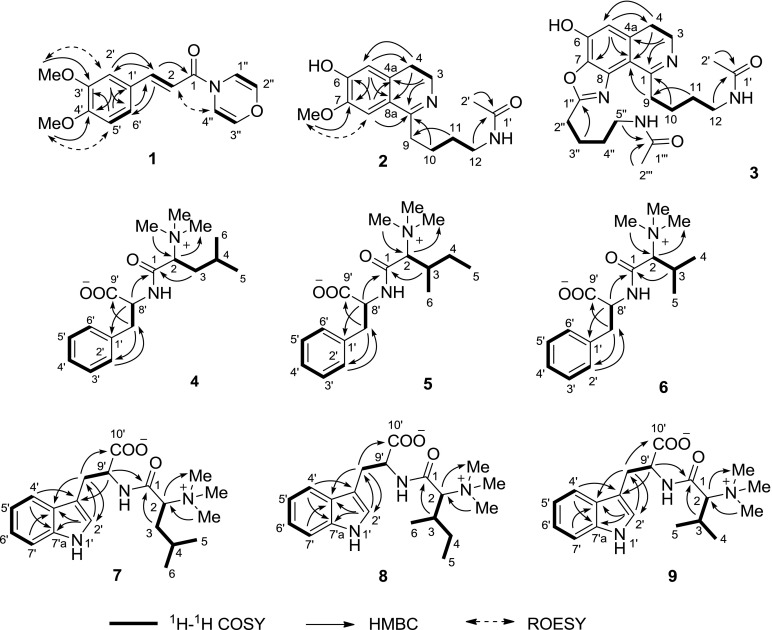
Key 2D NMR correlations of compounds **1**–**9**

**Fig. 3:**
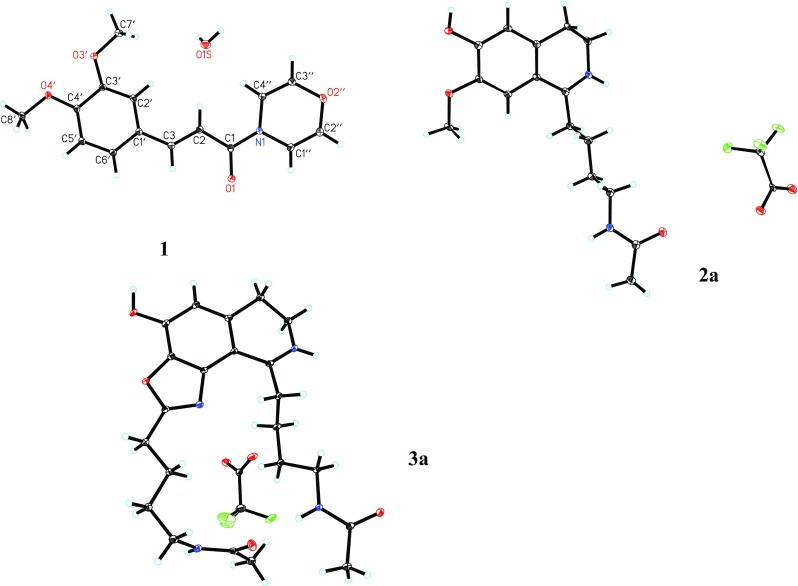
X-ray crystallographic structures of **1**, **2a**, and **3a**

According to the ^1^H–^1^H COSY and HMBC correlations of compound **1** (Fig. 2), (*E*)-3,4-dimethoxycinnamoyl and 1,4-oxazine groups were confirmed. Although the correlations from H-1″ and H-4″ to C-1 were not observed in the HMBC spectrum, the structure of **1** was finally determined as (*E*)-3-(3,4-dimethoxyphenyl)-1-(4*H*-1,4-oxazin-4-yl) prop-2-en-1-one by a single-crystal X-ray diffraction analysis (Fig. 3).

The molecular formula of pipermullesine B (**2**), C_16_H_22_N_2_O_3_, was determined by ^13^C NMR data (Table 2) and an HREIMS ion at *m/z* 290.1620 [M]^+^ (calcd for C_16_H_22_N_2_O_3_, 290.1630) and required 7 indices of hydrogen deficiency. The ^1^H NMR data (Table 2) indicated a tetrasubstituted phenyl ring [*δ*_H_ 7.15 (1H, s) and 6.52 (1H, s)], one methoxy group [*δ*_H_ 3.85 (3H, s)], and one acetyl group [*δ*_H_ 1.91 (3H, s)]. The ^13^C NMR data (Table 2) exhibited 15 signals. However, according to its HREIMS data, compound **2** should have 16 carbon atoms. The disappeared signal for C-9 (*δ*_C_ 33.0) was detected by the HMBC correlation (Fig. 2) from H_2_-11 to C-9.

**Table 2:** ^1^H and ^13^C NMR Data of **2** and **2a** in CD_3_OD

No.	**2**		**2a**	
	*δ*_H_ (*J* in Hz)^a^	*δ*_C_, type^b^	*δ*_H_ (*J* in Hz)^c^	*δ*_C_, type^d^
1		174.4, C		179.1, C
3	3.63, t (7.7)	42.4, CH_2_	3.77, t (7.9)	42.3, CH_2_
4	2.87, t (7.7)	27.1, CH_2_	3.01, t (7.9)	26.0, CH_2_
4a		137.6, C		136.4, C
5	6.52, s	118.2, CH	6.85, s	116.7, CH
6		167.6, C		158.3, C
7		151.1, C		149.2, C
8	7.15, s	112.2, CH	7.42, s	114.1, CH
8a		112.3, C		116.9, C
9	Disappeared	33.0^e^	Disappeared	33.1^e^
10	1.72, m	26.9, CH_2_	1.73, m	26.3, CH_2_
11	1.60, m	29.9, CH_2_	1.61, m	29.9, CH_2_
12	3.20, t (6.9)	40.0, CH_2_	3.20, t (6.9)	39.6, CH_2_
1′		173.3, C		173.4, C
2′	1.91, s	22.6, CH_3_	1.90, s	22.5, CH_3_
7-OMe	3.85, s	56.4, CH_3_	3.95, s	57.0, CH_3_

^a^Measured at 600 MHz

^b^Measured at 150 MHz

^c^Measured at 800 MHz

^d^Measured at 200 MHz

^e^Detected by HMBC

^1^H–^1^H COSY correlations (Fig. 2) exhibited two partial structures comprising C-2 to C-3 and C-10 to C-12. On the basis of the HMBC correlations from H_2_-3 to C-1 and C-4a, H_2_-4 to C-5 and C-8a, H-5 to C-7 and C-8a, H-8 to C-1, C-4a, and C-6, and 7-OMe to C-7, a 6-hydroxy-7-methoxy-3,4-dihydroisoquinoline fragment with a substituent group at C-1 was confirmed. The group at C-1 was deduced as 4-acetamidobutyl by the HMBC correlations from H_2_-10 to C-1, H_2_-11 to C-9 and C-1′, and H_3_-2′ to C-1′. Thus, the structure of **2** was determined as 1-(4-acetamidobutyl)-6-hydroxy-7-methoxy-3,4-dihydroisoquinoline and given the common name pipermullesine B.

The crystals for pipermullesine B trifluoroacetate (**2a**) were obtained from methanol. The NMR data of **2a** (Table 2) and the result of its single-crystal X-ray diffraction analysis (Fig. 3) further supported the structure elucidation of **2**.

Pipermullesine C (**3**) yielded a molecular formula of C_22_H_30_N_4_O_4_ with 10 degrees of unsaturation, as deduced by ^13^C NMR (Table 3) and the HREIMS data. A comparison of the NMR data (Tables 2, 3) of **3** with those of **2** indicated that there were signals for one additional 4-acetamidobutyl group [*δ*_C_ 173.2 (C), 40.1 (CH_2_), 29.7 (CH_2_), 28.9 (CH_2_), 25.2 (CH_2_), and 22.6 (CH_3_)] and one more imine (*δ*_C_ 168.8) in **3**.

**Table 3:** ^1^H and ^13^C NMR Data of **3** and **3a** in CD_3_OD

No.	**3**		**3a**	
	*δ*_H_ (*J* in Hz)^a^	*δ*_C_, type^b^	*δ*_H_ (*J* in Hz)^c^	*δ*_C_, type^d^
1		173.0, C		177.8, C
3	3.62, t (7.5)	41.9, CH_2_	3.80 dd 7.6 7.6	42.1, CH_2_
4	2.95, m	28.4, CH_2_	3.13 dd 7.6 7.6	27.1, CH_2_
4a		141.1, C		140.4, C
5	6.30, s	117.8, CH	6.78, s	113.5, CH
6		167.5, C		153.8^e^
7		144.1, C		140.8, C
8		145.9, C		146.4, C
8a		101.8, C		107.9, C
9	3.31, overlapped	34.7, CH_2_	3.48 dd 7.6 7.6	35.6, CH_2_
10	1.73, m	27.3, CH_2_	1.75, m	26.6, CH_2_
11	1.63, m	30.2, CH_2_	1.65, m	30.1, CH_2_
12	3.21, m	40.0, CH_2_	3.20 dd 7.1 7.1	39.9, CH_2_
1′		173.2, C		173.3, C
2′	1.90, s	22.6, CH_3_	1.92, s	22.6, CH_3_
1″		168.8, C		171.3, C
2″	2.98, m	28.9, CH_2_	3.06 dd 7.4 7.4	28.9, CH_2_
3″	1.90, overlapped	25.2, CH_2_	1.94, m	24.9, CH_2_
4″	1.63, m	29.7, CH_2_	1.65, m	29.8, CH_2_
5″	3.21, m	40.1, CH_2_	3.23 dd 7.1 7.1	39.9, CH_2_
1″′		173.2, C		173.3, C
2″′	1.93, s	22.6, CH_3_	1.89, s	22.6, CH_3_

^a^Measured at 500 MHz

^b^Measured at 125 MHz

^c^Measured at 600 MHz

^d^Measured at 150 MHz

^e^Detected by HMBC

On the basis of 2D NMR correlations (Fig. 2), a 1-(4-acetamidobutyl)-6-hydroxy-3,4-dihydroisoquinoline moiety was determined. One more ring is needed to meet the unsaturation, and the ring was deduced as an oxazole ring attached to C-7 and C-8 by comparison of the NMR data with those of benzoxazoles in the literature [15, 16]. The additional 4-acetamidobutyl group was located at C-1′′ of the oxazole ring by the HMBC correlation from H-3′′ to C-1′′. Thus, the structure of **3** (pipermullesine C) was determined.

Fortunately, the crystals for pipermullesine C trifluoroacetate (**3a**) were also obtained from methanol. The NMR data of **3a** (Table 3) and the result of its single-crystal X-ray diffraction analysis (Fig. 3) confirmed the chemical structure of **3**.

The molecular formula of pipermullamide A (**4**), C_18_H_28_N_2_O_3_, was determined by ^13^C NMR data (Table 4) and an HREIMS ion at 320.2102 [M]^+^ (calcd for C_18_H_28_N_2_O_3_, 320.2100), indicating 6 degrees of unsaturation. The ^1^H NMR data (Table 2) indicated one monosubstituted phenyl ring [*δ*_H_ 7.31 (2H, d, *J *= 7.4 Hz), 7.25 (2H, dd, *J *= 7.4, 7.4 Hz), and 7.17 (1H, dd, *J *= 7.4, 7.4 Hz)], three *N*-methyl groups [*δ*_H_ 2.85 (9H, s)], and two methyl groups [*δ*_H_ 0.98 (3H, d, *J *= 5.9 Hz) and 0.95 (3H, d, *J *= 6.3 Hz)]. By comparing the NMR data of **4** with those of phenylalanine and leucine trimethylbetaine [17, 18], compound **4** might comprise the two fragments, which was confirmed through its ^1^H–^1^H COSY and HMBC correlations (Fig. 2). The amino of phenylalanine was acylated by the carboxyl group of leucine trimethylbetaine according to the HMBC correlation from H-8′ to C-1.

**Table 4:** ^1^H and ^13^C NMR Data of **4**–**6** in CD_3_OD

No.	**4**		**5**		**6**	
	*δ*_H_ (*J* in Hz)^a^	*δ*_C_, type^*b*^	*δ*_H_ (*J* in Hz)^a^	*δ*_C_, type^*b*^	*δ*_H_ (*J* in Hz)^c^	*δ*_C_, type^d^
1		166.2, C		164.9, C		165.0, C
2	3.82, dd (12.4, 2.1)	75.1, CH	3.78, br s	79.1, CH	3.68, d (2.5)	80.6, CH
3	1.96, m 1.57, m	36.2, CH_2_	2.11, m	34.1, CH	2.42, m	27.8, CH
4	1.56, m	25.9, CH	1.61, m 1.51, m	30.6, CH_2_	1.03, d (6.7)	20.2, CH_3_
5	0.95, d (6.3)	24.2, CH_3_	1.01, dd (7.3, 7.3)	12.2, CH_3_	1.23, d (7.0)	23.6, CH_3_
6	0.98, d (5.9)	21.5, CH_3_	1.01, d (6.7)	17.6, CH_3_		
1′		140.0, C		139.9, C		139.9, C
2′,6′	7.31, d (7.4)	130.6, CH	7.29, d (7.4)	130.8, CH	7.29, d (7.4)	130.7, CH
3′,5′	7.25, dd (7.4, 7.4)	129.4, CH	7.23, dd (7.4, 7.4)	129.2, CH	7.24, dd (7.4, 7.4)	129.2, CH
4′	7.17, dd (7.4, 7.4)	127.6, CH	7.15, dd (7.4, 7.4)	127.4, CH	7.15, dd (7.4, 7.4)	127.4, CH
7′	3.41, dd (14.1, 4.4) 2.91, dd (14.1, 10.7)	39.8, CH_2_	3.36, dd (13.9, 4.4) 2.86, dd (13.9, 10.2)	40.2, CH_2_	3.38, dd (13.9, 4.3) 2.85, dd (13.9, 10.3)	40.1, CH_2_
8′	4.71, dd (10.7, 4.4)	57.6, CH	4.68, dd (10.2, 4.4)	57.2, CH	4.70, dd (10.3, 4.3)	57.1, CH
9′		177.2, C		177.2, C		177.2, C
NMe	2.85, s	52.4, CH_3_	2.98, s	52.8, CH_3_	2.96, s	52.9, CH_3_

^a^Measured at 600 MHz

^b^Measured at 150 MHz

^c^Measured at 400 MHz

^d^Measured at 100 MHz

Natural amino acids generally have an l configuration. Compound **10** from the plant is also a derivative of l-phenylalanine. Accordingly, compound **4** (pipermullamide A) was elucidated as l-(*N*,*N*,*N*-trimethyl)leucyl-l-phenylalanine.

The molecular formulae of pipermullamides B to F (**5–9**) were determined as C_18_H_28_N_2_O_3_, C_17_H_26_N_2_O_3_, C_20_H_29_N_3_O_3_, C_20_H_29_N_3_O_3_, and C_19_H_27_N_3_O_3_, respectively, by ^13^C NMR data (Tables 4, 5) and HRMS analysis. According to ^1^H–^1^H COSY and HMBC correlations (Fig. 2), compounds **5**–**9** were determined as l-(*N*,*N*,*N*-trimethyl)isoleucyl-l-phenylalanine (pipermullamide B, **5**), l-(*N*,*N*,*N*-trimethyl)valyl-l-phenylalanine (pipermullamide C, **6**), l-(*N*,*N*,*N*-trimethyl)leucyl-l-tryptophan (pipermullamide D, **7**), l-(*N*,*N*,*N*-trimethyl)isoleucyl-l-tryptophan (pipermullamide E, **8**), and l-(*N*,*N*,*N*-trimethyl)valyl-l-tryptophan (pipermullamide F, **9**), respectively.

**Table 5:** ^1^H and ^13^C NMR Data of **7**–**9** in CD_3_OD

No.	**7**		**8**		**9**	
	*δ*_H_ (*J* in Hz)^a^	*δ*_C_, type^b^	*δ*_H_ (*J* in Hz)^a^	*δ*_C_, type^c^	*δ*_H_ (*J* in Hz)^d^	*δ*_C_, type^c^
1		166.0, C		164.8, C		164.9, C
2	3.71, dd (11.8, 1.8)	75.1, CH	3.59, br s	79.3, CH	3.59, d (2.8)	80.6, CH
3	1.93, m 1.54, m	36.1, CH_2_	2.08, m	34.1, CH	2.36, m	27.8, CH
4	1.54, m	25.9, CH	1.58, m 1.51, m	30.6, CH_2_	1.01, d (6.7)	20.2, CH_3_
5	0.93, d (6.0)	24.1, CH_3_	0.99, overlapped	12.1, CH_3_	1.20, d (7.0)	23.5, CH_3_
6	0.95, d (5.8)	21.6, CH_3_	0.99, overlapped	17.4, CH_3_		
2′	7.15, s	124.5, CH	7.13, s	124.5, CH	7.14, s	124.5, CH
3′		112.4, C		112.4, C		112.4, C
3′a		129.1, C		129.4, C		129.3, C
4′	7.66, br d (7.9)	119.8, CH	7.66, br d (7.9)	119.8, CH	7.67, br d (7.8)	119.8, CH
5′	7.01, ddd (7.9, 7.0, 0.8)	119.7, CH	7.00, ddd (7.9, 6.9, 0.7)	119.6, CH	7.00, ddd (7.8, 7.5, 1.0)	119.6, CH
6′	7.07, ddd (8.1, 7.0, 0.8)	122.4, CH	7.06, ddd (8.1, 6.9, 0.7)	122.2, CH	7.06, ddd (8.0, 7.5, 1.0)	122.3, CH
7′	7.30, br d (8.1)	112.2, CH	7.28, br d (8.1)	112.1, CH	7.28, br d (8.0)	112.1, CH
7′a		137.8, C		137.8, C		137.8, C
8′	3.51, dd (15.0, 4.6) 3.15, dd (15.0, 9.9)	29.3, CH_2_	3.48, dd (14.9, 4.4) 3.11, dd (14.9, 9.5)	29.6, CH_2_	3.49, dd (14.8, 4.3) 3.10, dd (14.8, 9.8)	29.6, CH_2_
9′	4.80, dd (9.9, 4.6)	57.2, CH	4.75, dd (9.5, 4.4)	56.9, CH	4.77, dd (9.8, 4.3)	56.9, CH
10′		177.8, C		177.9, C		177.9, C
NMe	2.69, s	52.3, CH_3_	2.84, s	52.5, CH_3_	2.80, s	52.7, CH_3_

^a^Measured at 800 MHz

^b^Measured at 100 MHz

^c^Measured at 125 MHz

^d^Measured at 500 MHz

The known compounds (+)-phenylalanine betaine (**10**) [19], (-)-mangochinine (**11**) [20], xylopinidine (**12**) [21], (-)-oblongine (**13**) [22], pellitorine (**14**) [23], (2*E*,4*E*)-*N*-isobutyl-2,4-dodecadienamide (**15**) [24], retrofractamide A (**16**) [23], guineensine (**17**) [23], brachystamide B (**18**) [25], retrofractamide C (**19**) [26], sarmentine (**20**) [27], 3-(3,4-dimethoxyphenyl)propanoylpyrrole (**21**) [28], *N*-*trans*-feruloyltyramine (**22**) [29], (-)-machilusin (**23**) [30], galgravin (**24**) [31], (-)-nectandrin A (**25**) [32], methyl 3-(3,4-dimethoxyphenyl)propanoate (**26**) [33], piperic acid (**27**) [34], methyl piperate (**28**) [34], methyl (2*E*,4*E*)-7-(1,3-benzodioxol-5-yl)hepta-2,4-dienoate (**29**) [35], (-)-blumenol B (**30**) [36], (-)-T-muurolol (**31**) [37], *trans*-phytol (**32**) [38], α-tocopherolquinone (**33**) [39], γ-tocopherol (**34**) [40], stigmast-4-ene-3,6-dione (**35**) [41], (22*E*)-stigmasta-4,22-diene-3,6-dione (**36**) [42], and (22*E*)-stigmasta-4,6,8(14),22-tetraen-3-one (**37**) [43] were determined by comparing the NMR data of **10**–**37** and the optical rotation values of **10**–**13**, **23**–**25**, **30**, and **31** with those reported in the literature.

### In Vitro Platelet Aggregation Assay

All isolates (**1**–**37**) were evaluated for inhibitory activity against platelet aggregation induced by thrombin (IIa) or PAF. As shown in Tables 6 and 7, compounds **2**, **3**, **5**, **14**, **27**, **33**, and **34** possessed weak inhibitory effects on the aggregation of rabbit platelets induced by thrombin (IIa) (1 U/mL) with inhibition rates from 11.5 to 22.2% at a concentration of 300 µg/mL. Compounds **11**, **14**, **15**, **20**, and **25** showed weak inhibitory activity against the rabbit platelet aggregation induced by PAF (0.4 µg/mL) with inhibition rates from 16.8% to 36.4% at a concentration of 300 µg/mL, while (-)-mangochinine (**11**), pellitorine (**14**), and (2*E*,4*E*)-*N*-isobutyl-2,4-dodecadienamide (**15**) have IC_50_ values of 470.3 µg/mL, 614.9 µg/mL, and 579.7 µg/mL, respectively. The antiplatelet activity of (-)-mangochinine and (2*E*,4*E*)-*N*-isobutyl-2,4-dodecadienamide was reported for the first time. The other tested compounds were inactive.

**Table 6:** The inhibitory effect of all compounds on aggregation of rabbit platelet induced by Thrombin (IIa) (1 U/mL)^a^

Compound	Concentration (µg/mL)	Inhibition (%)^b^
**2**	300	19.0 ± 9.6
**3**	300	17.0 ± 3.2
**5**	300	11.5 ± 7.9
**14**	300	22.2 ± 12.1
**27**	300	21.9 ± 11.3
**33**	300	14.8 ± 8.5
**34**	300	12.8 ± 9.4
Bivalirudin^c^	50	98.8 ± 1.0

^a^The inhibition of other tested compounds (**1**, **4**, **6**–**13**, **15**–**26**, **28**–**32**, and **35**–**37**) was less than 10% at the concentration of 300 µg/mL

^b^Mean ± SD, *n* = 3

^c^Positive control

**Table 7:** The inhibitory effect of all compounds on rabbit platelet aggregation induced by PAF (0.4 µg/mL)^a^

Compound	Concentration (µg/mL)	Inhibition (%)^b^
**11** ^c^	900	84.9 ± 7.0
	600	66.8 ± 3.9
	300	36.4 ± 3.1
	150	21.9 ± 6.6
	75	10.7 ± 8.5
**14** ^c^	800	68.1 ± 12.6
	600	51.2 ± 12.7
	300	20.4 ± 7.1
	150	10.9 ± 0.6
	75	6.0 ± 2.6
**15** ^c^	900	73.3 ± 14.6
	600	50.4 ± 6.7
	300	33.6 ± 5.1
	150	23.9 ± 9.6
	75	18.3 ± 7.4
**20**	300	16.8 ± 6.7
**25**	300	17.0 ± 7.6
Ginkgolide B^d^	25	90.2 ± 10.4
	12.5	56.9 ± 11.2
	6.25	33.9 ± 9.3
	3.125	19.5 ± 9.6
	1.5625	9.0 ± 5.2

^a^The inhibition of other tested compounds (**1**–**10**, **12**, **13**, **16**–**19**, **21**–**24**, and **26**–**37**) was less than 10% at the concentration of 300 µg/mL

^b^mean ± SD, *n* = 3

^c^The IC_50_ values of **11**, **14**, and **15** was 470.3 ± 51.6 µg/mL, 614.9 ± 113.5 µg/mL, and 579.7 ± 139.1 µg/mL, respectively

^d^Positive control (IC_50_ = 12.2 ± 2.8 µg/mL)

More than fifty antiplatelet compounds, mainly including alkaloids and amides, lignans and neolignans, and phenylpropanoids, have been isolated from the *Piper* genus [44]. Pellitorine is a very interesting compound with various biological activities, such as an inhibitory effect on platelet aggregation induced by arachidonic acid (IC_50_ = 53.0 μg/mL) [45], antituberculosis activity (MIC = 25 µg/mL) [46], antifungal activity against *Cryptococcus neoformans* (IC_50_ = 7.7 μg/mL) [47], and α-glucosidase-I enzyme inhibitory activity (IC_50_ = 34.39 μg/mL) [48]. Although its in vitro activity against platelet aggregation is weak, the amide shows strong in vivo anticoagulant activities at a dose of 4.5 μg/mouse or 9.0 μg/mouse [49]. It is worthwhile to conduct further in vivo antithrombotic studies of pellitorine along with (-)-mangochinine and (2*E*,4*E*)-*N*-isobutyl-2,4-dodecadienamide.

## Experimental Section

### General Experimental Procedures

The instruments and materials for isolation and identification of compounds from the herb were presented in Supplementary Material.

### Plant Material

The aerial parts of *Piper mullesua* Buch.-Ham. ex D. Don (Piperaceae) were collected from Mengyuan Village (E101°22′01″, N21°45′22″), Guanlei Town, Mengla County, Xishuangbanna of Yunnan Province, People’s Republic of China, in July 2014, and identified by one of the authors (C.-L.L.). A voucher specimen (No. 201401) was deposited at the Key Laboratory of Economic Plants and Biotechnology, Kunming Institute of Botany, Chinese Academy of Sciences.

### Extraction and Isolation

The air-dried, powdered *P. mullesua* plant (1.8 kg) was exhaustively extracted with MeOH (4 × 10 L) at room temperature. The MeOH extracts (92.5 g) were suspended in H_2_O and further partitioned with petroleum ether and CHCl_3_. The petroleum ether-soluble part (31.2 g) and CHCl_3_-soluble part (4.8 g) were combined (36.0 g, part B) according to the testing results of thin-layer chromatography. The water phase was partitioned by D101 resin column chromatography to obtain the water-eluted part (discarded) and 95% EtOH-eluted part (7.2 g, part A).

Part A was subjected to column chromatography (silica gel; CHCl_3_/MeOH, 10:1 → 0:1, v/v) to yield three fractions (A1–A3). Fraction A1 was separated on an RP-18 silica gel column eluted with MeOH/H_2_O (10% → 100%). The 40% MeOH-eluted portion was purified by Sephadex LH-20 column chromatography (MeOH) and semi-preparative HPLC (Aligent Zorbax SB-C_18_ column, 10 × 250 mm, 2 mL/min) to obtain **3** (6.5 mg, MeOH/H_2_O, 80:20, *t*_R_ = 12.150 min) and **11** (6.9 mg, MeCN/H_2_O, 15:85, *t*_R_ = 5.190 min).

Fraction A2 was separated on an RP-18 silica gel column eluted with MeOH/H_2_O (10% → 100%) to yield two main subfractions. The 5% MeOH-eluted portion was purified by Sephadex LH-20 column chromatography (MeOH) to yield two main subfractions (A2-1-1 and A2-1-2). Subfraction A2-1-1 was performed on preparative TLC (CHCl_3_/MeOH, 3:1) to obtain **2** (4.3 mg). Subfraction A2-1-2 was recrystallized from MeOH to obtain **10** (23.5 mg). The 30% MeOH-eluted portion was purified by Sephadex LH-20 column chromatography (MeOH) and semipreparative HPLC [Welch Ultimate AQ-C_18_ column, 5.0 μm, *ϕ* 4.6 × 300 mm, MeCN/H_2_O (containing 0.05% TFA), 20:80, 1 mL/min] to obtain **13** (2.0 mg, *t*_R_ = 5.796 min).

Fraction A3 was separated on an RP-18 silica gel column eluted with MeOH/H_2_O (10% → 100%) to yield two main subfractions. The 15% MeOH-eluted portion was purified by Sephadex LH-20 column chromatography (MeOH) and semipreparative HPLC (Aligent Zorbax SB-C_18_ column, 10 × 250 mm, MeOH/H_2_O, 20:80, 2 mL/min) to obtain **7** (4.5 mg, *t*_R_ = 14.369 min), **6** (8.0 mg, *t*_R_ = 15.398 min), **9** (24.0 mg, *t*_R_ = 17.255 min), **8** (38.4 mg, *t*_R_ = 22.038 min), **5** (27.3 mg, *t*_R_ = 23.055 min), and **4** (8.5 mg, *t*_R_ = 38.045 min). The 35% MeOH-eluted portion was purified by Sephadex LH-20 column chromatography (MeOH) and semipreparative HPLC [Welch Ultimate AQ-C_18_ column, 5.0 *μ*m, *ϕ* 4.6 × 300 mm, MeCN/H_2_O (containing 0.05% TFA), 20:80, 1 mL/min] to obtain **12** (3.6 mg, *t*_R_ = 9.300 min).

Part B was subjected to column chromatography (silica gel; petroleum ether/acetone, 20:1 → 0:1, v/v) to yield five fractions (B1–B5). Fraction B1 was separated on an RP-18 silica gel column eluted with MeOH/H_2_O (10% → 100%) to yield four main subfractions. The 70% MeOH-eluted portion was purified by Sephadex LH-20 column chromatography (MeOH) and silica gel column chromatography (petroleum ether/acetone, 40:1, v/v) to obtain **28** (3.3 mg) and **29** (4.9 mg). The 85% MeOH-eluted portion was purified by Sephadex LH-20 column chromatography (MeOH) and preparative TLC (petroleum ether/EtOAc, 20:1, v/v) to obtain **32** (13.5 mg). The 90% MeOH-eluted portion was purified by Sephadex LH-20 column chromatography (MeOH) to obtain **31** (2.3 mg) recrystallized from MeOH. The 95% MeOH-eluted portion was purified by Sephadex LH-20 column chromatography (MeOH) and semipreparative HPLC (Aligent Zorbax SB-C_18_ column, 10 × 250 mm, MeOH/H_2_O, 100:0, 2 mL/min) to obtain **34** (6.0 mg, *t*_R_ = 19.574 min) and **37** (1.5 mg, *t*_R_ = 26.868 min).

Fraction B2 was separated on an RP-18 silica gel column eluted with MeOH/H_2_O (10% → 100%) to yield two main subfractions. The 80% MeOH-eluted portion was purified by Sephadex LH-20 column chromatography (MeOH) and semipreparative HPLC (Aligent Zorbax SB-C_18_ column, 10 × 250 mm, 2 mL/min) to obtain **14** (86.7 mg, MeOH/H_2_O, 80:20, *t*_R_ = 16.778 min), **26** (6.4 mg, MeOH/H_2_O, 75:25, *t*_R_ = 8.558 min), **21** (16.0 mg, MeOH/H_2_O, 80:20, *t*_R_ = 9.164 min), and **23** (18.9 mg, MeOH/H_2_O, 80:20, *t*_R_ = 19.593 min). The 90% MeOH-eluted portion was purified by Sephadex LH-20 column chromatography (MeOH) and semipreparative HPLC (Aligent Zorbax SB-C_18_ column, 10 × 250 mm, MeCN/H_2_O, 99:1, 2 mL/min) to obtain **36** (3.4 mg, *t*_R_ = 37.378 min), **35** (4.0 mg, *t*_R_ = 42.351 min), and **33** (5.0 mg, *t*_R_ = 45.179 min).

Fraction B3 was separated on an RP-18 silica gel column eluted with MeOH/H_2_O (10% → 100%) to yield two main subfractions. The 60% MeOH-eluted portion was purified by Sephadex LH-20 column chromatography (MeOH) to obtain **17** (32.6 mg) and **24** (105.7 mg) recrystallized from MeOH. The 70% MeOH-eluted portion was purified by Sephadex LH-20 column chromatography (MeOH) and semipreparative HPLC (Aligent Zorbax SB-C_18_ column, 10 × 250 mm, MeOH/H_2_O, 85:15, 2 mL/min) to obtain **15** (10.3 mg, *t*_R_ = 19.983 min).

Fraction B4 was separated on an RP-18 silica gel column eluted with MeOH/H_2_O (10% → 100%) to yield four main subfractions. The 60% MeOH-eluted portion was purified by Sephadex LH-20 column chromatography (MeOH) to obtain **1 (**169.9 mg) recrystallized from MeOH. The 70% MeOH-eluted portion was isolated by column chromatography (Sephadex LH-20, MeOH; silica gel, petroleum ether/acetone, 30:1, v/v) and further purified by semipreparative HPLC (Agilent Zorbax SB-C_18_ column, 9.4 × 250 mm, 2 mL/min) to yield **20** (8.5 mg, MeOH/H_2_O, 80:20, *t*_R_ = 19.115 min), **25** (3.0 mg, MeOH, 75:25, *t*_R_ = 15.549 min), and **18** (16.0 mg; MeOH/H_2_O, 80:20, *t*_R_ = 15.356 min). The 80% MeOH-eluted portion was purified by Sephadex LH-20 column chromatography (MeOH) obtain **19** (2.0 mg) recrystallized from MeOH. The 90% MeOH/H_2_O-eluted portion was purified by Sephadex LH-20 column chromatography (MeOH) and semipreparative HPLC (Aligent Zorbax SB-C_18_ column, 10 × 250 mm, MeOH/H_2_O, 95:5, 2 mL/min) to obtain **16** (14.7 mg, *t*_R_ = 14.172 min).

Fraction B5 was separated on an RP-18 silica gel column eluted with MeOH/H_2_O (10% → 100%) to yield four main subfractions. The 10% MeOH-eluted portion was purified by column chromatography (Sephadex LH-20, MeOH; preparative TLC, CHCl_3_/MeOH, 5:1, v/v) and semipreparative HPLC (Aligent Zorbax SB-C_18_ column, 10 × 250 mm, MeOH/H_2_O, 40:60, 2 mL/min) to obtain **30** (18.2 mg, *t*_R_ = 18.052 min). The 20% MeOH-eluted portion was purified by Sephadex LH-20 column chromatography (MeOH) to obtain **27** (52.3 mg) recrystallized from MeOH. The 30% MeOH-eluted portion was purified by Sephadex LH-20 column chromatography (MeOH) and semipreparative HPLC (Aligent Zorbax SB-C_18_ column, 10 × 250 mm, MeOH/H_2_O, 50:50, 2 mL/min) to obtain **22 (**18.0 mg, *t*_R_ = 6.746 min).

### Spectroscopic Data of Compounds

#### Pipermullesine A (**1**)

Pale yellow needles (CHCl_3_); mp 90–93 °C; UV (MeOH) *λ*_max_ (log*ε*) 334 (4.22), 243 (4.11), 224 (3.94) nm; IR (KBr) *ν*_max_ 1643, 1595, 1513, 1461, 1439, 1415, 1376, 1345, 1315, 1287, 1269, 1228, 1160, 1141, 1047, 1023, 907, 803 cm^−1^; ^1^H NMR and ^13^C NMR data, see Table 1; ESIMS *m/z* 296 [M + Na]^+^, 569 [2 M + Na]^+^; HREIMS *m/z* 273.0997 [M]^+^ (calcd for C_15_H_15_NO_4_, 273.1001).

Crystal data for pipermullesine A (**1**): C_15_H_15_NO_4_·H_2_O, *M *= 291.30, monoclinic, *a *= 4.9028(8) Å, *b *= 21.159(3) Å, *c *= 13.455(2) Å, *α *= 90.00°, *β *= 92.120(2)°, *γ *= 90.00°, *V *= 1394.9(4) Å^3^, *T *= 100(2) K, space group *P*21*/n*, *Z *= 4, *μ*(MoKα) = 0.105 mm^−1^, 14679 reflections measured, and 3883 independent reflections (*R*_*int*_= 0.0331). The final *R*_*1*_ value was 0.0434 (*I *> 2*σ*(*I*)). The final *wR*(*F*^2^) value was 0.1212 (*I *> 2*σ*(*I*)). The final *R*_*1*_ value was 0.0575 (all data). The final *wR*(*F*^2^) value was 0.1322 (all data). The goodness of fit on *F*^2^ was 1.030. The crystallographic data for the structure of **1** have been deposited in the Cambridge Crystallographic Data Centre (deposition number CCDC 1529565). Copies of the data can be obtained free of charge from the CCDC via www.ccdc.cam.ac.uk.

#### Pipermullesine B (**2**)

Pale yellow powder; UV (MeOH) *λ*_max_ (log*ε*) 402 (3.62), 310 (3.07), 268 (3.43) nm; IR (KBr) *ν*_max_ 3424, 1622, 1511, 1466, 1441, 1368, 1354, 1235, 1208, 1180, 1039 cm^−1^; ^1^H and ^13^C NMR data, see Table 2; ESIMS *m/z* 291 [M + H]^+^, 313 [M + Na]^+^; HREIMS *m/z* 290.1620 [M]^+^ (calcd for C_16_H_22_N_2_O_3_, 290.1630).

#### Pipermullesine C (**3**)

Pale yellow powder; UV (MeOH) *λ*_max_ (log*ε*) 400 (3.62), 310 (3.10), 268 (3.42) nm; IR (KBr) *ν*_max_: 3376, 1721, 1630, 1607, 1590, 1562, 1511, 1462, 1438, 1384, 1337, 1271, 1237, 1211, 1101, 1076, 1037 cm^−1^; ^1^H and ^13^C NMR data, see Table 3; ESIMS *m/z* 415 [M + H]^+^, 437 [M + Na]^+^; HREIMS *m/z* 414.2223 [M]^+^ (calcd for C_22_H_30_N_4_O_4_, 414.2223).

#### Pipermullamide A (**4**)

White solid; $[\alpha ]_{\text{D}}^{25}$− 16.2 (*c* 0.08, MeOH); UV (MeOH) *λ*_max_ (log*ε*) 204 (3.35) nm; ECD Δ*ε* (*c* 0.08, MeOH) +1.35 (217); IR (KBr) *ν*_max_ 3429, 1713, 1626, 1460, 1415, 1384, 1299, 1274, 1126, 1079, 1046 cm^−1^; ^1^H and ^13^C NMR data, see Table 4; ESIMS *m/z* 321 [M + H]^+^, 343 [M + Na]^+^; HREIMS *m/z* 320.2102 [M]^+^ (calcd for C_18_H_28_N_2_O_3_, 320.2100).

#### Pipermullamide B (**5**)

White solid; $[\alpha ]_{\text{D}}^{19}$− 7.6 (*c* 0.18, MeOH); UV (MeOH) *λ*_max_ (log*ε*) 203 (4.00) nm; ECD Δ*ε* (*c* 0.012, MeOH) +1.48 (214); IR (KBr) *ν*_max_ 3442, 1666, 1609, 1494, 1456, 1385, 1312, 1256, 1226, 1091, 1031 cm^−1^; ^1^H and ^13^C NMR data, see Table 4; ESIMS *m/z* 321 [M + H]^+^, 343 [M + Na]^+^; HREIMS *m/z* 320.2102 [M]^+^ (calcd for C_18_H_28_N_2_O_3_, 320.2100).

#### Pipermullamide C (**6**)

White solid; $[\alpha ]_{\text{D}}^{20}{-}33.1$ (*c* 0.09, MeOH); UV (MeOH) *λ*_max_ (log*ε*) 206 (4.07) nm; ECD Δ*ε* (*c* 0.014, MeOH) +2.50 (214); IR (KBr) *ν*_max_ 3426, 1673, 1608, 1494, 1456, 1383, 1315, 1257, 1225, 1096, 1032 cm^−1^; ^1^H and ^13^C NMR data, see Table 4; ESIMS *m/z* 329 [M + Na]^+^; HRESIMS *m/z* 307.2017 [M + H]^+^ (calcd for C_17_H_27_N_2_O_3_, 307.2022).

#### Pipermullamide D (**7**)

White solid; $[\alpha ]_{\text{D}}^{21}{-}13.7$ (*c* 0.08, MeOH); UV (MeOH) *λ*_max_ (log*ε*) 281 (3.47), 222 (4.28), 206 (4.16) nm; ECD Δ*ε* (*c* 0.037, MeOH) +1.40 (233), -1.15 (222), -0.94 (214); IR (KBr) *ν*_max_ 3426, 1674, 1611, 1488, 1459, 1384, 1259, 1228, 1127, 1101 cm^−1^; ^1^H and ^13^C NMR data, see Table 5; ESIMS *m/z* 360 [M + H]^+^, 382 [M + Na]^+^; HRESIMS *m/z* 360.2288 [M + H]^+^ (calcd for C_20_H_30_N_3_O_3_, 360.2287).

#### Pipermullamide E (**8**)

White solid; $[\alpha ]_{\text{D}}^{21} {-}32.2$ (*c* 0.06, MeOH); UV (MeOH) *λ*_max_ (log*ε*) 398 (1.74), 282 (3.09), 220 (3.90), 205 (3.89) nm; ECD Δ*ε* (*c* 0.028, MeOH) +1.21 (233), +1.87 (225), -0.51 (220), -3.34 (200); IR (KBr) *ν*_max_ 3428, 1681, 1619, 1452, 1422, 1384, 1209, 1139, 1046 cm^−1^; ^1^H and ^13^C NMR data, see Table 5; ESIMS *m/z* 360 [M + H]^+^, 382 [M + Na]^+^; HRESIMS *m/z* 360.2293 [M + H]^+^ (calcd for C_20_H_30_N_3_O_3_, 360.2287).

#### Pipermullamide F (**9**)

White solid; $[\alpha ]_{\text{D}}^{20}{-}12.2$ (*c* 0.21, MeOH); UV (MeOH) *λ*_max_ (log*ε*) 281 (3.53), 221 (4.31), 206 (4.15) nm; ECD Δ*ε* (*c* 0.013, MeOH) +1.30 (228), -1.80 (212), -4.48 (200); IR (KBr) *ν*_max_ 3415, 3266, 1672, 1603, 1491, 1459, 1384, 1255, 1229, 1098, 961, 747 cm^−1^; ^1^H and ^13^C NMR data, see Table 5; ESIMS *m/z* 346 [M + H]^+^, 368 [M + Na]^+^; HRESIMS *m/z* 346.2128 [M + H]^+^ (calcd for C_19_H_28_N_3_O_3_, 346.2131).

### Preparation of Pipermullesine B Trifluoroacetate (2a)

Compound **2** (1.6 mg, 0.00551 mmol) was performed on semipreparative HPLC [Welch Ultimate AQ-C_18_ column, 5.0 μm, *ϕ* 4.6 × 300 mm, MeCN/H_2_O (containing 0.05% TFA), 20:80, 1.0 mL/min] to obtain **2a** (2.0 mg, *t*_R_ = 6.344 min; 0.00516 mmol, 94% yield): pale yellow needles (MeOH); mp 142–145 °C; ^1^H and ^13^C NMR data, see Table 2.

Crystal data for pipermullesine B trifluoroacetate (**2a**): C_16_H_23_N_2_O_3_·C_2_F_3_O_2_, *M *= 404.38, *a *= 7.6163(3) Å, *b *= 8.8383(3) Å, *c *= 14.8434(5) Å, *α *= 82.5370(10)°, *β *= 89.4170(10)°, *γ *= 74.5720(10)°, *V *= 954.71(6) Å^3^, *T *= 100(2) K, space group *P*-1, *Z *= 2, *μ*(CuKα) = 1.046 mm^−1^, 13115 reflections measured, 3381 independent reflections (*R*_*int*_= 0.0623). The final *R*_*1*_ value was 0.1027 (*I *> 2*σ*(*I*)). The final *wR*(*F*^2^) value was 0.2945 (*I *> 2*σ*(*I*)). The final *R*_*1*_ value was 0.1048 (all data). The final *wR*(*F*^2^) value was 0.2990 (all data). The goodness of fit on *F*^2^ was 1.412. The crystallographic data for the structure of **2a** have been deposited in the Cambridge Crystallographic Data Centre (deposition number CCDC 1529558). Copies of the data can be obtained free of charge from the CCDC via www.ccdc.cam.ac.uk.

### Preparation of Pipermullesine C Trifluoroacetate (3a)

Compound **3** (4.0 mg, 0.00965 mmol) was performed on semipreparative HPLC [Welch Ultimate AQ-C_18_ column, 5.0 μm, *ϕ* 4.6 × 300 mm, MeCN/H_2_O (containing 0.05% TFA), 20:80, 1.0 mL/min] to obtain **3a** (4.5 mg, *t*_R_ = 7.460 min; 0.00880 mmol, 91% yield): pale yellow needles (MeOH); mp 157–159 °C; ^1^H and ^13^C NMR data, see Table 3.

Crystal data for pipermullesine C trifluoroacetate (**3a**): C_22_H_31_N_4_O_4_·C_2_F_3_O_2_, *M *= 528.53, *a *= 8.5097(10) Å, *b *= 12.3631(15) Å, *c *= 12.8583(15) Å, *α *= 68.354(2)°, *β *= 78.084(2)°, *γ *= 80.814(2)°, *V *= 1225.1(3) Å^3^, *T *= 100(2) K, space group *P*-1, *Z *= 2, *μ*(MoKα) = 0.118 mm^−1^, 13522 reflections measured, 6727 independent reflections (*R*_*int*_= 0.0383). The final *R*_*1*_ values were 0.0528 (*I *> 2*σ*(*I*)). The final *wR*(*F*^2^) value was 0.1229 (*I *> 2*σ*(*I*)). The final *R*_*1*_ value was 0.0879 (all data). The final *wR*(*F*^2^) value was 0.1414 (all data). The goodness of fit on *F*^2^ was 1.020. The crystallographic data for the structure of **3a** have been deposited in the Cambridge Crystallographic Data Centre (deposition number CCDC 1589949). Copies of the data can be obtained free of charge from the CCDC via www.ccdc.cam.ac.uk.

### In vitro Platelet Aggregation Assay

The inhibitory effects of compounds against rabbit platelet aggregation induced by PAF or Thrombin (IIa) were evaluated according to the published methods [50–53]. The details were presented in Supplementary Material.

## Conclusion

Thirty-seven compounds were isolated from the folk Chinese medicine *Piper mullesua* with the “Huayu” function associated with the antiplatelet therapies. The antiplatelet compounds, especially (-)-mangochinine, pellitorine, and (2*E*,4*E*)-*N*-isobutyl-2,4-dodecadienamide, might be scientific evidence to support the traditional use of the plant as folk medicine. In order to make better use of the folk medicine to serve for human health, further research needs to be conducted on bioguided isolation of compounds from the plant, based on both in vitro and in vivo bioassay testing.

## Electronic supplementary material

Below is the link to the electronic supplementary material.

Supplementary material 1 (DOC 5719 kb)
