# Supplementary material for: Amides, Isoquinoline Alkaloids and Dipeptides from the Aerial Parts of Piper mullesua
Source: Nat Prod Bioprospect. 2018 Aug 2;8(6):419–30. doi: 10.1007/s13659-018-0180-z (PMC6224811; doi:10.1007/s13659-018-0180-z)
Supplement: Supplementary file 1 — Supplementary material 1 (DOC 5719 kb) [file 13659_2018_180_MOESM1_ESM.doc]

Supplementary Material for

Amides, Isoquinoline Alkaloids and Dipeptides from the Aerial Parts of *Piper mullesua*

Meng-Yuan Xia^1,2^ **∙** Jun Yang^1,2^ **∙** Pan-Hua Zhang^1^ **∙** Xiao-Nian Li^3^ **∙** Ji-Feng Luo^1^**∙**

Chun-Lin Long^1,4^ **∙** Yue-Hu Wang^1,2^

Meng-Yuan Xia and Jun Yang contributed equally to this work.

**Electronic supplementary material** The online version of this article (doi: ) contains supplementary material, which is available to authorized users.

🖂 Chun-Lin Long

long@mail.kib.ac.cn

🖂 Yue-Hu Wang

wangyuehu@mail.kib.ac.cn

^1^ Key Laboratory of Economic Plants and Biotechnology and the Yunnan Key Laboratory for Wild Plant Resources, Kunming Institute of Botany, Chinese Academy of Sciences, Kunming 650201, People’s Republic of China

^2^ Southeast Asia Biodiversity Research Institute, Chinese Academy of Sciences, Yezin, Nay Pyi Taw 05282, Myanmar

^3^ State Key Laboratory of Phytochemistry and Plant Resources in West China, Kunming Institute of Botany, Chinese Academy of Sciences, Kunming 650201, People’s Republic of China

^4^  College of Life and Environmental Sciences, Minzu University of China, Beijing 100081, People’s Republic of China

**Contents**

| ***General experimental procedures*** |
| --- |
| ***In vitro platelet aggregation assay*** |
| **Scheme S1. Extraction procedures** |
| **Fig. S1.** ^1^H NMR spectrum of **1** (CDCl_3_, 500 MHz). |
| **Fig. S2.** ^13^C NMR spectrum of **1** (CDCl_3_, 125 MHz). |
| **Fig. S3.** HSQC spectrum of **1**. |
| **Fig. S4.** ^1^H‒^1^H COSY spectrum of **1**. |
| **Fig. S5.** HMBC spectrum of **1**. |
| **Fig. S6.** ROESY spectrum of **1**. |
| **Fig. S7.** HREIMS spectrum of **1.** |
| **Fig. S8.** ^1^H NMR spectrum of **2** (CD_3_OD, 600 MHz). |
| **Fig. S9.** ^13^C NMR spectrum of **2** (CD_3_OD, 150 MHz). |
| **Fig. S10.** HSQC spectrum of **2**. |
| **Fig. S11.** ^1^H‒^1^H COSY spectrum of **2**. |
| **Fig. S12.** HMBC spectrum of **2**. |
| **Fig. S13.** ROESY spectrum of **2**. |
| **Fig. S14.** HREIMS spectrum of **2.** |
| **Fig. S15.** ^1^H NMR spectrum of **2a** (CD_3_OD, 800 MHz). |
| **Fig. S16.** ^13^C NMR spectrum of **2a** (CD_3_OD, 200 MHz). |
| **Fig. S17.** HSQC spectrum of **2a**. |
| **Fig. S18.** ^1^H‒^1^H COSY spectrum of **2a**. |
| **Fig. S19.** HMBC spectrum of **2a**. |
| **Fig. S20.** ROESY spectrum of **2a.** |
| **Fig. S21.** ^1^H NMR spectrum of **3** (CD_3_OD, 500 MHz). |
| **Fig. S22.** ^13^C NMR spectrum of **3** (CD_3_OD, 120 MHz). |
| **Fig. S23.** HSQC spectrum of **3**. |
| **Fig. S24.** ^1^H‒^1^H COSY spectrum of **3**. |
| **Fig. S25.** HMBC spectrum of **3**. |
| **Fig. S26.** ROESY spectrum of **3**. |
| **Fig. S27.** HREIMS spectrum of **3**. |
| **Fig. S28.** ^1^H NMR spectrum of **4** (CD_3_OD, 600 MHz). |
| **Fig. S39.** ^13^C NMR spectrum of **4** (CD_3_OD, 150 MHz). |
| **Fig. S30.** HSQC spectrum of **4**. |
| **Fig. S31.** ^1^H‒^1^H COSY spectrum of **4**. |
| **Fig. S32.** HMBC spectrum of **4**. |
| **Fig. S33.** ROESY spectrum of **4**. |
| **Fig. S34.** HREIMS spectrum of **4**. |
| **Fig. S35.** ECD spectrum of **4**. |
| **Fig. S36.** ^1^H NMR spectrum of **5** (CD_3_OD, 600 MHz). |
| **Fig. S37.** ^1^H NMR spectrum of **5** (CD_3_OD, 150 MHz). |
| **Fig. S38.** HSQC spectrum of **5**. |
| **Fig. S39.** ^1^H‒^1^H COSY spectrum of **5**. |
| **Fig. S40.** HMBC spectrum of **5**. |
| **Fig. S41.** ROESY spectrum of **5**. |
| **Fig. S42.** HREIMS spectrum of **5**. |
| **Fig. S43.** ECD spectrum of **5**. |
| **Fig. S44.** ^1^H NMR spectrum of **6** (CD_3_OD, 400 MHz). |
| **Fig. S45.** ^1^H NMR spectrum of **6** (CD_3_OD, 100 MHz). |
| **Fig. S46.** HSQC spectrum of **6**. |
| **Fig. S47.** ^1^H‒^1^H COSY spectrum of **6**. |
| **Fig. S48.** HMBC spectrum of **6**. |
| **Fig. S49.** HRESIMS spectrum of **6**. |
| **Fig. S50.** ECD spectrum of **6**. |
| **Fig. S51.** ^1^H NMR spectrum of **7** (CD_3_OD, 800 MHz). |
| **Fig. S52.** ^13^C NMR spectrum of **7** (CD_3_OD, 100 MHz). |
| **Fig. S53.** HSQC spectrum of **7**. |
| **Fig. S54.** ^1^H‒^1^H COSY spectrum of **7**. |
| **Fig. S55.** HMBC spectrum of **7**. |
| **Fig. S56.** ROESY spectrum of **7**. |
| **Fig. S57.** HRESIMS spectrum of **7**. |
| **Fig. S58.** ECD spectrum of **7**. |
| **Fig. S59.** ^1^H NMR spectrum of **8** (CD_3_OD, 800 MHz). |
| **Fig. S60.** ^1^H NMR spectrum of **8** (CD_3_OD, 125 MHz). |
| **Fig. S61.** HSQC spectrum of **8**. |
| **Fig. S62.** ^1^H–^1^H COSY spectrum of **8**. |
| **Fig. S63.** HMBC spectrum of **8**. |
| **Fig. S64.** ROESY spectrum of **8**. |
| **Fig. S65.** HRESIMS spectrum of **8**. |
| **Fig. S66.** ECD spectrum of **8**. |
| **Fig. S67.** ^1^H NMR spectrum of **9** (CD_3_OD, 500 MHz). |
| **Fig. S68.** ^13^C NMR spectrum of **9** (CD_3_OD, 125 MHz). |
| **Fig. S69.** HSQC spectrum of **9**. |
| **Fig. S70.** ^1^H‒^1^H COSY spectrum of **9**. |
| **Fig. S71.** HMBC spectrum of **9**. |
| **Fig. S72.** ROESY spectrum of **9**. |
| **Fig. S73.** HRESIMS spectrum of **9**. |
| **Fig. S74.** ECD spectrum of **9**. |

*General*

The melting points were determined using an X-4 melting point apparatus (Yingyu Yuhua Apparatus Factory, Gongyi, China) and were not corrected. Optical rotations were recorded using a JASCO P-1020 Polarimeter (Jasco Corp., Tokyo, Japan). Ultraviolet (UV) spectra were taken on a Shimadzu UV-2401 PC spectrophotometer (Shimadzu, Kyoto, Japan). Electronic circular dichroism (ECD) spectra were recorded on a Chirascan CD spectrometer (Applied Photophysics Ltd., Leatherhead, UK). Infrared (IR) spectra were measured on a Bruker Tensor 27 FTIR Spectrometer (Bruker Corp., Ettlingen, Germany) with KBr disks. ^1^H and ^13^C Nuclear magnetic resonance (NMR) spectra were collected on a Bruker AM-400, DRX-500, Avance III-600, and Accend™ 800 MHz spectrometers (Bruker Corp., Karlsruhe, Germany) with tetramethylsilane (TMS) as an internal standard. Electrospray ionization mass spectrometry (ESIMS) and high-resolution electrospray ionization mass spectrometry (HRESIMS) analyses were performed on an API QSTAR Pulsar 1 spectrometer (Applied Biosystems/MDS Sciex, Foster City, CA, USA). HREIMS were performed on a Waters AutoSpec Premier p776 spectrometer (Waters, Milford, MA, USA). X-ray diffraction was performed on a Bruker APEX DUO diffractometer using graphite-monochromated CuKα radiation. Silica gel G (80–100 and 300–400 mesh, Qingdao Meigao Chemical Co., Ltd., Qingdao, China), C_18_ silica gel (40–75 μm, Fuji Silysia Chemical Ltd., Aichi, Japan), and Sephadex LH-20 (GE Healthcare Bio-Sciences AB, Uppsala, Sweden) were used for column chromatography, and silica gel GF_254_ (Qingdao Meigao Chemical Co., Ltd.) was used for preparative thin layer chromatography (TLC) as precoated plates. TLC spots were visualized under UV light at 254 nm and by dipping into 5% H_2_SO_4_ in alcohol followed by heating. Semipreparative high-performance liquid chromatography (HPLC) was performed on an Agilent 1200 series pump (Agilent Technologies, Santa Clara, USA) equipped with a diode array detector and a Waters XBridge C_18_ column (5.0 *μ*m, *φ* 10×250 mm) and an Agilent Zorbax SB-C_18_ column (5.0 μm, *φ* 9.4×250 mm).

*In vitro platelet aggregation assay*

Thrombin (IIa), bivalirudin, PAF, and ginkgolide B were purchased from Sigma-Aldrich Corporation (St Louis, Missouri, USA). Japanese big-ear white rabbits were purchased from Chengdu Dashuo Biological Science Co. Ltd., China [certification number SCXK (Chuan) 2013-24]. Turbid metric measurements of platelet aggregation were performed in a Chronolog Model 700 Aggregometer (Chronolog Corporation, Havertown, PA, USA) according to Born’s method (Born, 1962; Küster et al., 1986; Wu et al., 2015; Zhu et al., 2016; Zhuo et al., 2016).

The blood from the central aural artery of rabbits by a butterfly infusion set was anticoagulated with 3.8% sodium citrate (9:1, v/v). Platelet-rich plasma (PRP) was prepared shortly after blood collection by spinning the sample at 180 g for 10 min at 22 °C. The PRP was carefully removed, and the remaining blood was centrifuged at 2400 g for 10 min to obtain platelet poor plasma (PPP). The centrifuge temperature was maintained at 22 °C. The platelet number was adjusted by the addition of PPP to the PRP to achieve a count of 500×10^9^ cells/L. Platelet aggregation studies were completed within 3 h of the preparation of PRP. Immediately after the preparation of PRP, 250 μL was transferred into each of the prepared test tubes, with 250 μL of PPP set as a control. Before the addition of inducers, compounds were incubated with PRP at 37 °C for 5 min. The change of optical density as a result of platelet aggregation was recorded, and the inhibition percentage of the compounds was calculated according to the following formula: inhibition of aggregation (%)=(A−B)/A×100% (A: maximum change in turbidity in the DMSO added; B: maximum change in turbidity in the sample added).

**References**

[1] C. Chen, F.Q. Wang, X. Wang, Z.N. Xia, H. Guang, J. Tradit. Chin. Med. 37, 64-75 (2017)

[2] X.L. Su, W. Su, Y. Wang, X. Ming, Y. Kong, Acta Pharm. Sinica 37, 1208-1217 (2016)

[3] J.X. Zhuo, Y.H. Wang, X.L. Su, R.Q. Mei, J. Yang, Y. Kong, C.L. Long, Nat. Prod. Bioprospect. 6, 161-166 (2016)

[4] Y.H. Wang, S.L. Morris Natschke, J. Yang, H.M. Niu, C.L. Long, K.H. Lee, J. Tradit. Complement. Med. 4, 8-16 (2014)

[5] Editorial Board of "Zhonghua Bencao", Zhonghua Bencao. Shanghai

Scientific and Technological Press: Shanghai. 3, 424–449 ( 1999)

Air-dried *P. mullesua* (2.5 kg) plant (1.8 kg)

partitioned with petroleum ether and CHCl_3_

MeOH extracts (92.5 g)

extracted with MeOH

A(water part,7.2g)

B（PE-soluble and CHCl_3_-soluble parts ,36.0 g）

silica gel; CHCl_3_/MeOH, 10:1→0:1, v/v

A1(1.01 g)

A2(1.51 g)

A3(1.36 g)

**3**,**11**

**2**,**10**,**13**

**4**,**5**,**6**,**7**,**8**,**9,12**

B1(8.85 g)

B2(2.78 g)

B3(3.17 g)

B4(7.71 g)

B5(15.11g)

**28,29**,**31,32**,

**34**,**37**

**14**,**21**,**23**,**26**,

**33**,**35**,**36**

**15**,**17**,**24**

**1,16**,**18**,**19**,

**20**,**25**

**22**,**27**,**30**

silica gel; petroleum ether/acetone, 20:1→0:1, v/v

Scheme S1. Extraction procedures

**Fig. S1.** ^1^H NMR spectrum of **1** (CDCl_3_, 500 MHz).

**Fig. S2.** ^13^C NMR spectrum of **1** (CDCl_3_, 125 MHz).

**Fig. S3.** HSQC spectrum of **1**.

**Fig. S4.** ^1^H–^1^H COSY spectrum of **1**.

**Fig. S5.** HMBC spectrum of **1**.

**Fig. S6.** ROESY spectrum of **1**.


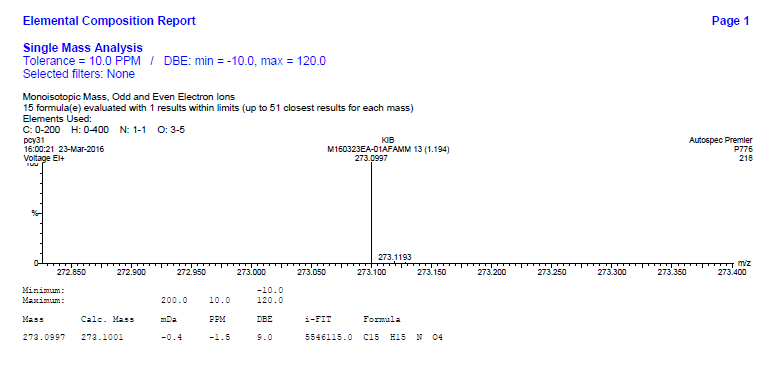


**Fig. S7.** HREIMS spectrum of **1**.

**Fig. S8.** ^1^H NMR spectrum of **2** (CD_3_OD, 600 MHz).

**Fig. S9.** ^13^C NMR spectrum of **2** (CD_3_OD, 150 MHz).

**Fig. S10.** HSQC spectrum of **2**.

**Fig. S11.** ^1^H–^1^H COSY spectrum of **2**.

**Fig. S12.** HMBC spectrum of **2**.

**Fig. S13.** ROESY spectrum of **2**.


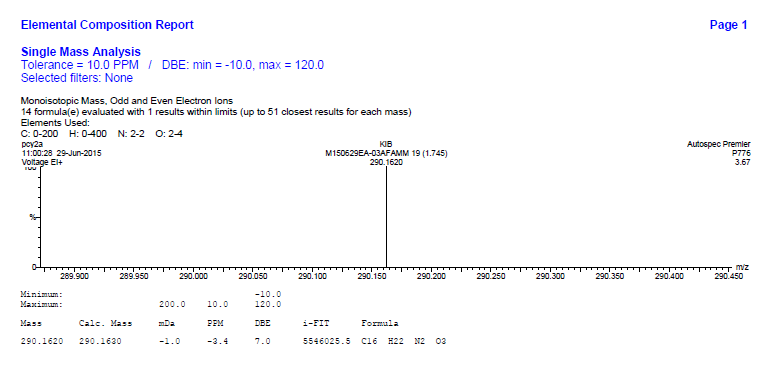


**Fig. S14.** HREIMS spectrum of **2**.

**Fig. S15.** ^1^H NMR spectrum of **2a** (CD_3_OD, 800 MHz).


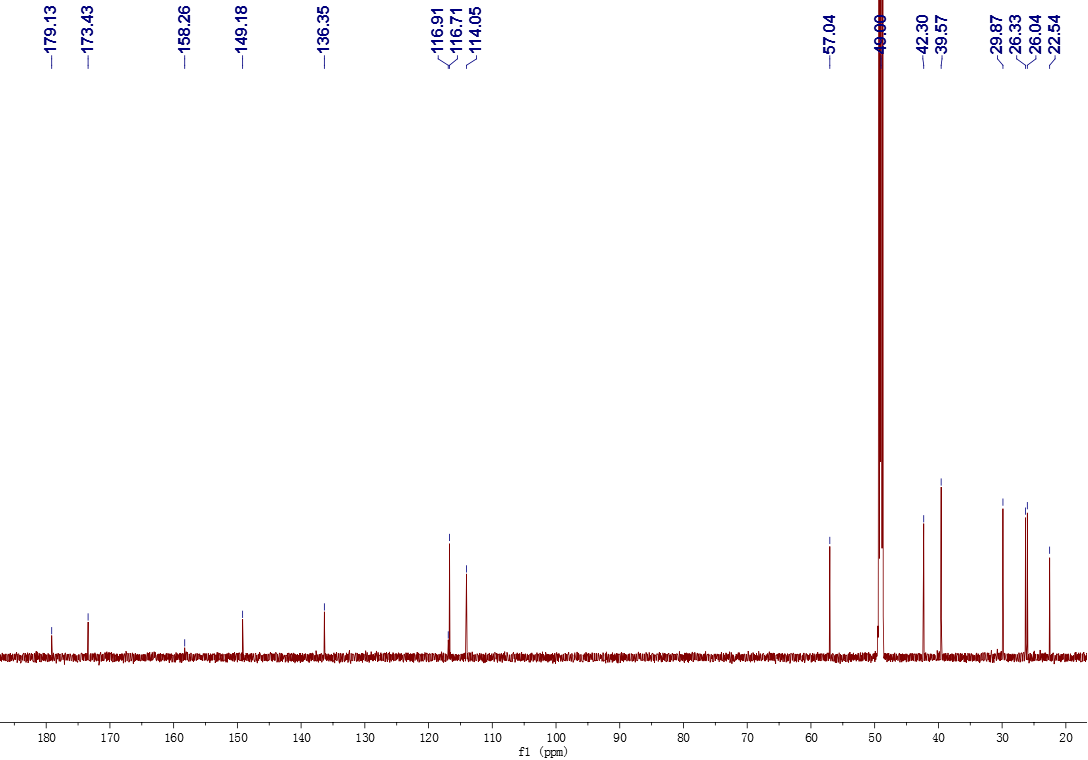


**Fig. S16.** ^13^C NMR spectrum of **2a** (CD_3_OD, 200 MHz).


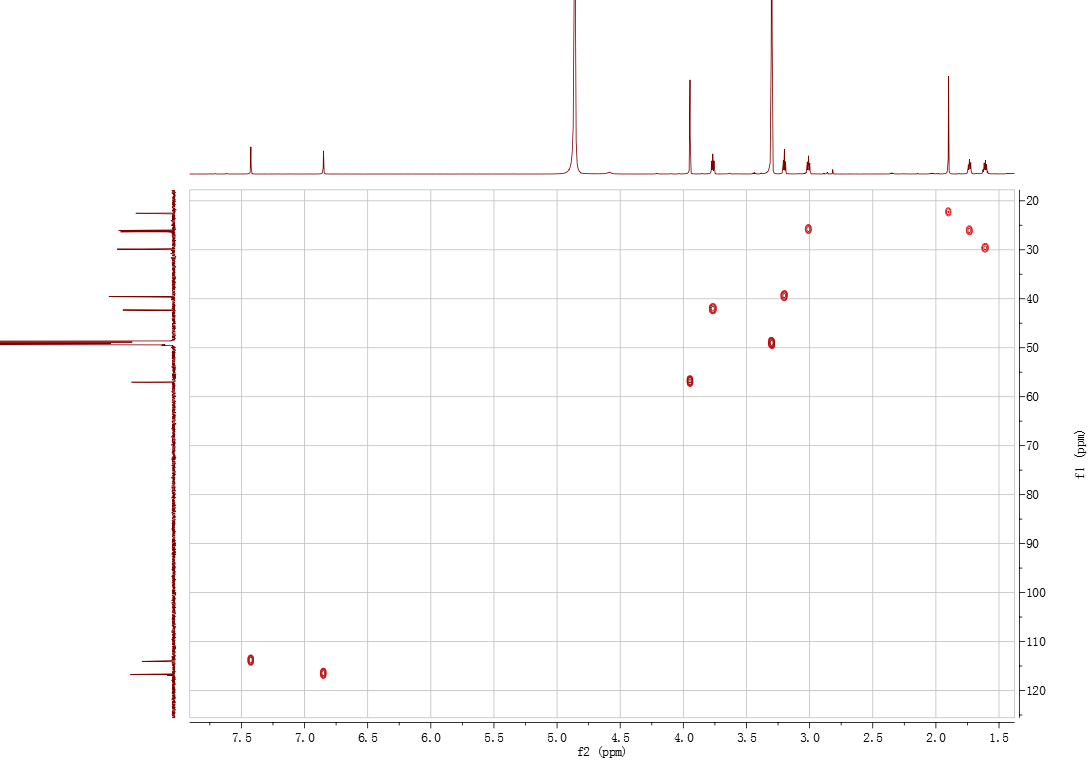


**Fig. S17.** HSQC spectrum of **2a**.


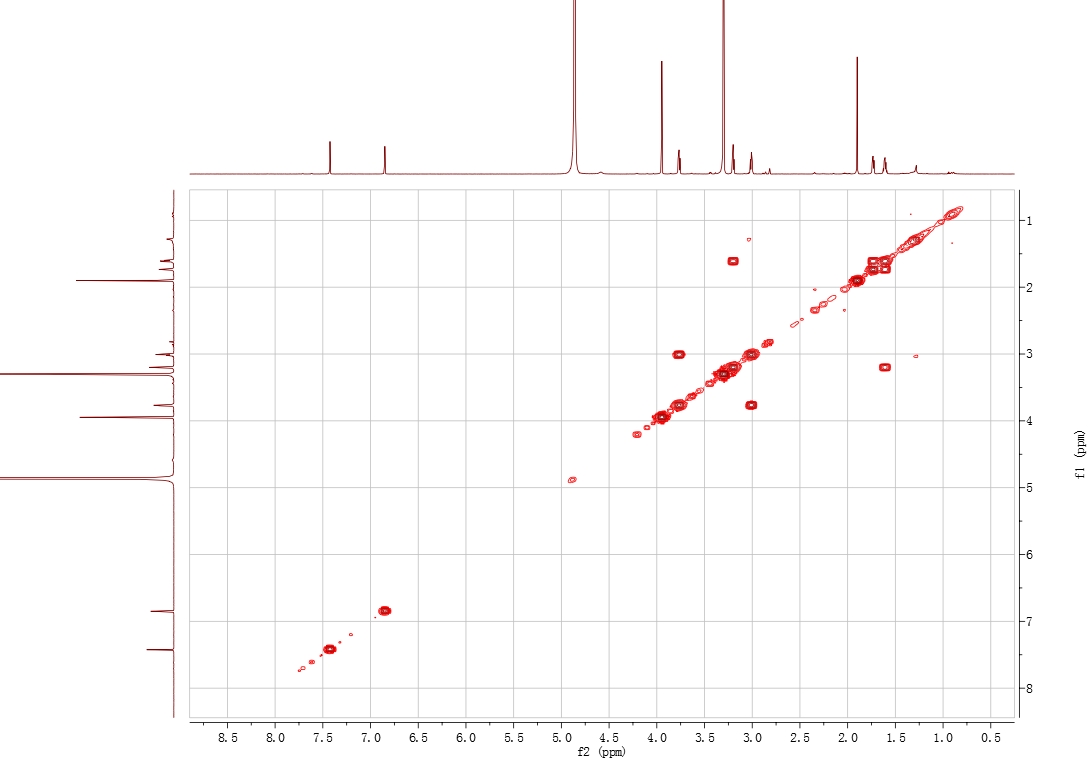


**Fig. S18.** ^1^H–^1^H COSY spectrum of **2a**.


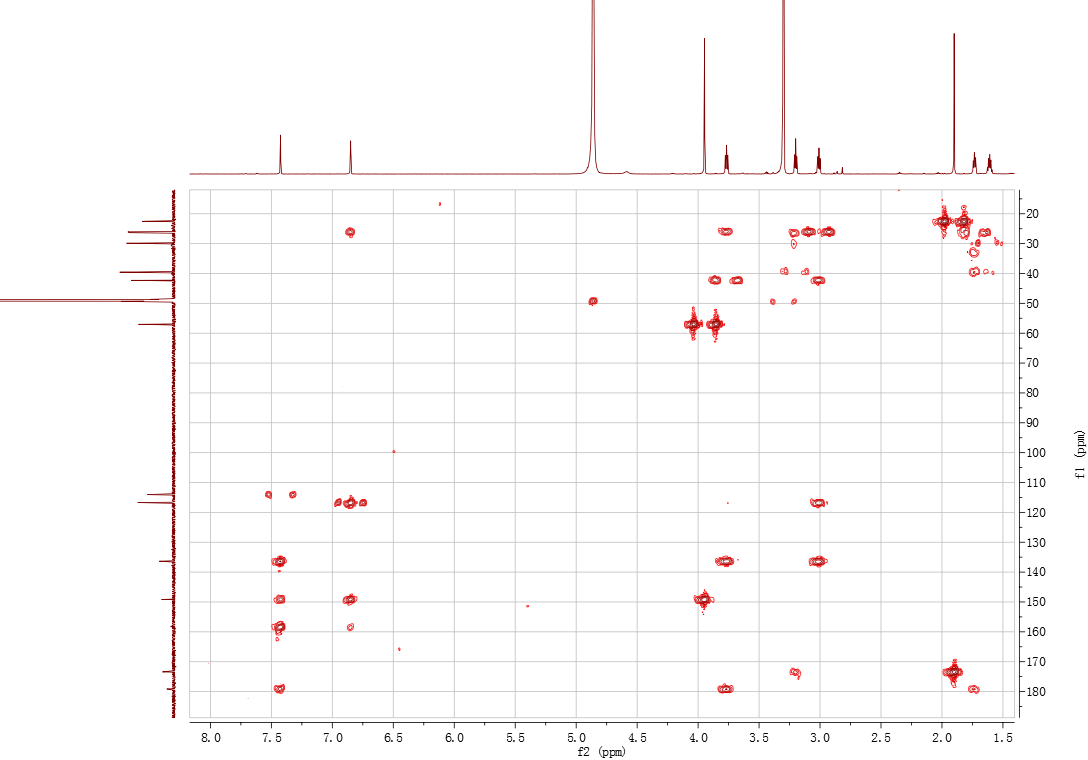


**Fig. S19.** HMBC spectrum of **2a**.


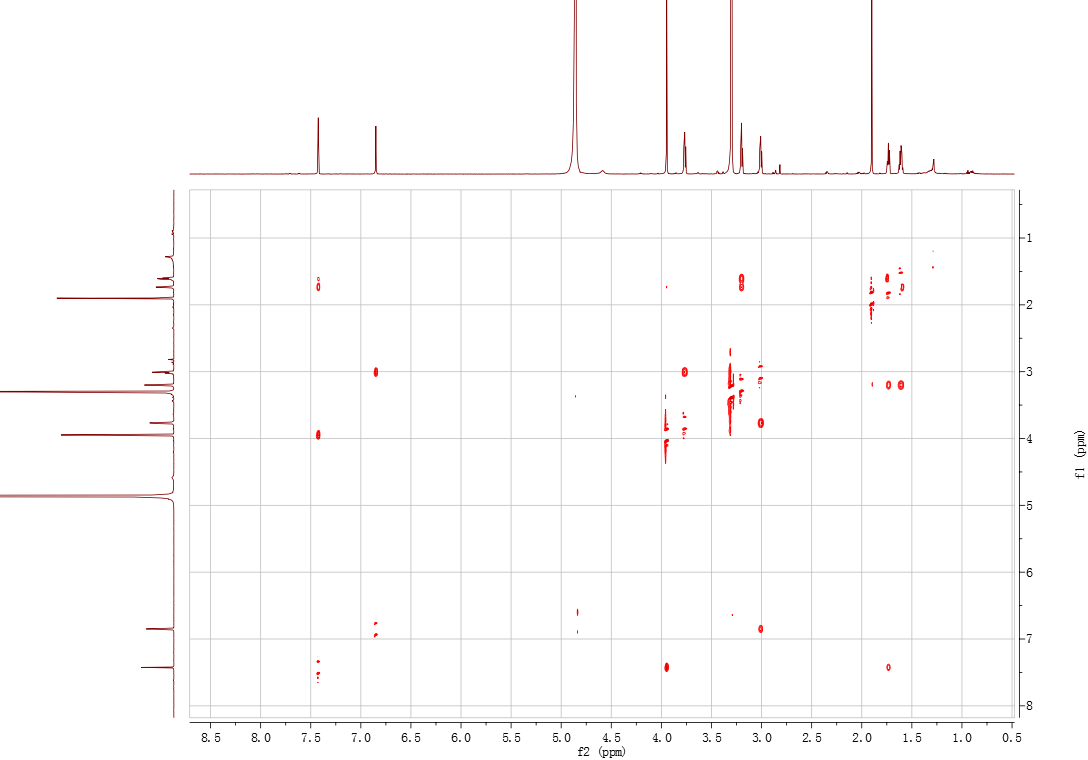


**Fig. S20.** ROESY spectrum of **2a**.


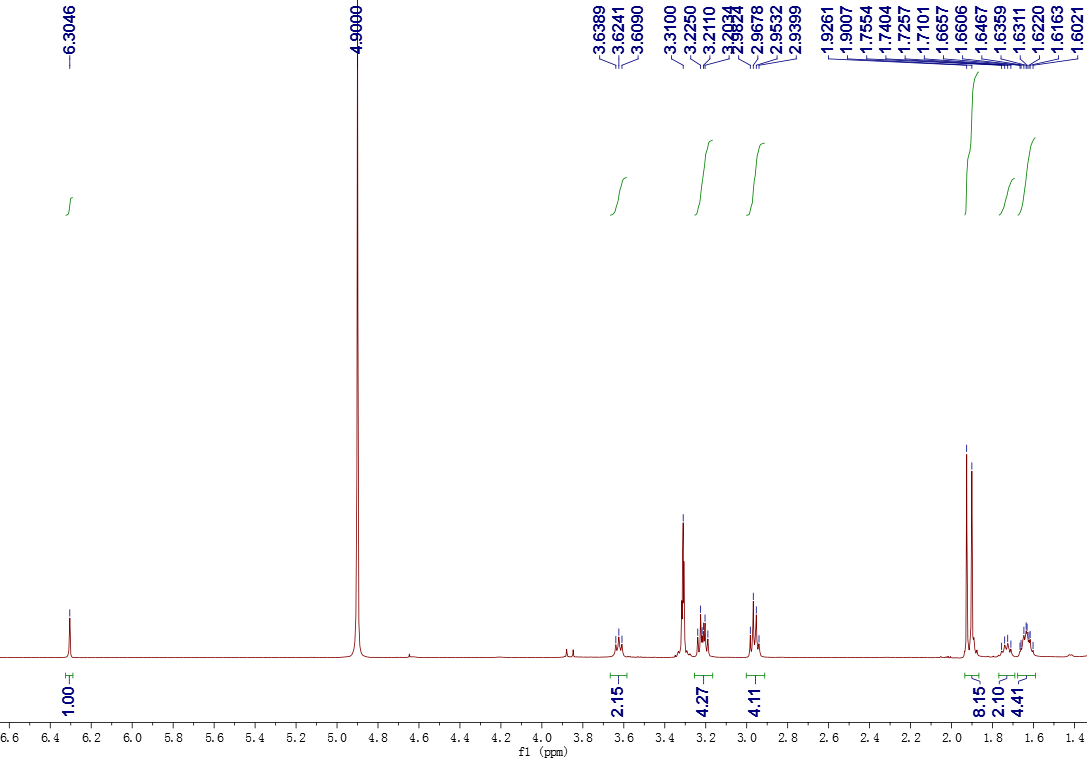


**Fig. S21.** ^1^H NMR spectrum of **3** (CD_3_OD, 500 MHz).


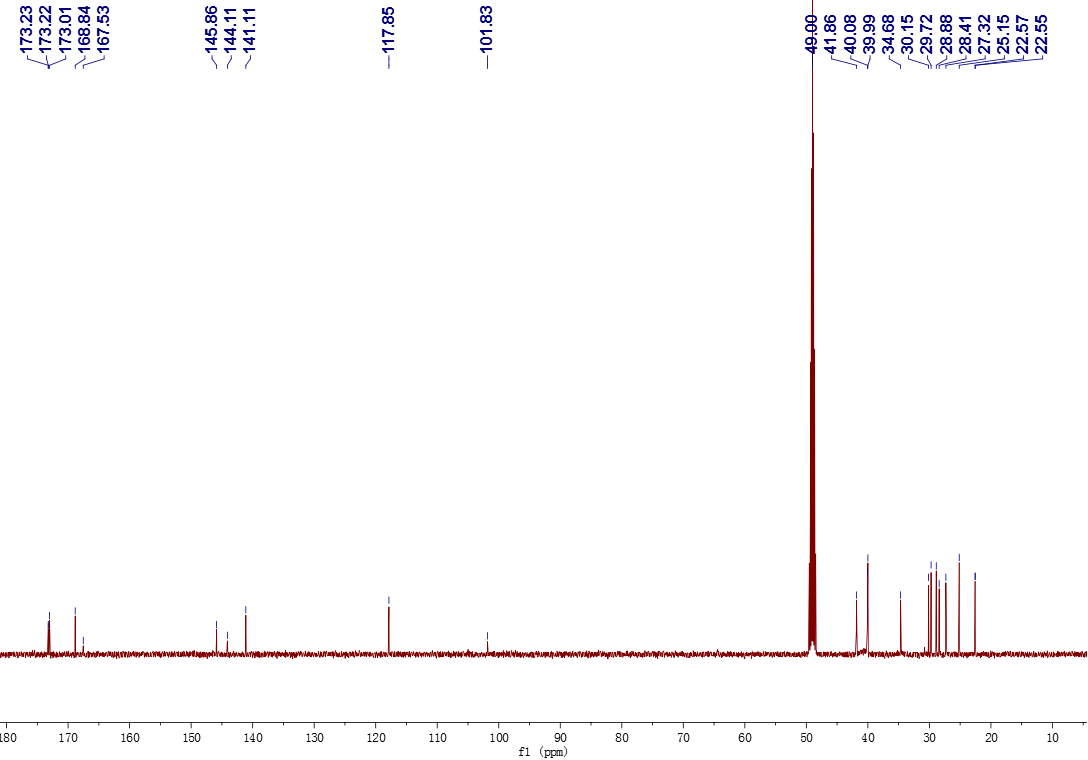


**Fig. S22.** ^13^C NMR spectrum of **3** (CD_3_OD, 120 MHz).


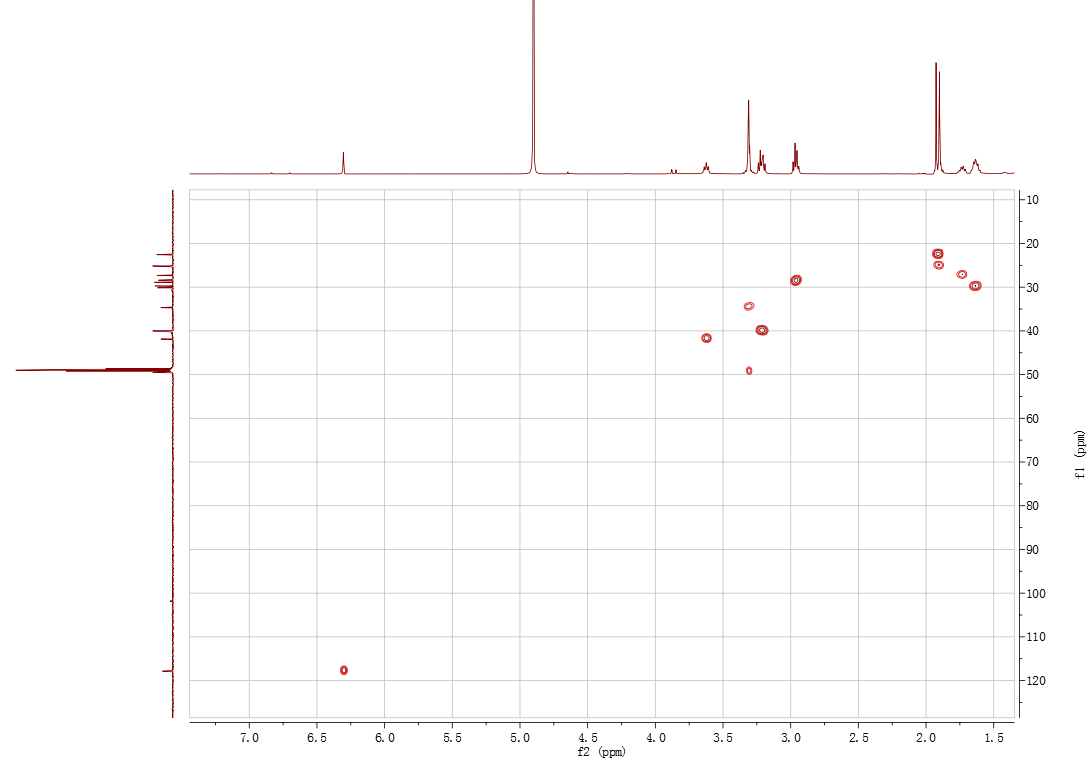


**Fig. S23.** HSQC spectrum of **3**.


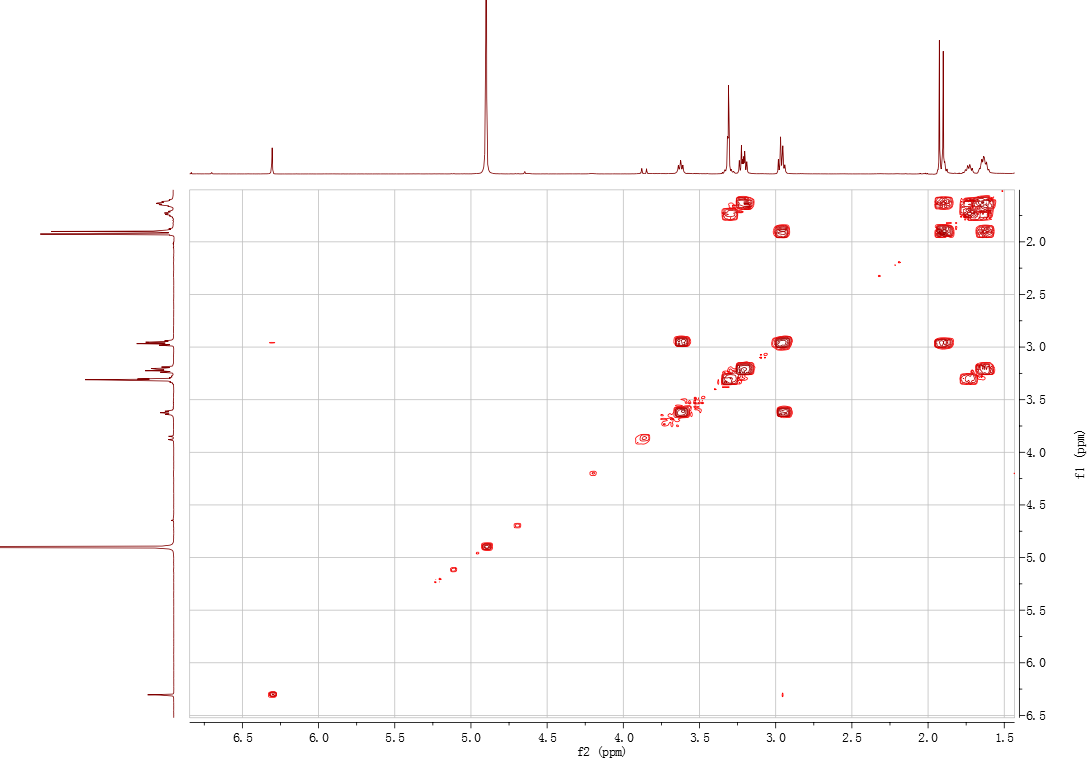


**Fig. S24.** ^1^H–^1^H COSY spectrum of **3**.


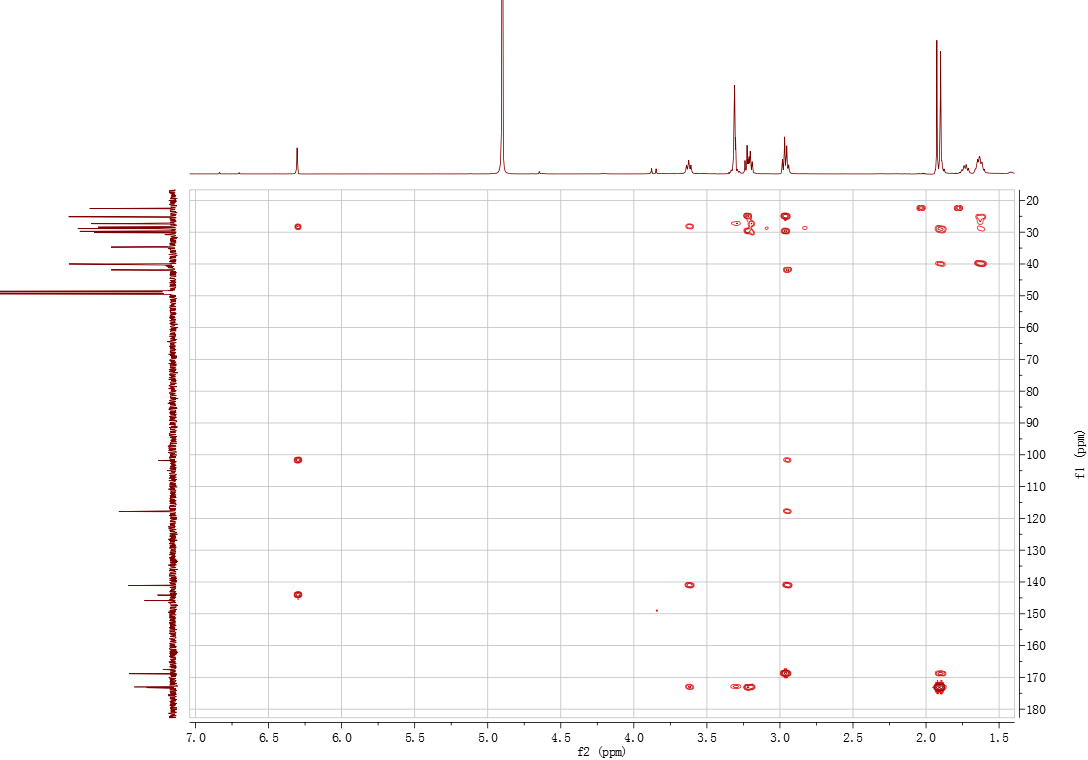


**Fig. S25.** HMBC spectrum of **3**.


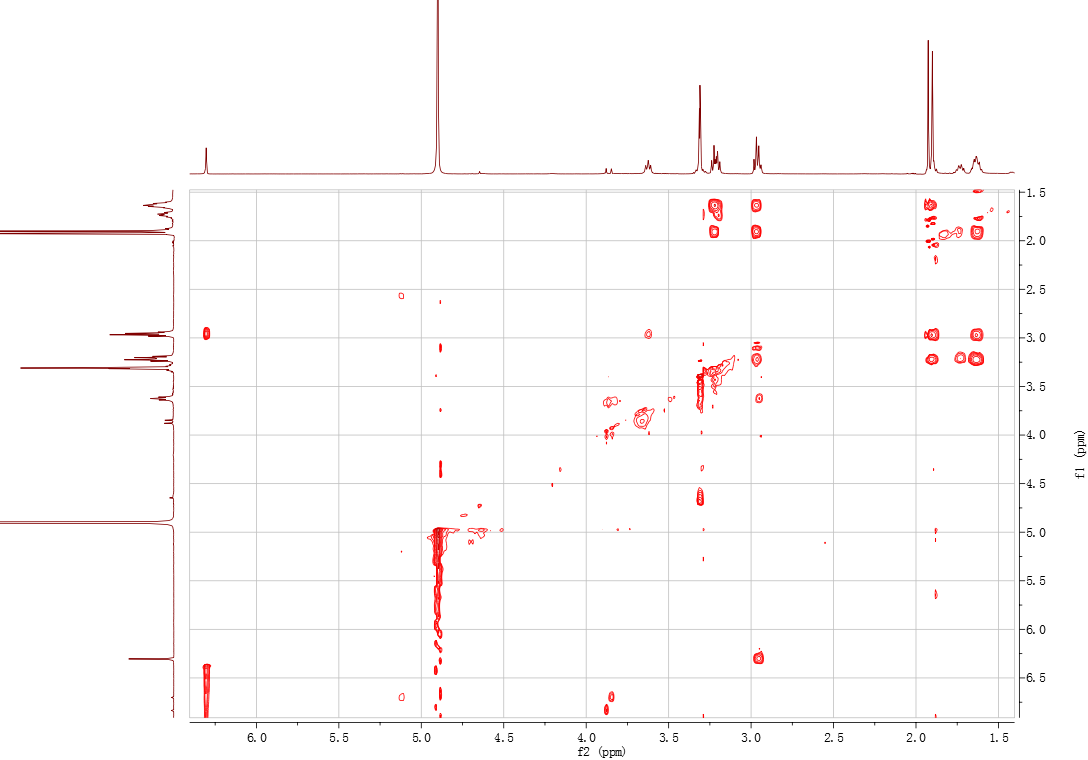


**Fig. S26.** ROESY spectrum of **3**.


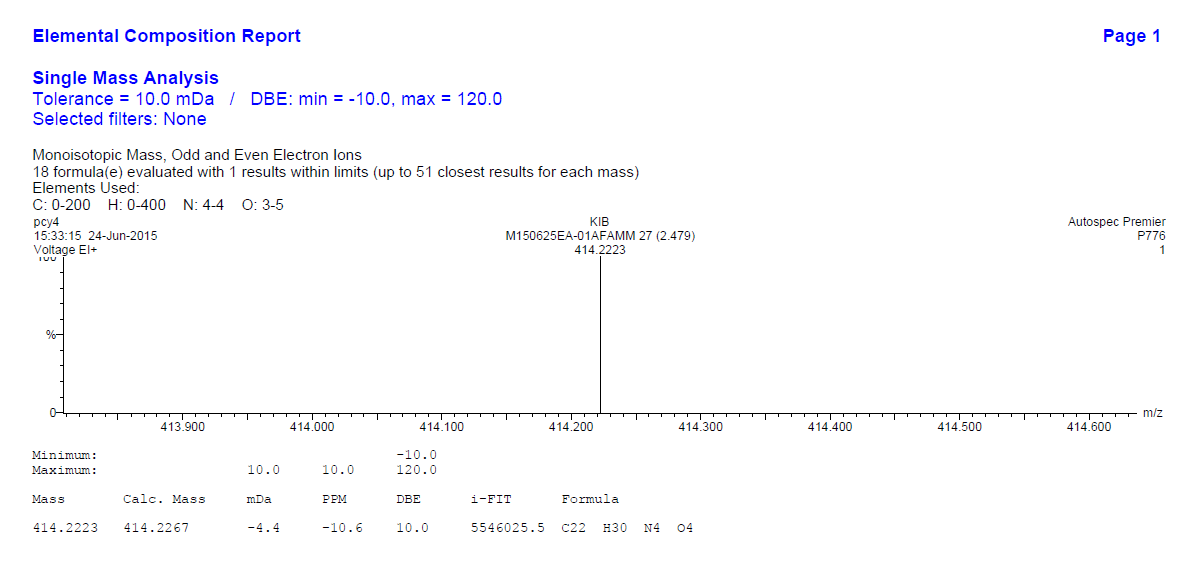


**Fig. S27.** HREIMS spectrum of **3**.


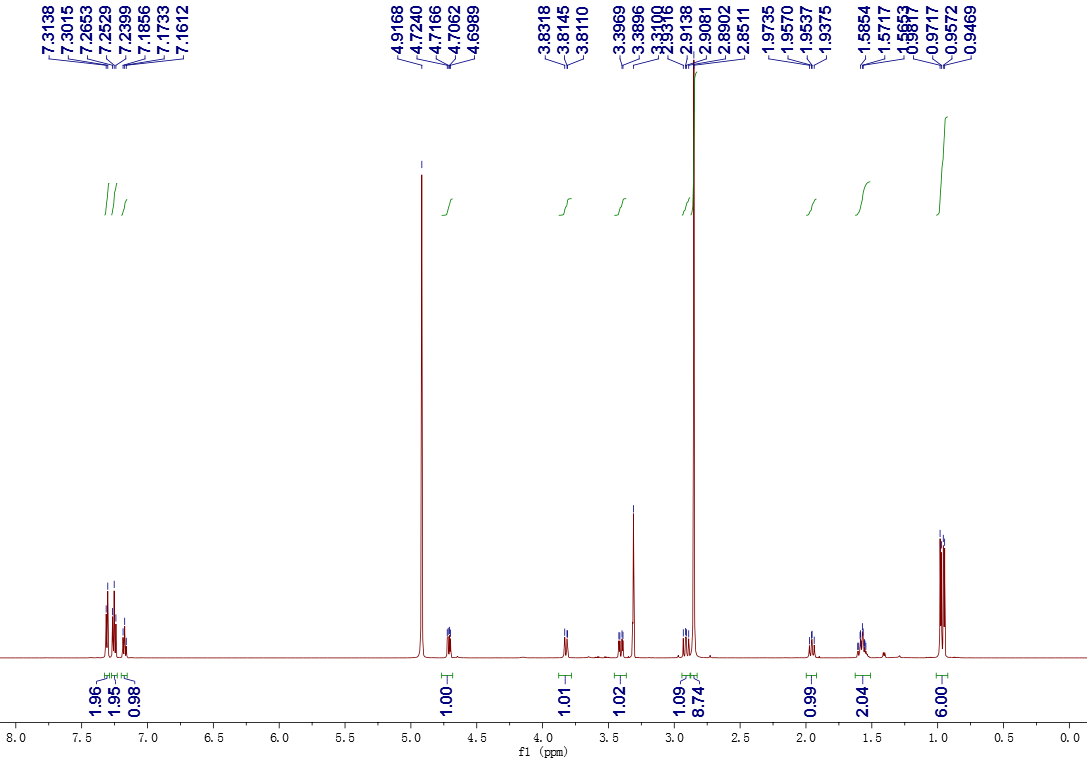


**Fig. S28.** ^1^H NMR spectrum of **4** (CD_3_OD, 600 MHz).


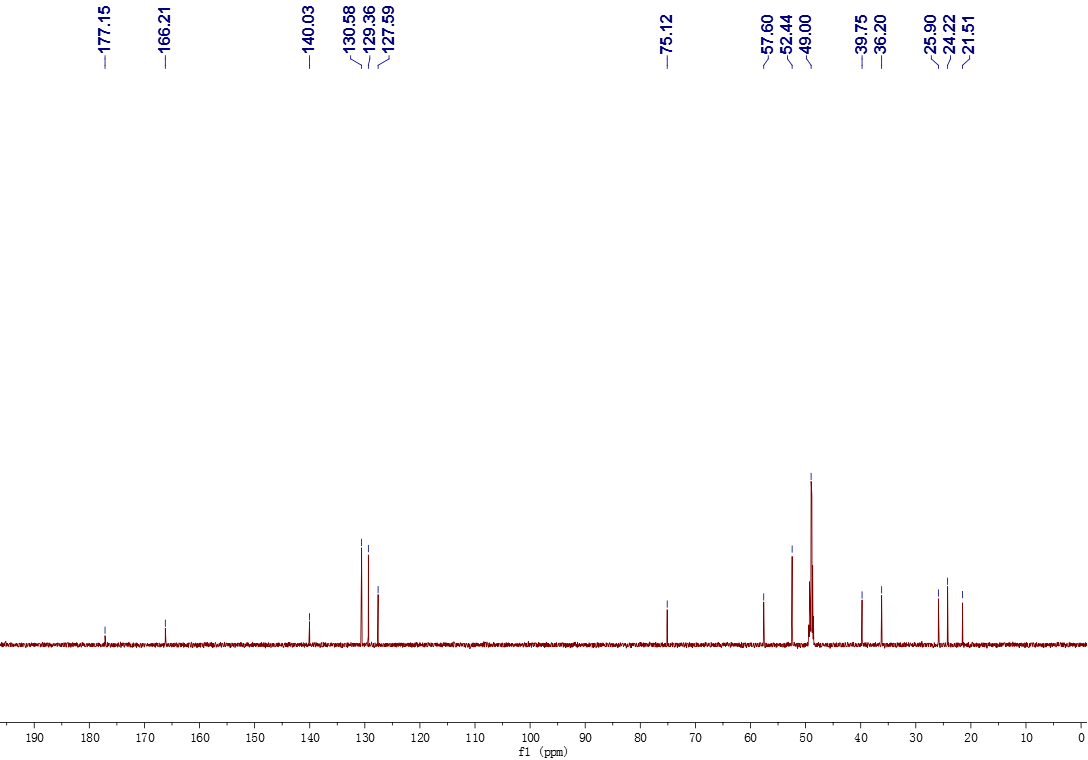


**Fig. S29.** ^13^C NMR spectrum of **4** (CD_3_OD, 150 MHz).


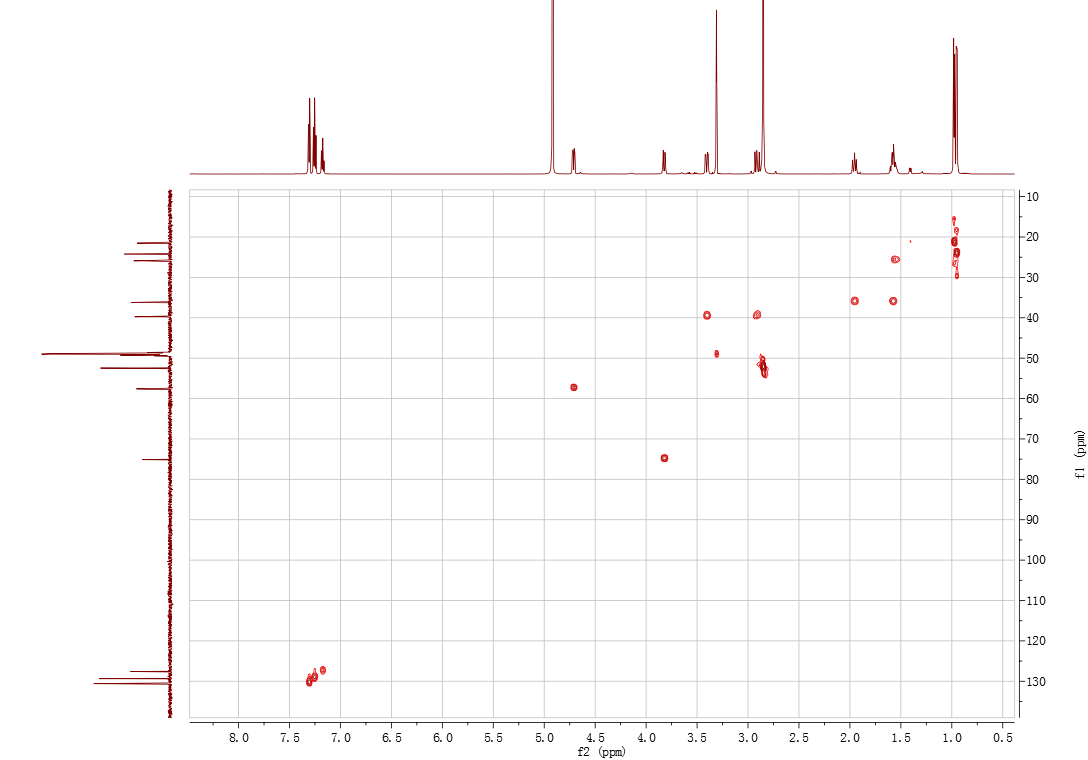


**Fig. S30.** HSQC spectrum of **4**.


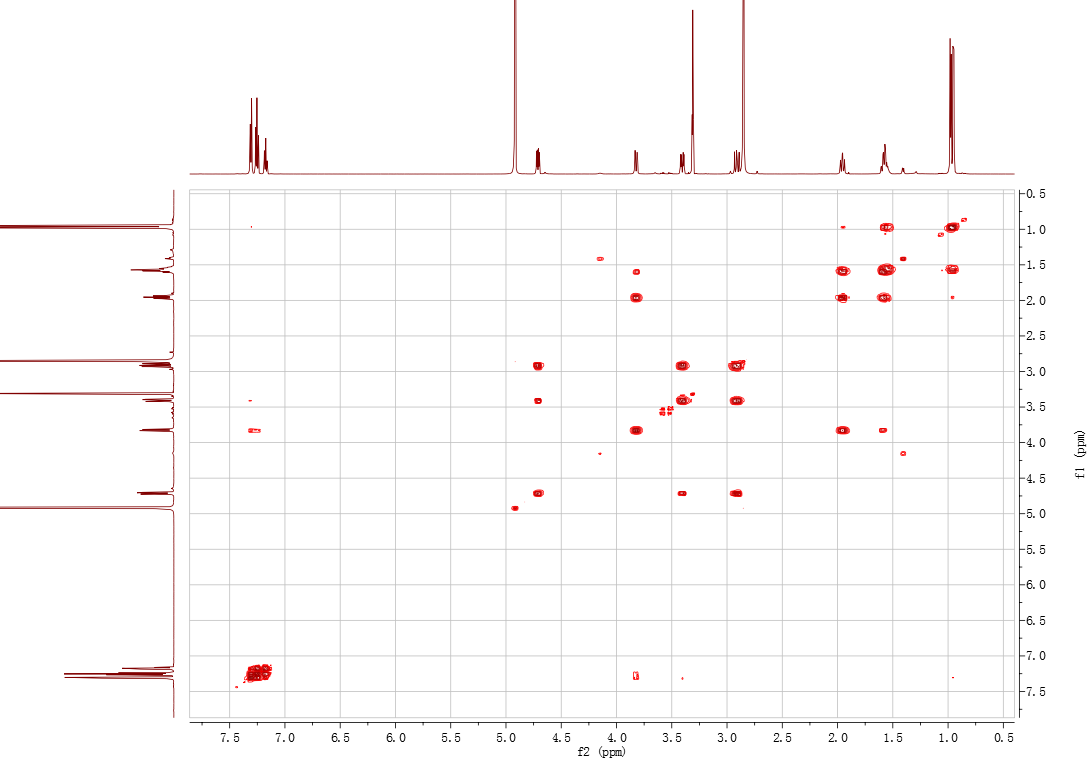


**Fig. S31.** ^1^H–^1^H COSY spectrum of **4**.


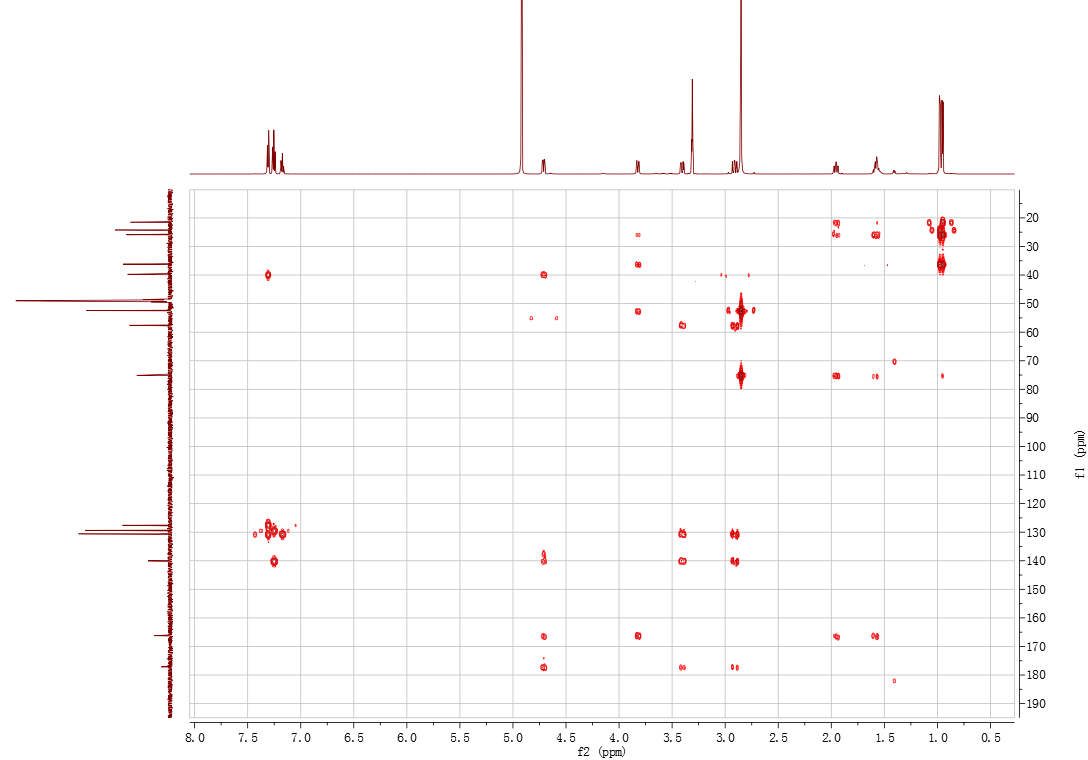


**Fig. S32.** HMBC spectrum of **4**.


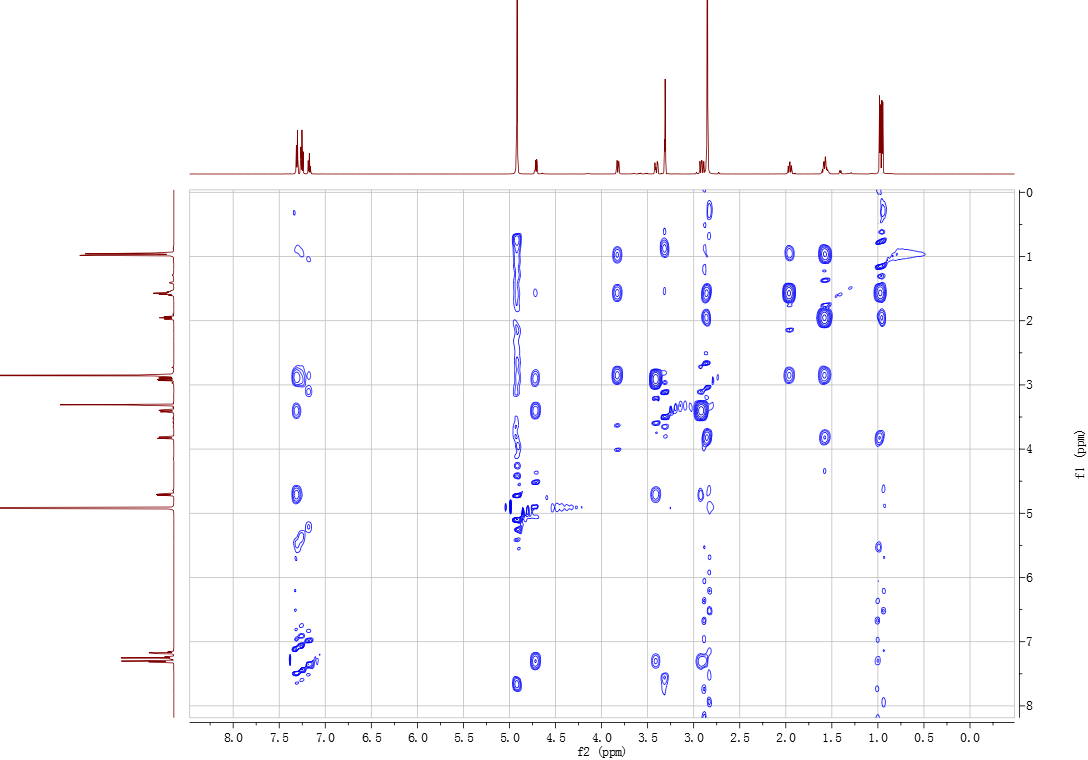


**Fig. S33.** ROESY spectrum of **4**.


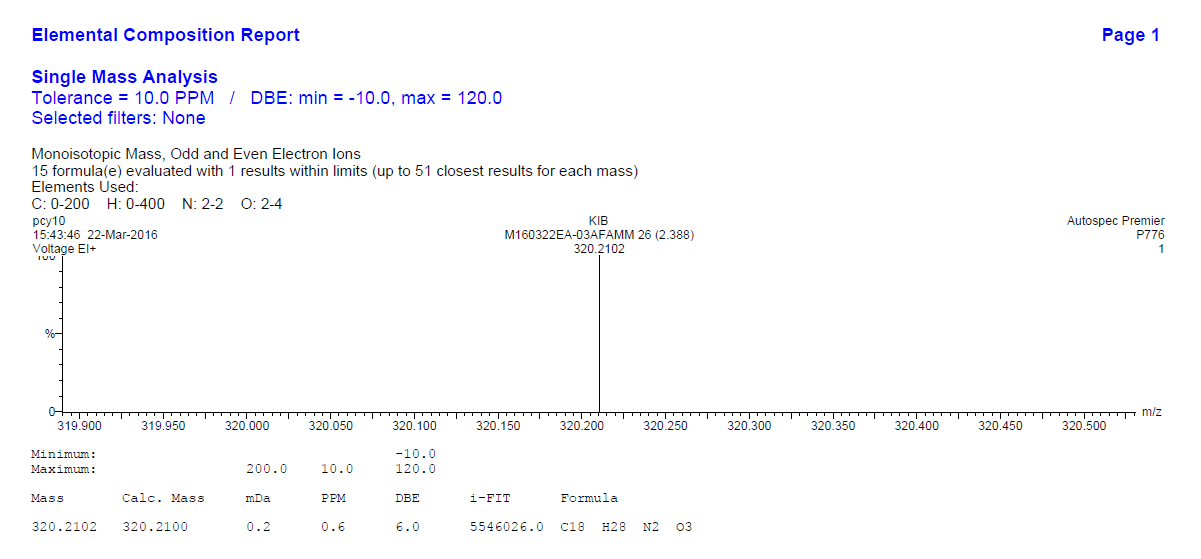


**Fig. S34.** HREIMS spectrum of **4**.


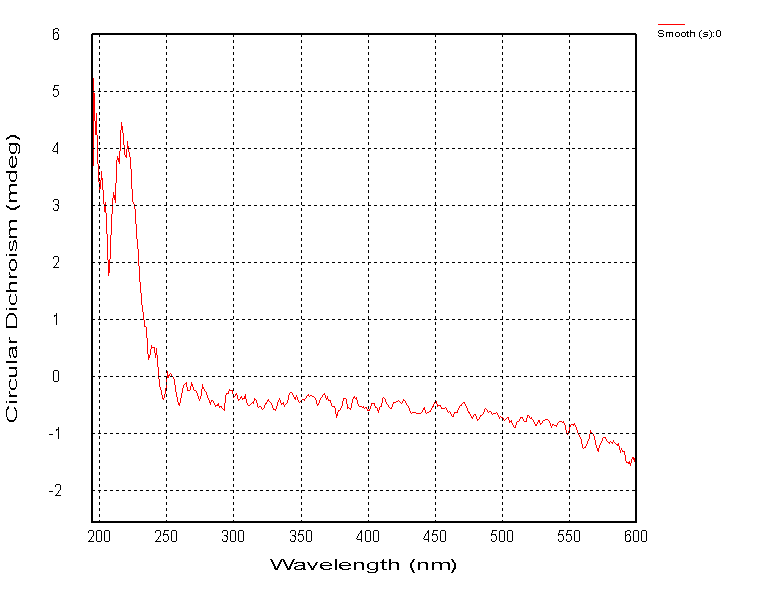


File: CD PCY10-1mm(195-600)16031704.dsx

ProBinaryX

Attributes :

- Time Stamp :Thu Mar 17 10:18:56 2016

- File ID : {02CAA986-265E-4343-AEC1-23AFBA9FA6C3}

- Is CFR Compliant : false

- Original unaltered data

Remarks:

- HV (CDDC channel): 0 v

- Time per point: 1 s

- Description: Sample 1

- Concentration: 0.8000mg/mL MeOH

- Pathlength: 1 mm

Settings:

- Time-per-point: 1s (25us x 40000)

- Wavelength: 195nm - 600nm

- Step Size: 1nm

- Bandwidth: 1nm

**Fig. S35.** ECD spectrum of **4**.


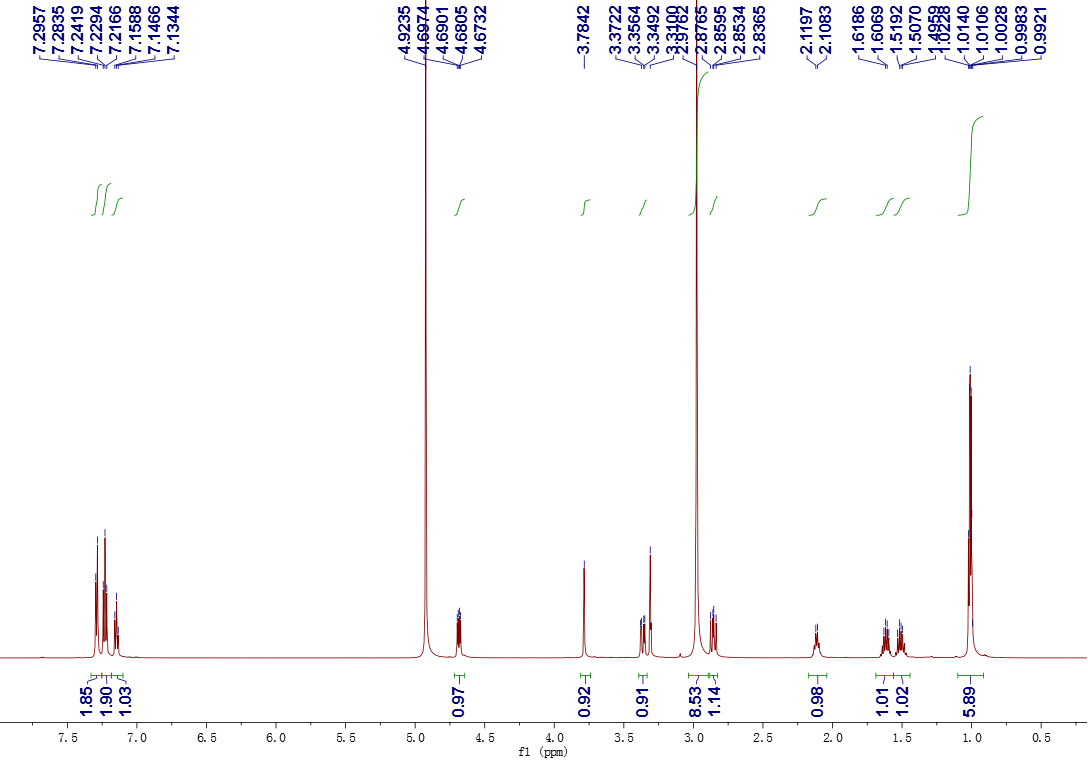


**Fig. S36.** ^1^H NMR spectrum of **5** (CD_3_OD, 600 MHz).


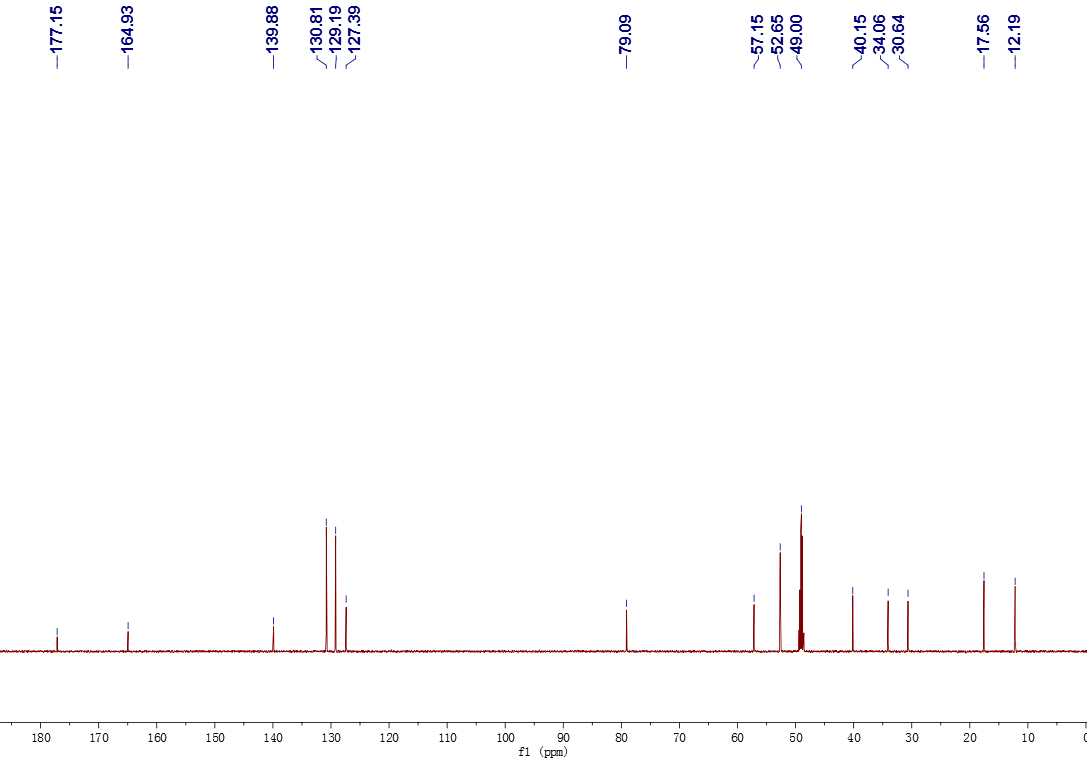


**Fig. S37.** ^1^H NMR spectrum of **5** (CD_3_OD, 150 MHz).


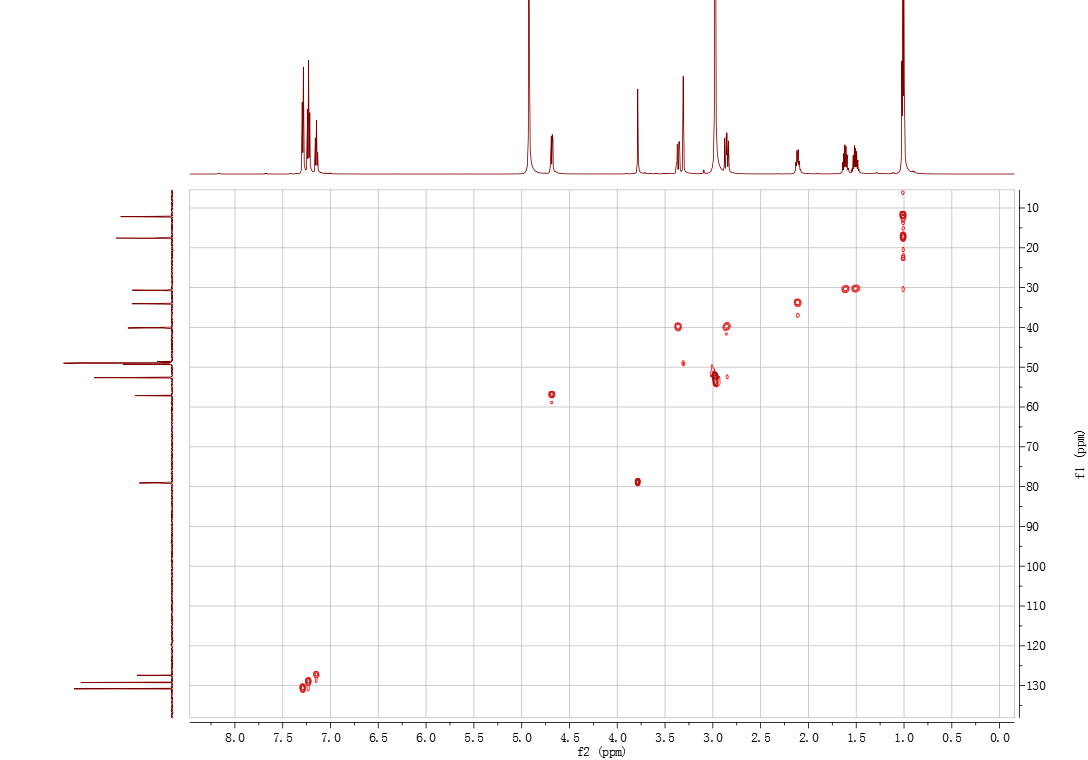


**Fig. S38.** HSQC spectrum of **5**.


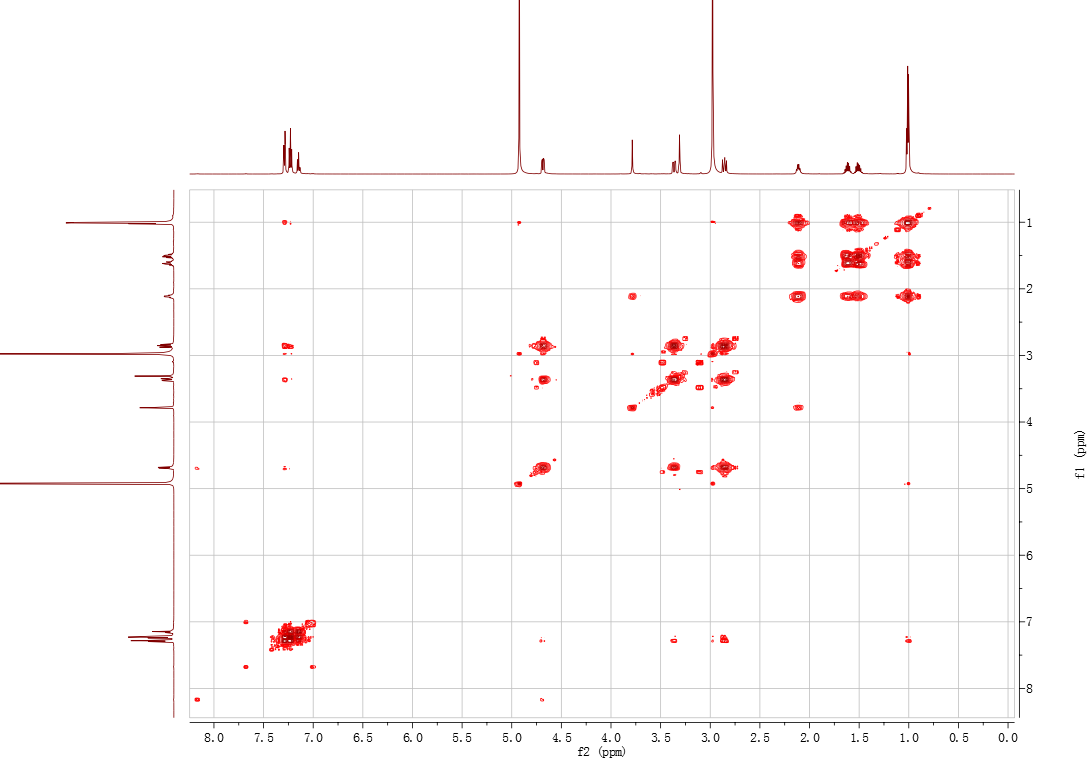


**Fig. S39.** ^1^H–^1^H COSY spectrum of **5**.


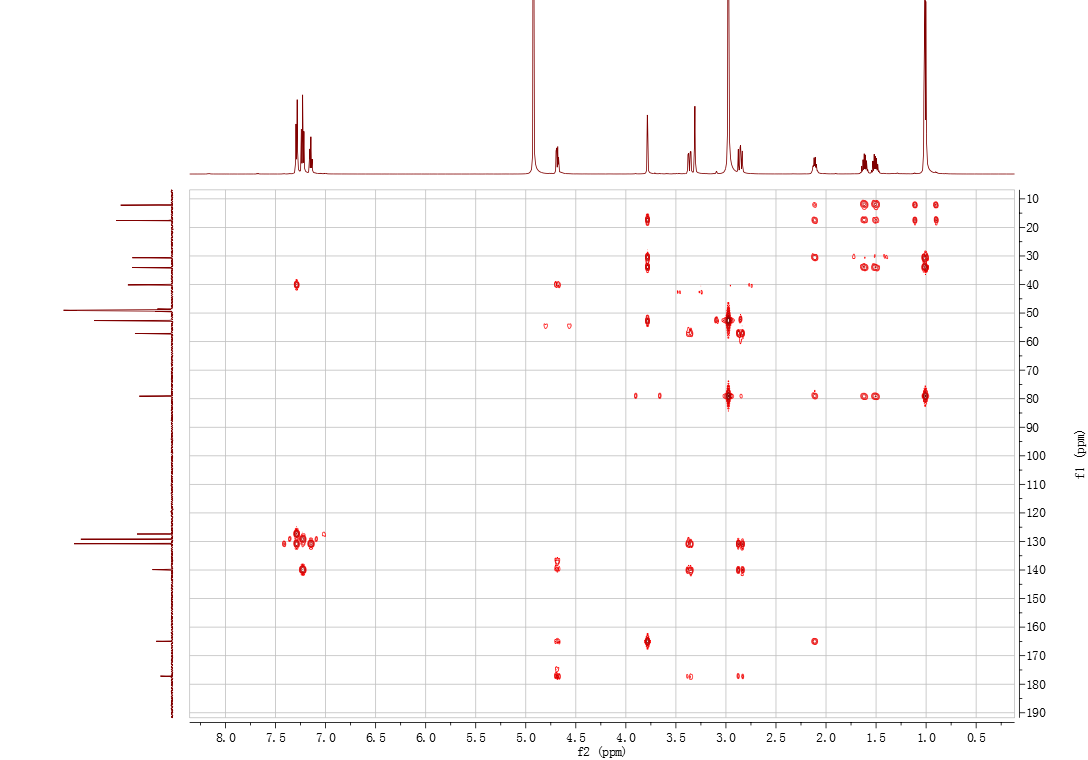


**Fig. S40.** HMBC spectrum of **5**.

**Fig. S41.** ROESY spectrum of **5**.


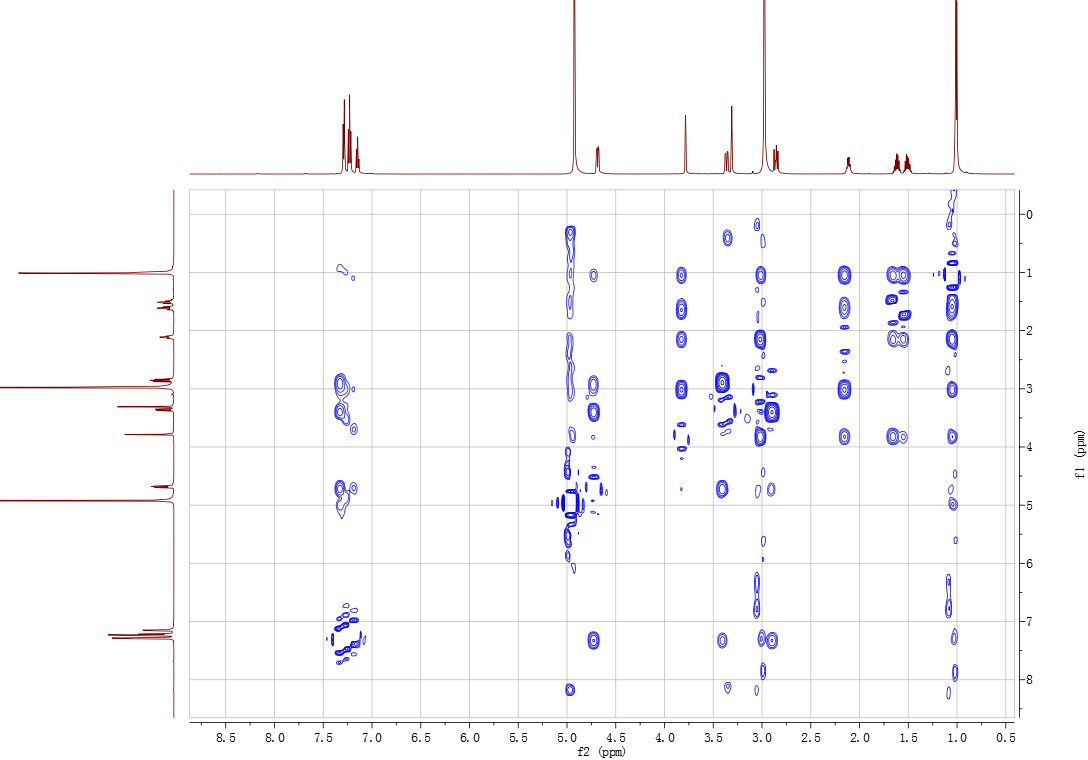


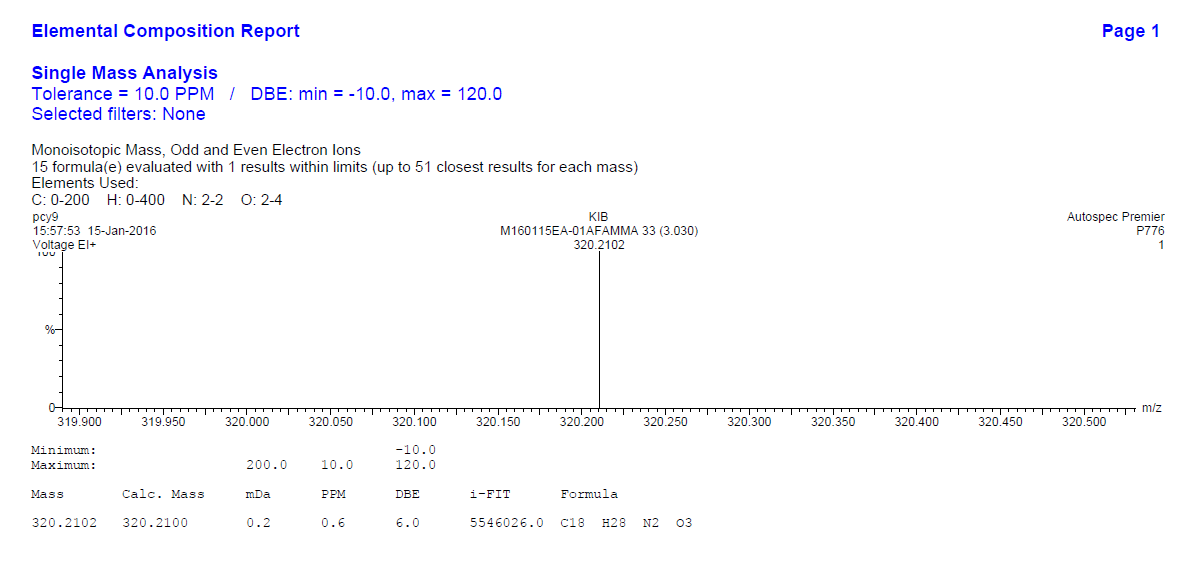


**Fig. S42.** HREIMS spectrum of **5**.


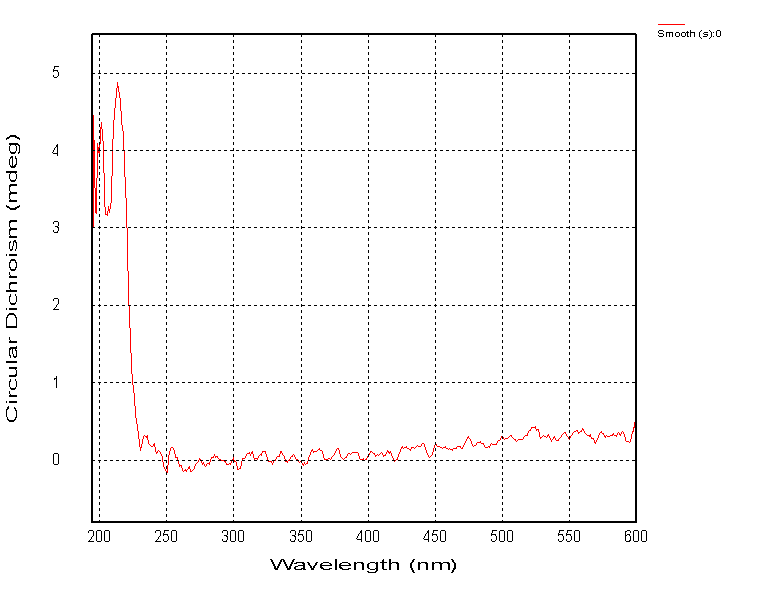


File: CD PCY9-1mm(195-600)16012419.dsx

ProBinaryX

Attributes :

- Time Stamp :Sun Jan 24 19:35:01 2016

- File ID : {1955187F-4840-42d6-86B9-9F7798A7835B}

- Is CFR Compliant : false

- Original unaltered data

Remarks:

- HV (CDDC channel): 0 v

- Time per point: 1 s

- Description: Sample 1

- Concentration: 0.1224mg/mL MeOH

- Pathlength: 1 mm

Settings:

- Time-per-point: 1s (25us x 40000)

- Wavelength: 195nm - 600nm

- Step Size: 1nm

- Bandwidth: 2nm

**Fig. S43.** ECD spectrum of **5**.


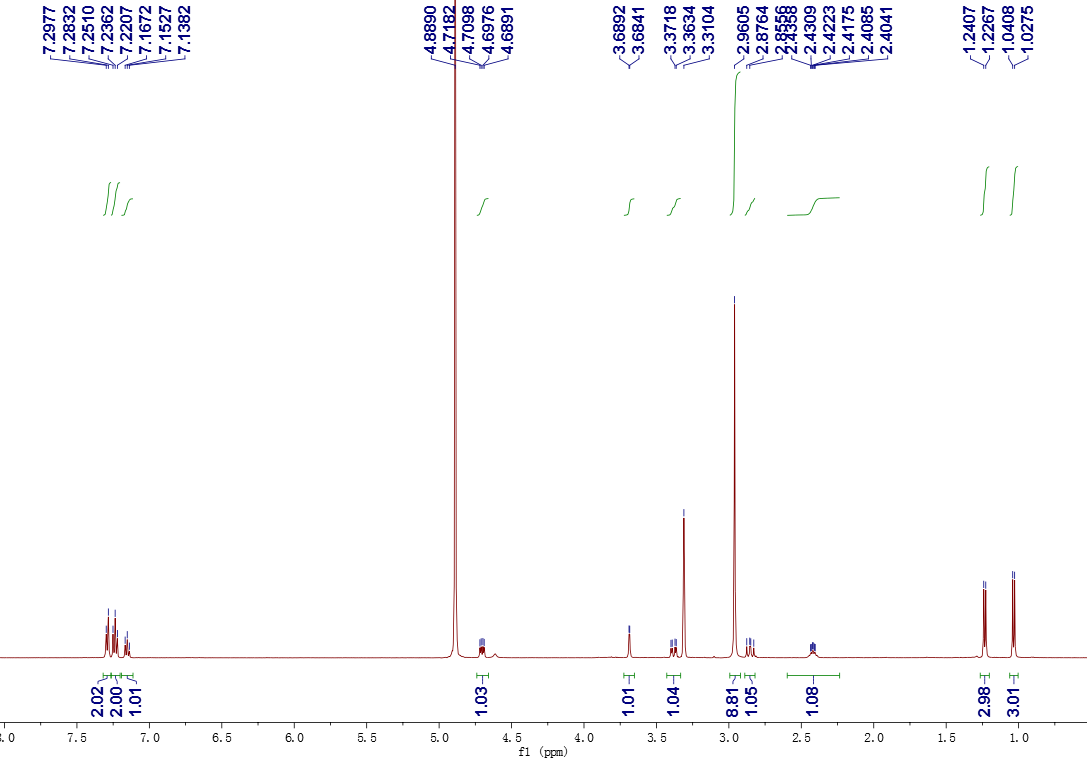


**Fig. S44.** ^1^H NMR spectrum of **6** (CD_3_OD, 400 MHz).


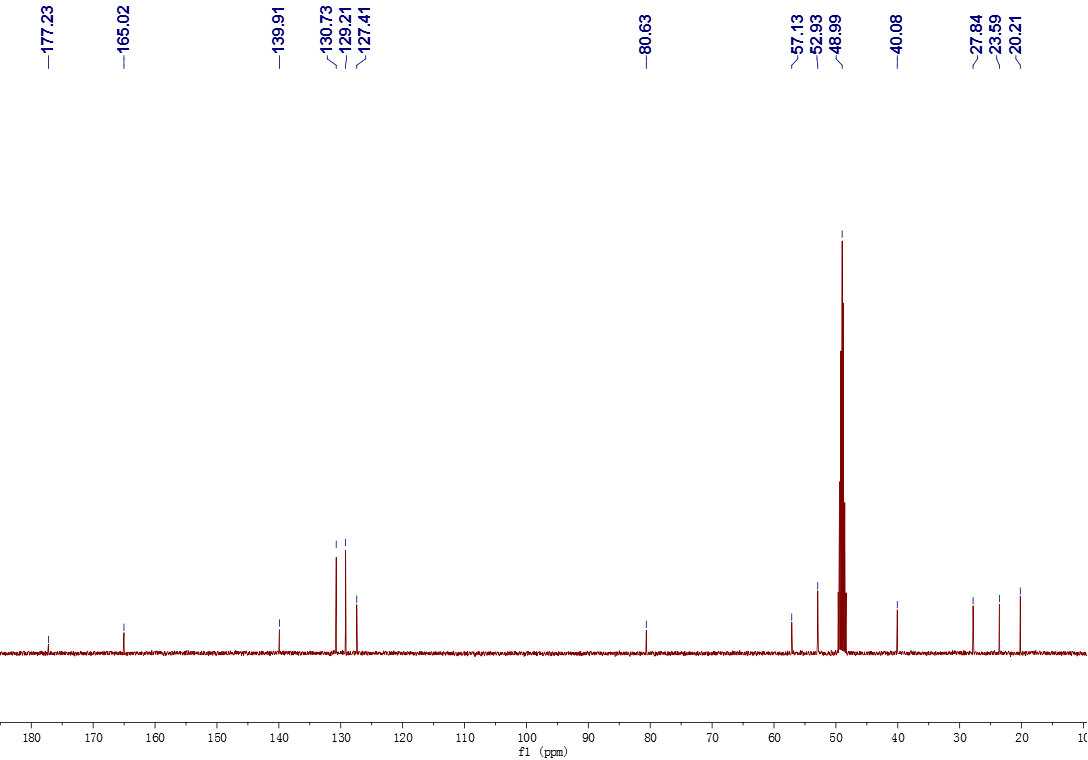


**Fig. S45.** ^1^H NMR spectrum of **6** (CD_3_OD, 100 MHz).


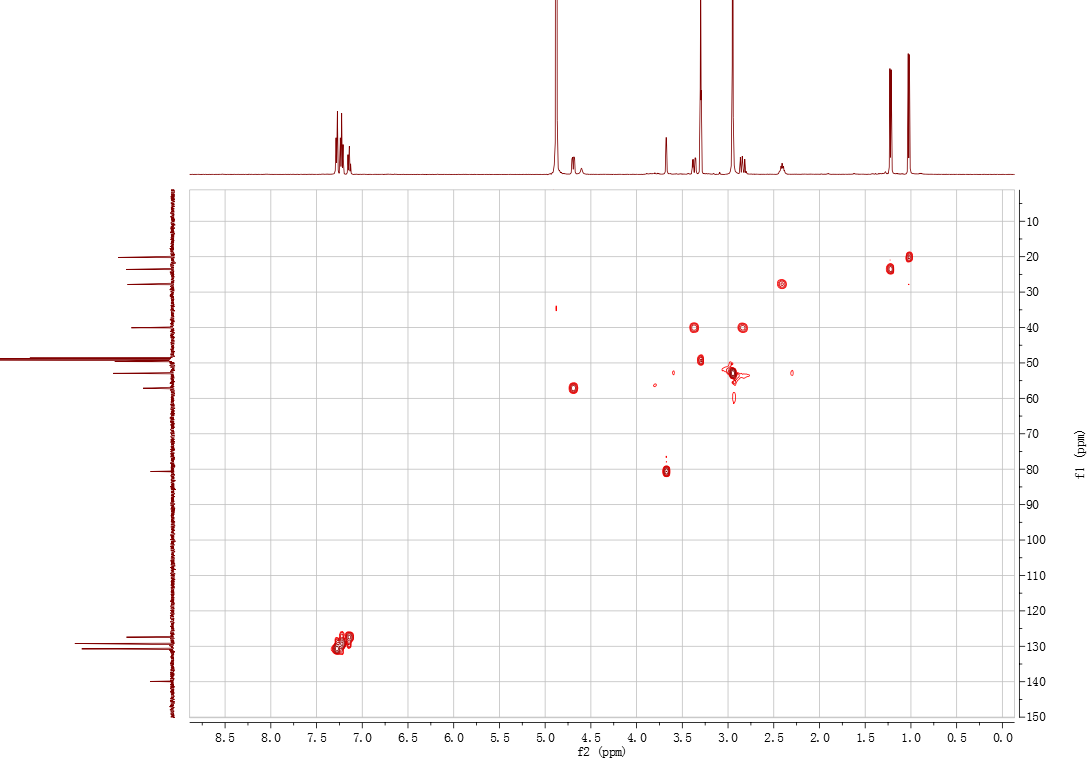


**Fig. S46.** HSQC spectrum of **6**.


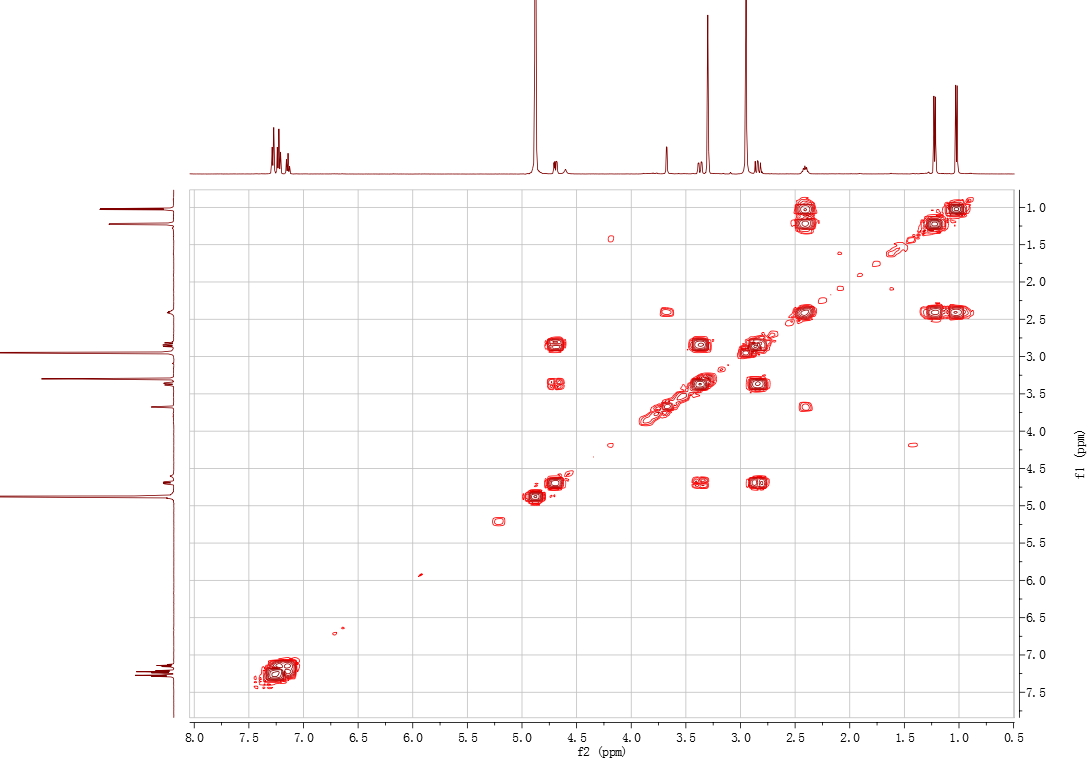


**Fig. S47.** ^1^H–^1^H COSY spectrum of **6**.


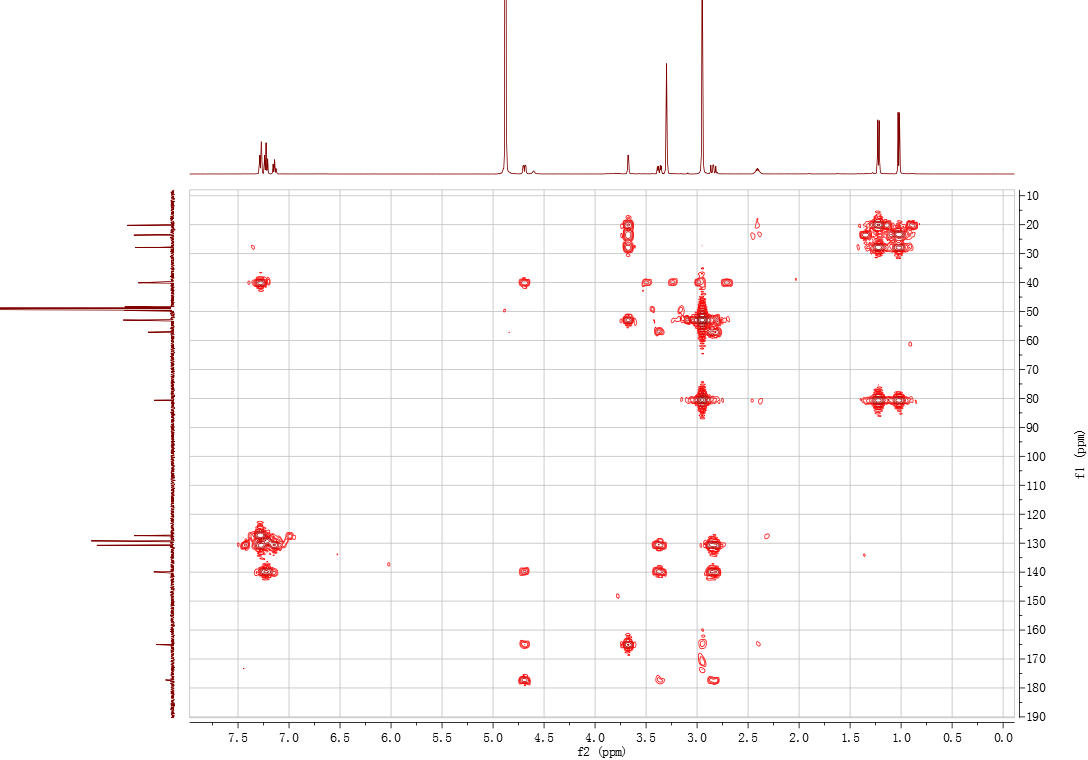


**Fig. S48.** HMBC spectrum of **6**.


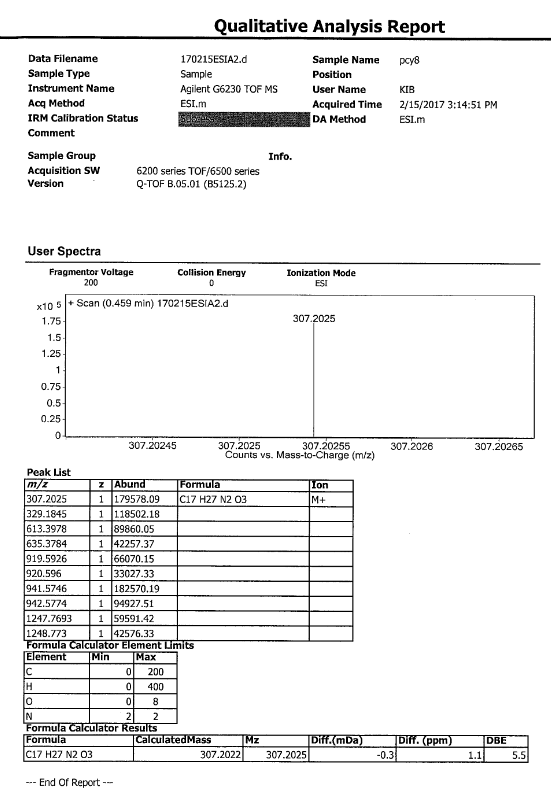


**Fig. S49.** HRESIMS spectrum of **6**.


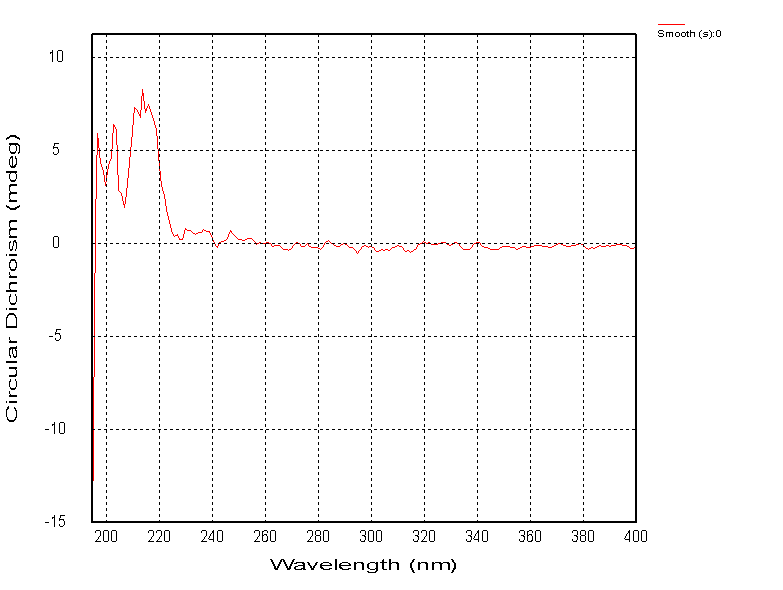


File: CD PCY8-1mm(195-400)17012503.dsx

ProBinaryX

Attributes :

- Time Stamp :Wed Jan 25 09:46:08 2017

- File ID : {F5220F38-696B-40bf-BFCB-F0092BB5B72B}

- Is CFR Compliant : false

- Original unaltered data

Remarks:

- HV (CDDC channel): 0 v

- Time per point: 1 s

- Description: Sample 1

- Concentration: 0.1350mg/mL MeOH

- Pathlength: 1 mm

Settings:

- Time-per-point: 1s (25us x 40000)

- Wavelength: 195nm - 400nm

- Step Size: 1nm

- Bandwidth: 1nm

**Fig. S50.** ECD spectrum of **6**.


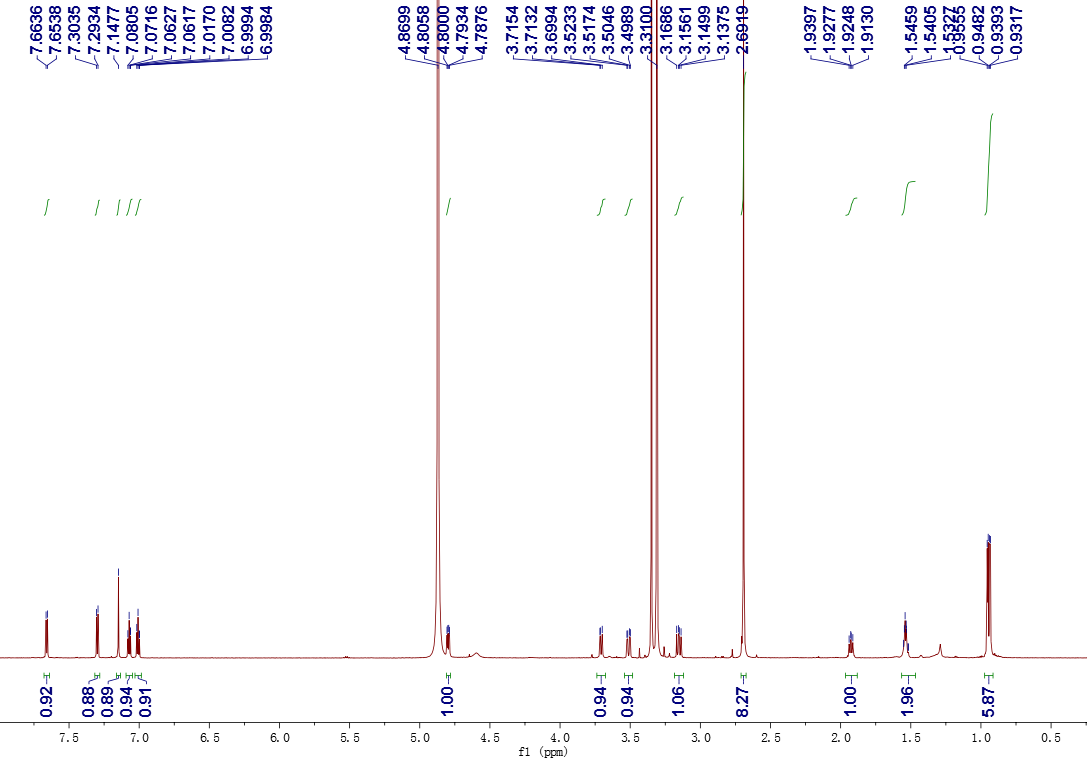


**Fig. S51.** ^1^H NMR spectrum of **7** (CD_3_OD, 800 MHz).


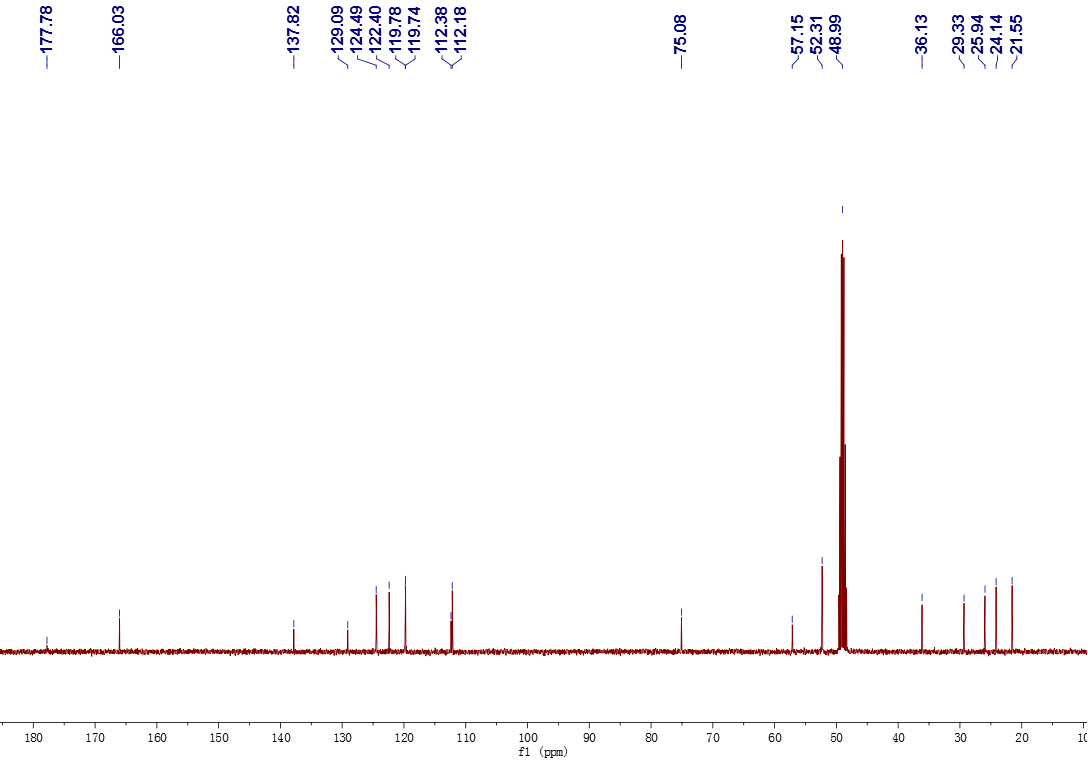


**Fig. S52.** ^13^C NMR spectrum of **7** (CD_3_OD, 100 MHz).


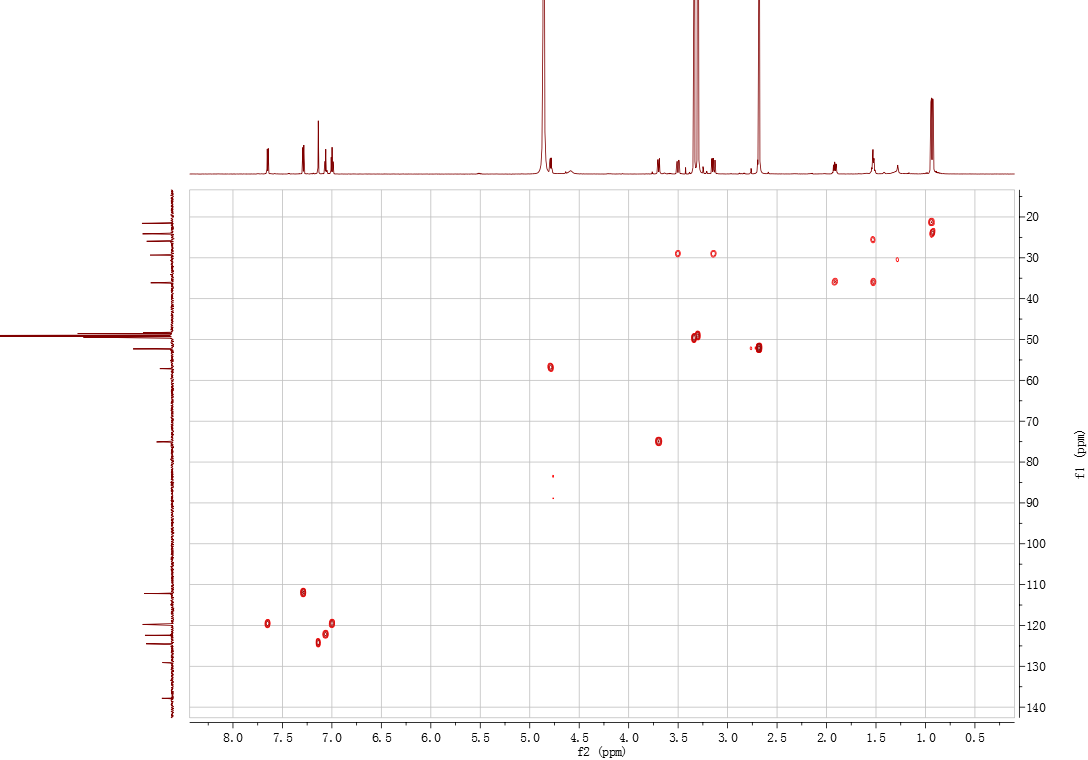


**Fig. S53.** HSQC spectrum of **7**.


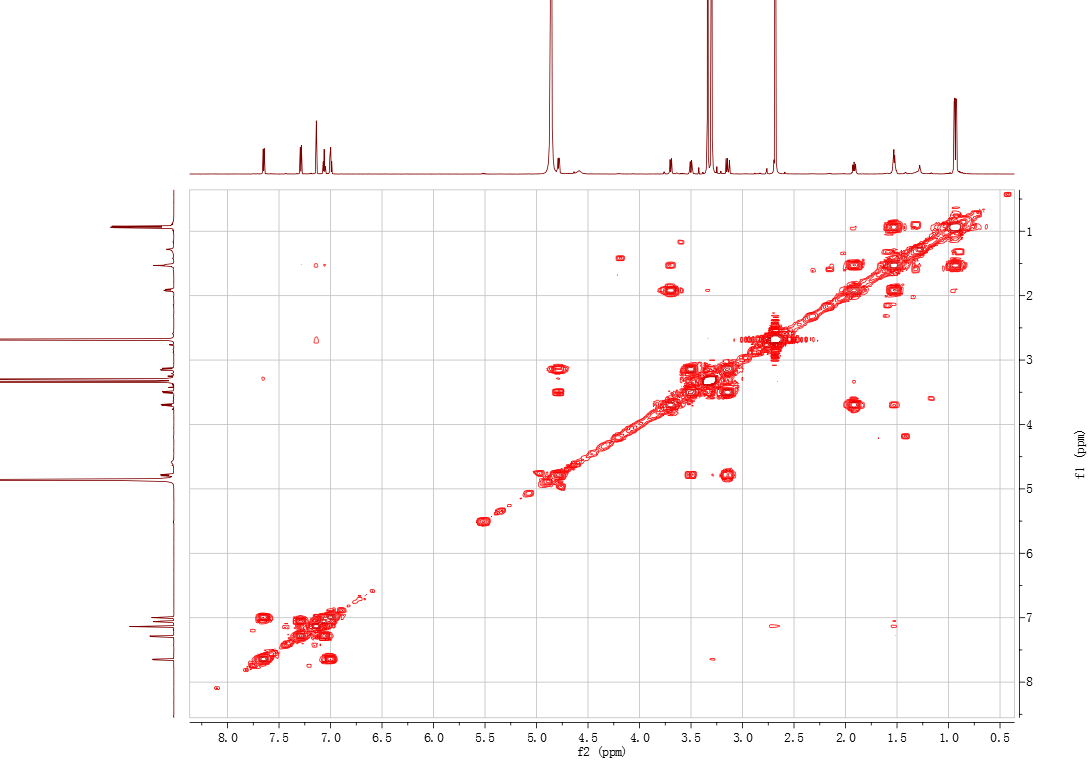


**Fig. S54.** ^1^H–^1^H COSY spectrum of **7**.


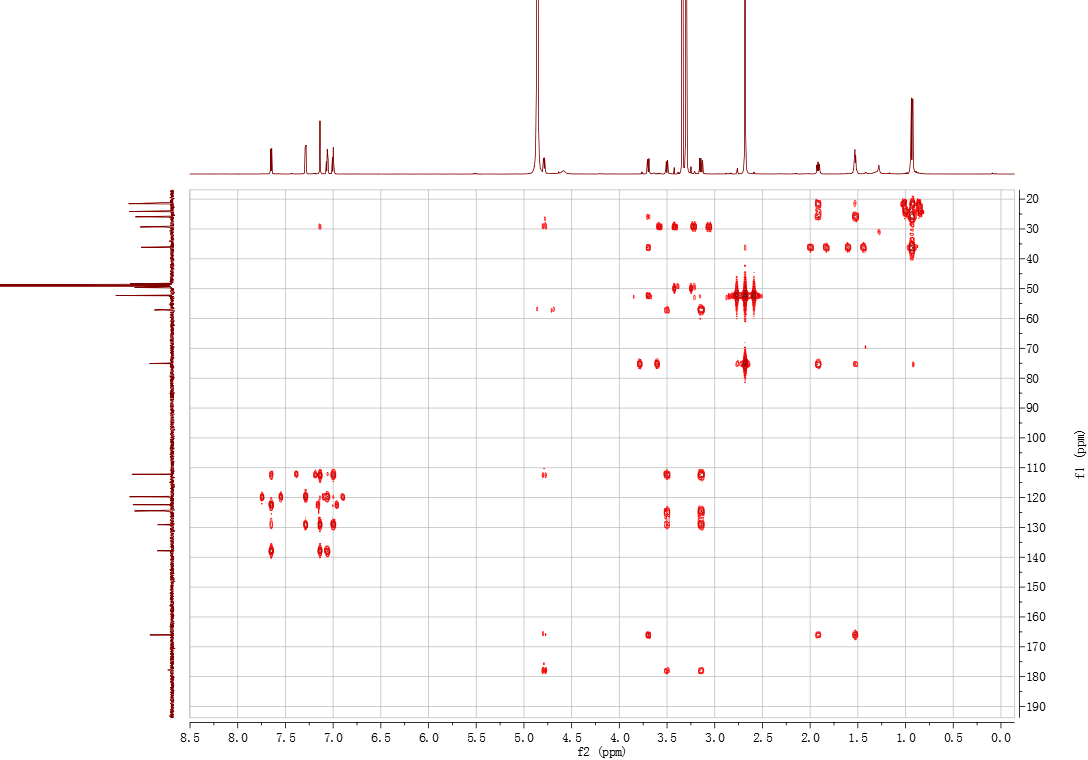


**Fig. S55.** HMBC spectrum of **7**.


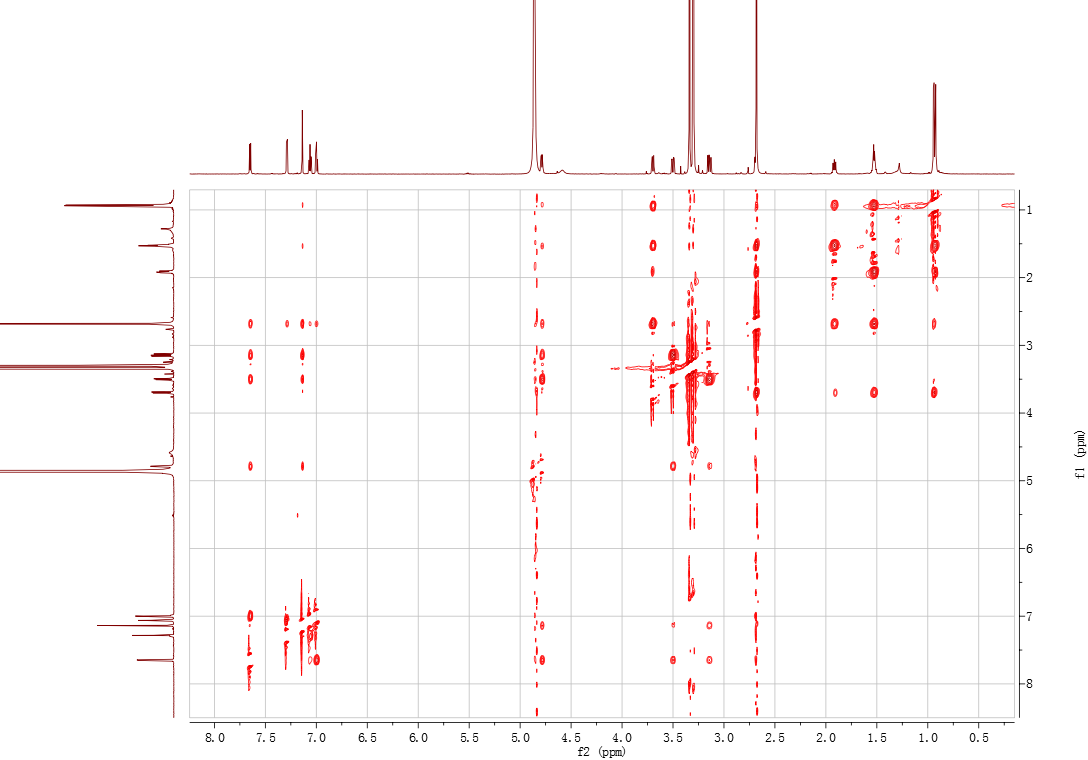


**Fig. S56.** ROESY spectrum of **7**.


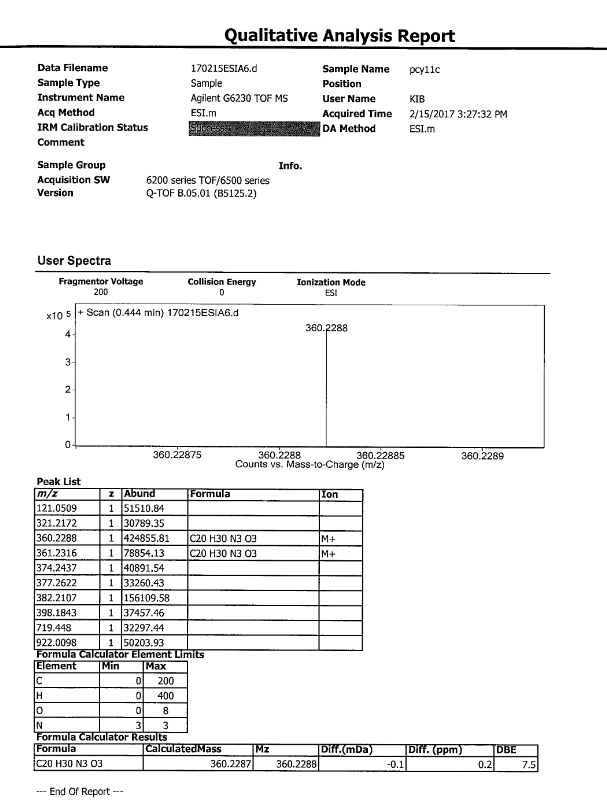


**Fig. S57.** HRESIMS spectrum of **7**.


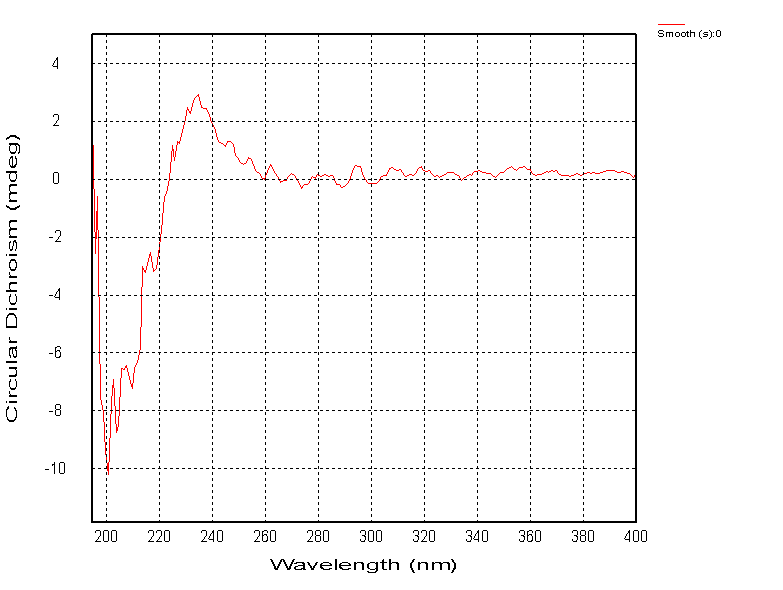


**Fig. S58.** ECD spectrum of **7**.

File: CD PCY11C-1mm(195-400)17050320.dsx

ProBinaryX

Attributes :

- Time Stamp :Wed May 03 18:53:13 2017

- File ID : {60E95004-2D3A-4015-8176-CF16DDFC8770}

- Is CFR Compliant : false

- Original unaltered data

Remarks:

- HV (CDDC channel): 0 v

- Time per point: 1 s

- Description: Sample 1

- Concentration: 0.2880mg/mL MeOH

- Pathlength: 1 mm

Settings:

- Time-per-point: 1s (25us x 40000)

- Wavelength: 195nm - 400nm

- Step Size: 1nm

- Bandwidth: 1nm


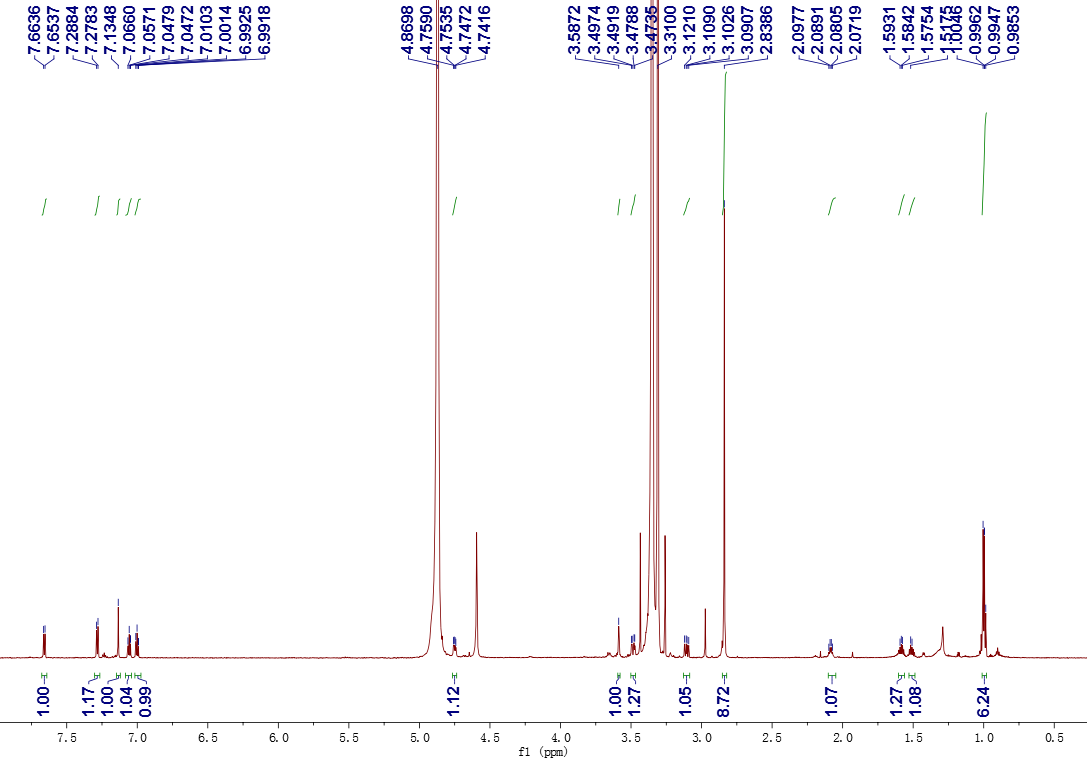


**Fig. S59.** ^1^H NMR spectrum of **8** (CD_3_OD, 800 MHz).


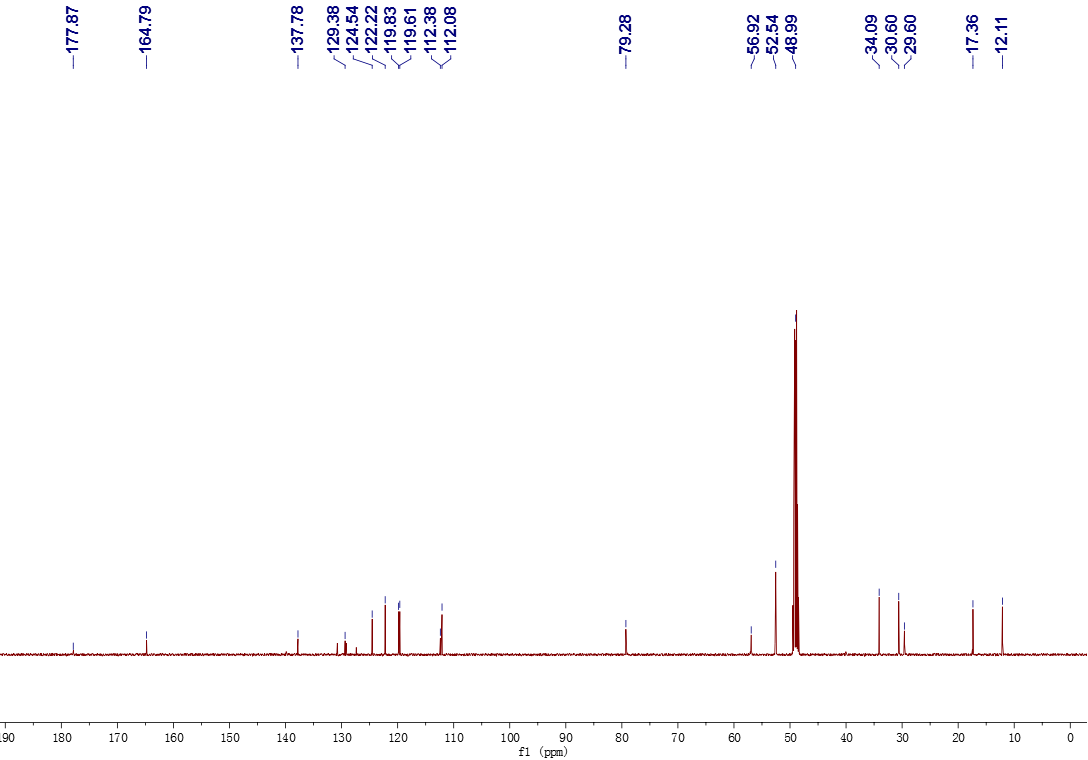


**Fig. S60.** ^1^H NMR spectrum of **8** (CD_3_OD, 125 MHz).


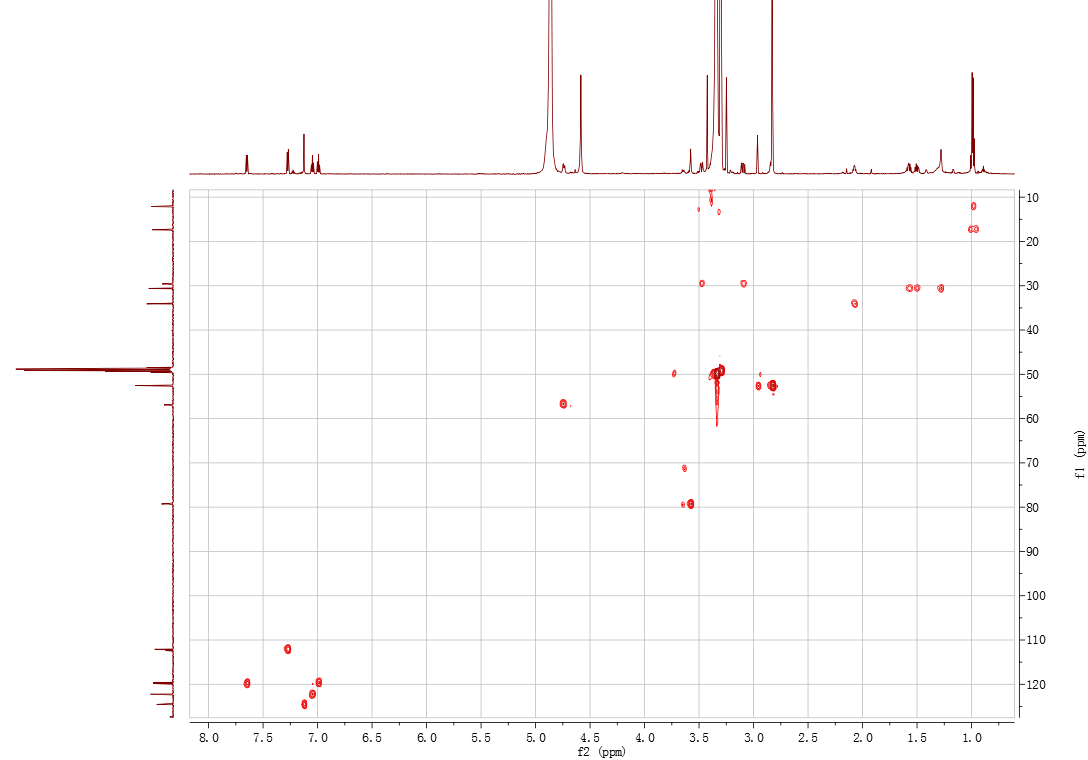


**Fig. S61.** HSQC spectrum of **8**.


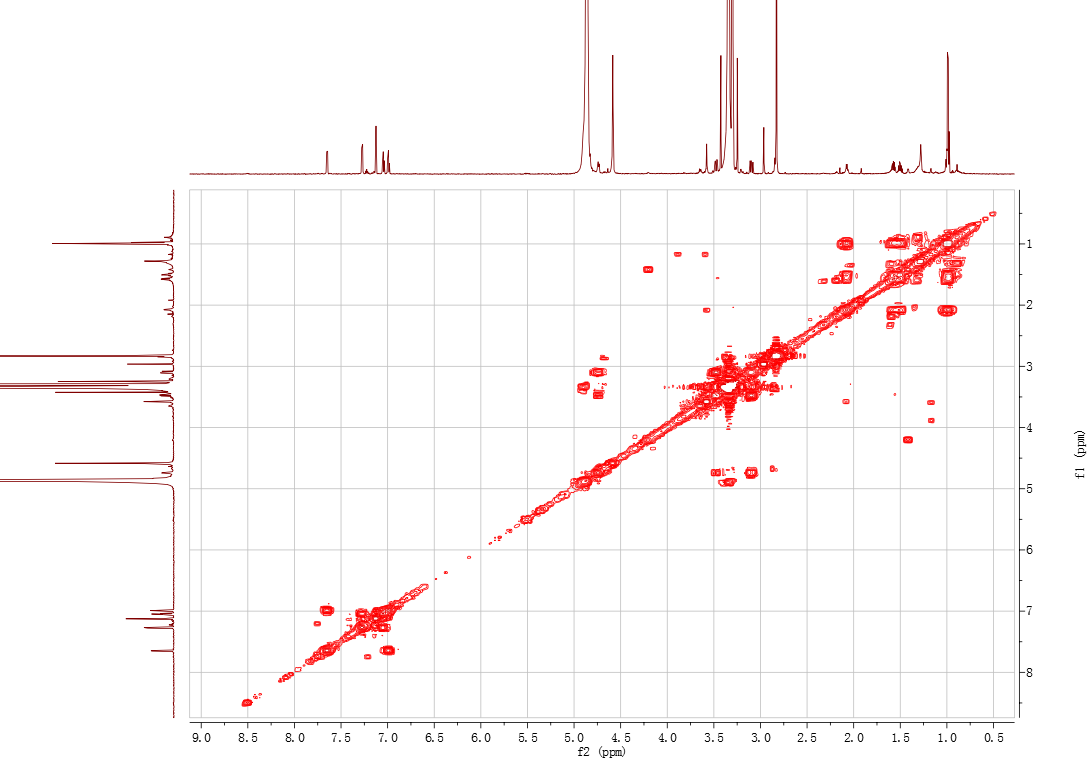


**Fig. S62.** ^1^H–^1^H COSY spectrum of **8**.


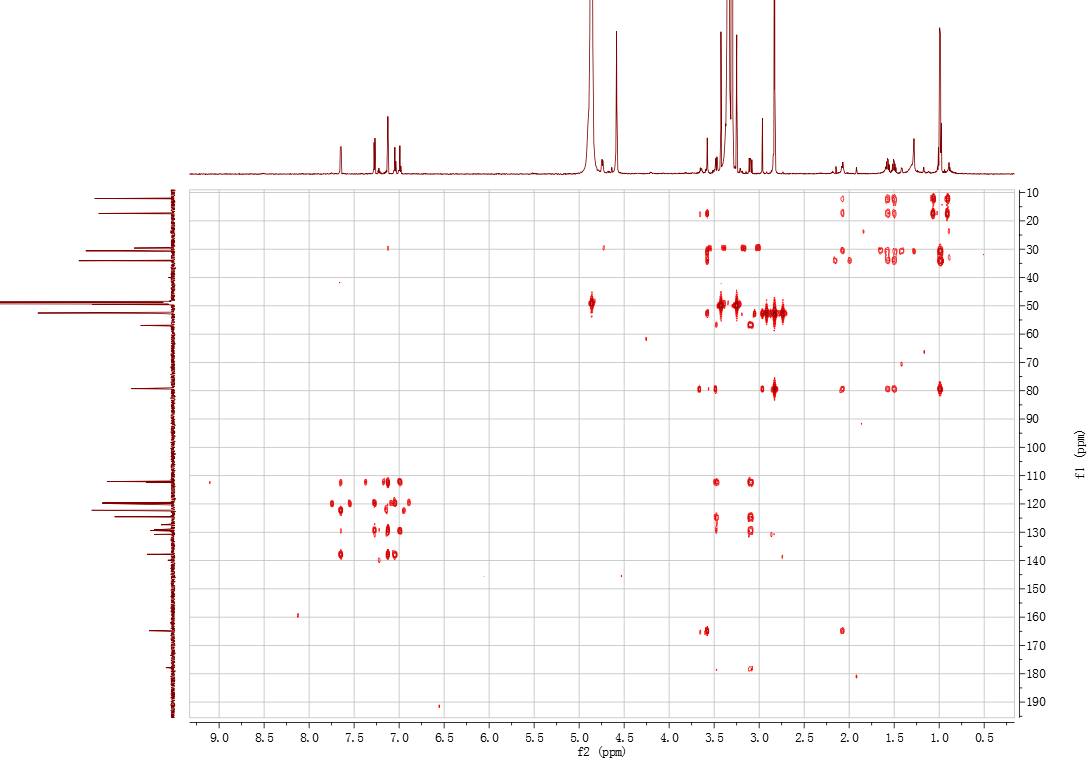


**Fig. S63.** HMBC spectrum of **8**.


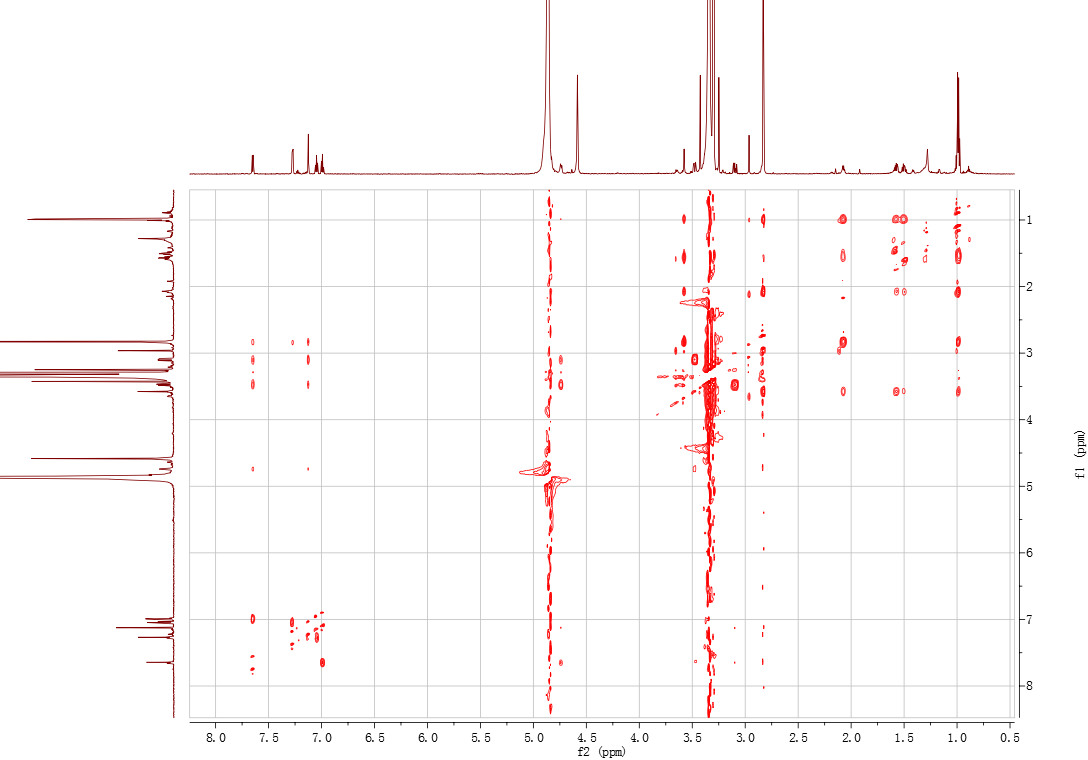


**Fig. S64.** ROESY spectrum of **8**.


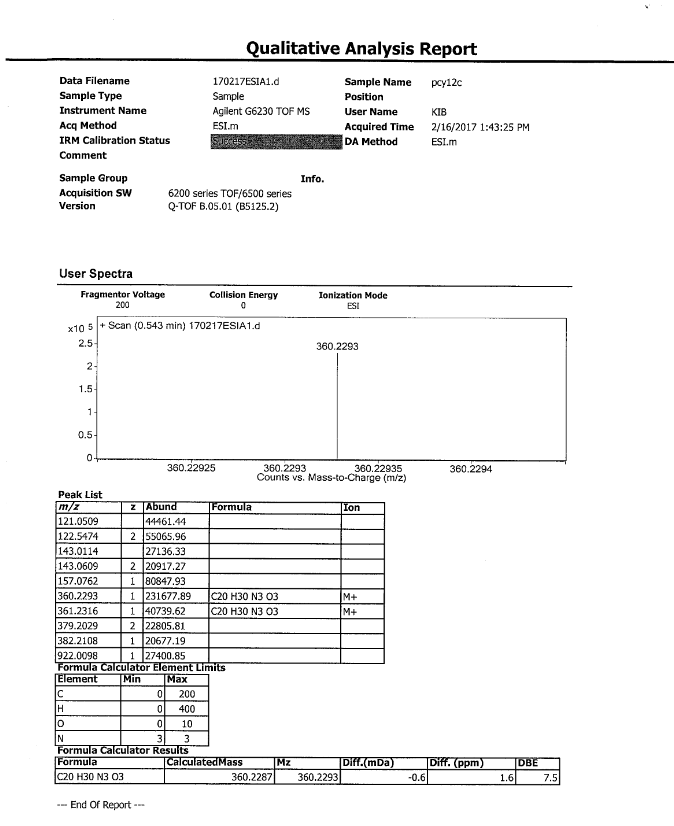


**Fig. S65.** HRESIMS spectrum of **8**.


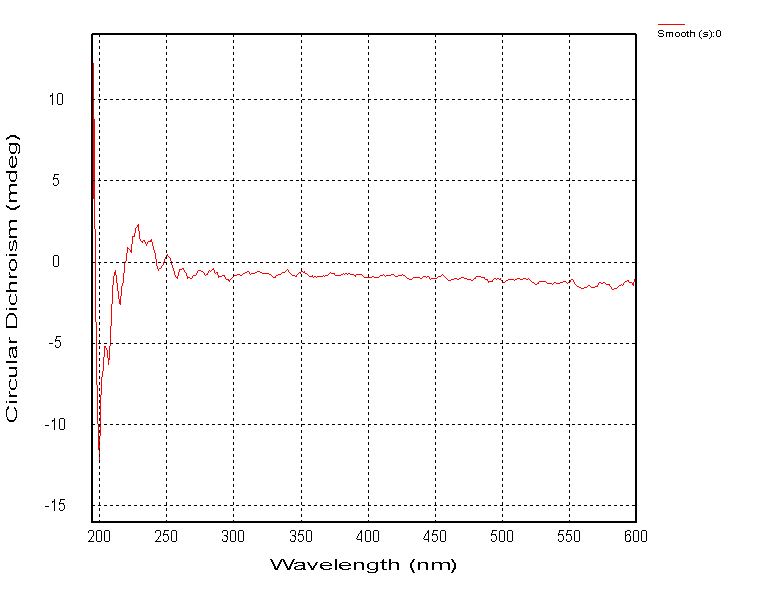


**Fig. S66.** ECD spectrum of **8**.

File: CD PCY12C-1mm(195-600)17032814.dsx

ProBinaryX

Attributes :

- Time Stamp :Tue Mar 28 14:56:14 2017

- File ID : {84E2C710-04B3-41d3-BE79-9808241F9F02}

- Is CFR Compliant : false

- Original unaltered data

Remarks:

- HV (CDDC channel): 0 v

- Time per point: 1 s

- Description: Sample 1

- Concentration: 0.2580 mg/mL MeOH

- Pathlength: 1 mm

Settings:

- Time-per-point: 1s (25us x 40000)

- Wavelength: 195nm - 600nm

- Step Size: 1nm

- Bandwidth: 1nm


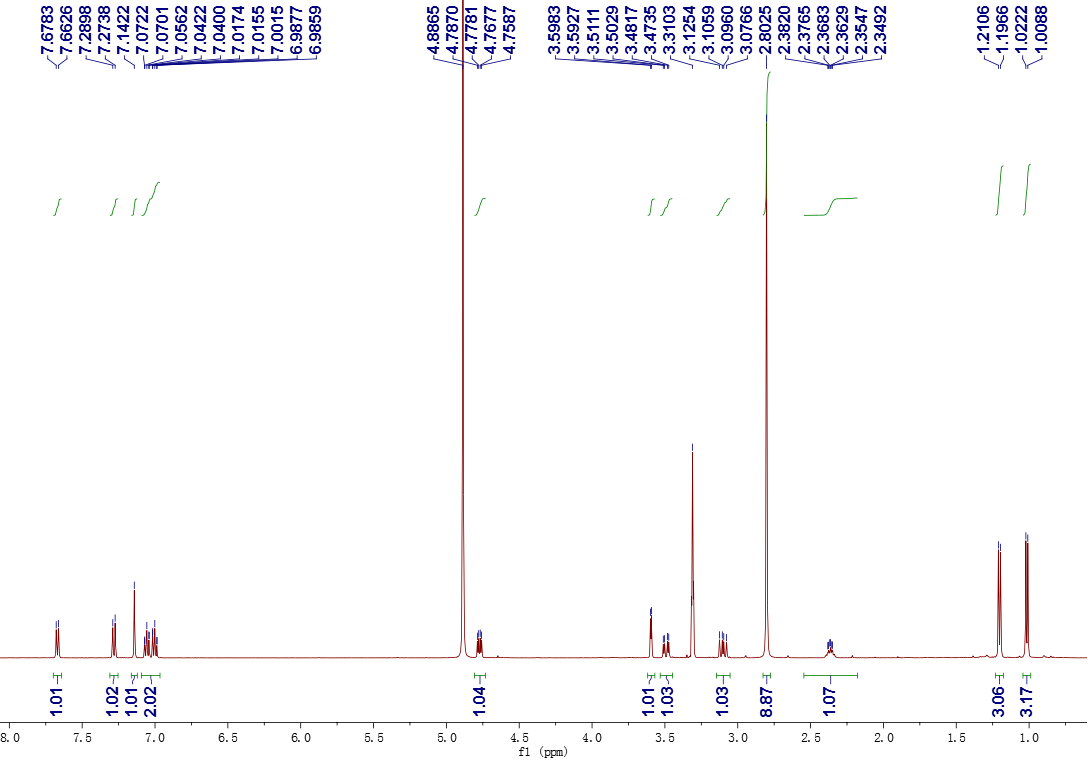


**Fig. S67.** ^1^H NMR spectrum of **9** (CD_3_OD, 500 MHz).


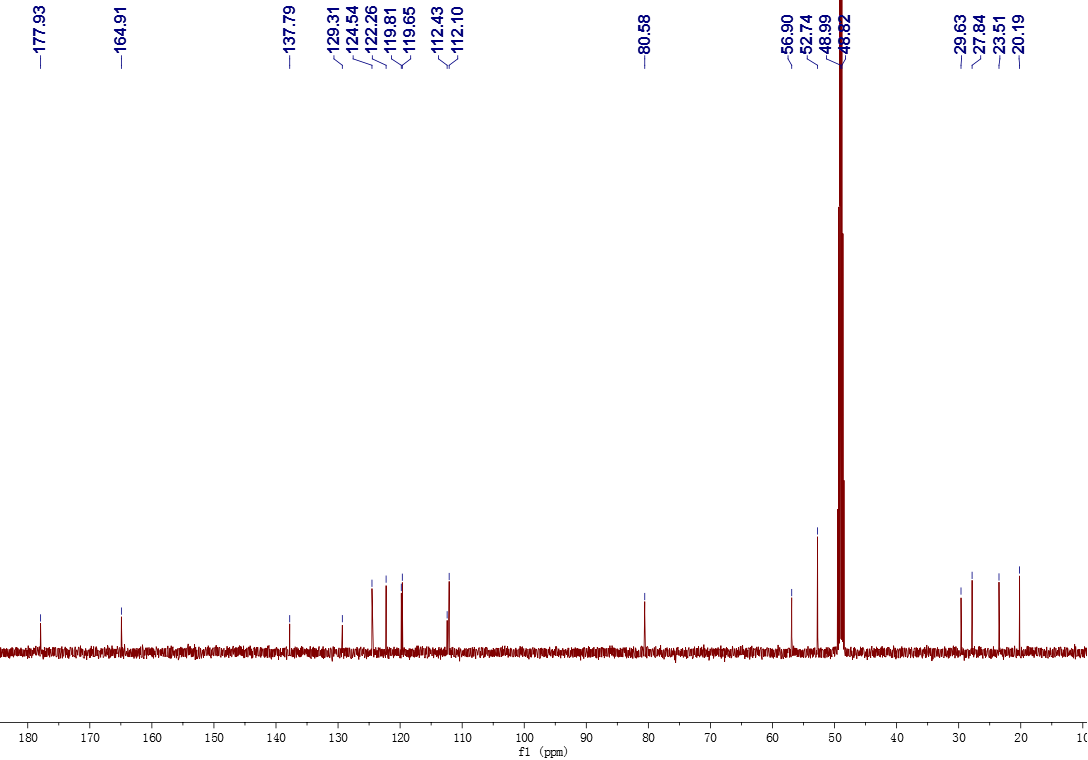


**Fig. S68.** ^13^C NMR spectrum of **9** (CD_3_OD, 125 MHz).


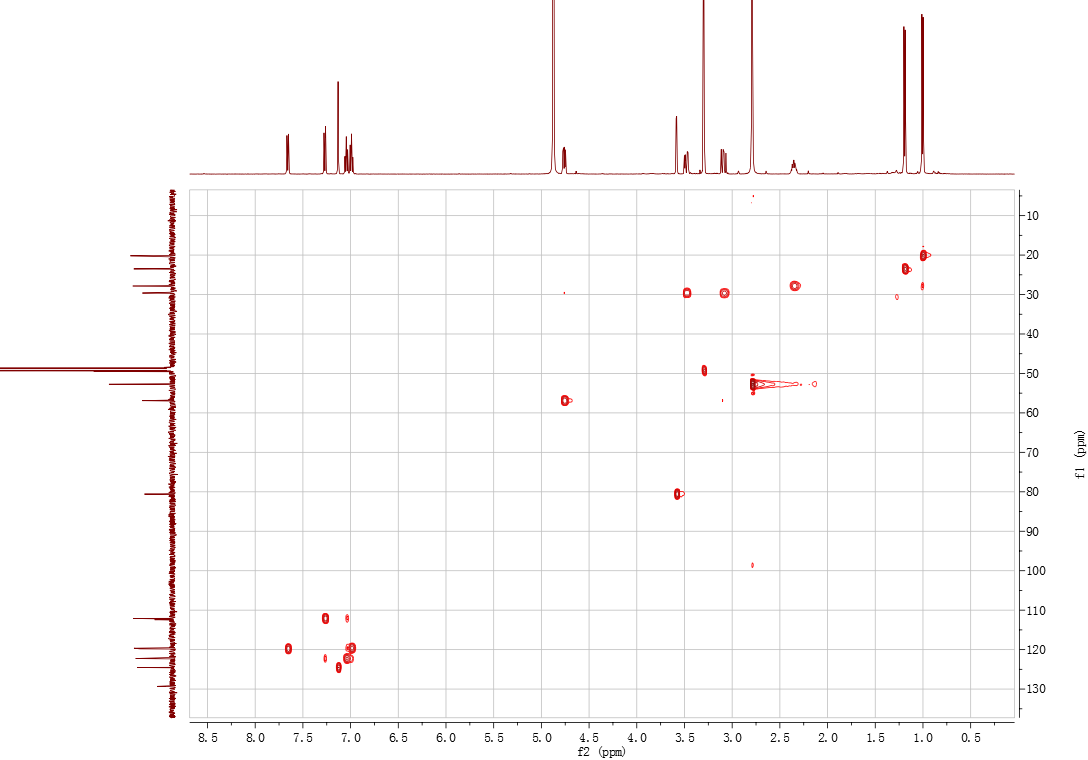


**Fig. S69.** HSQC spectrum of **9**.


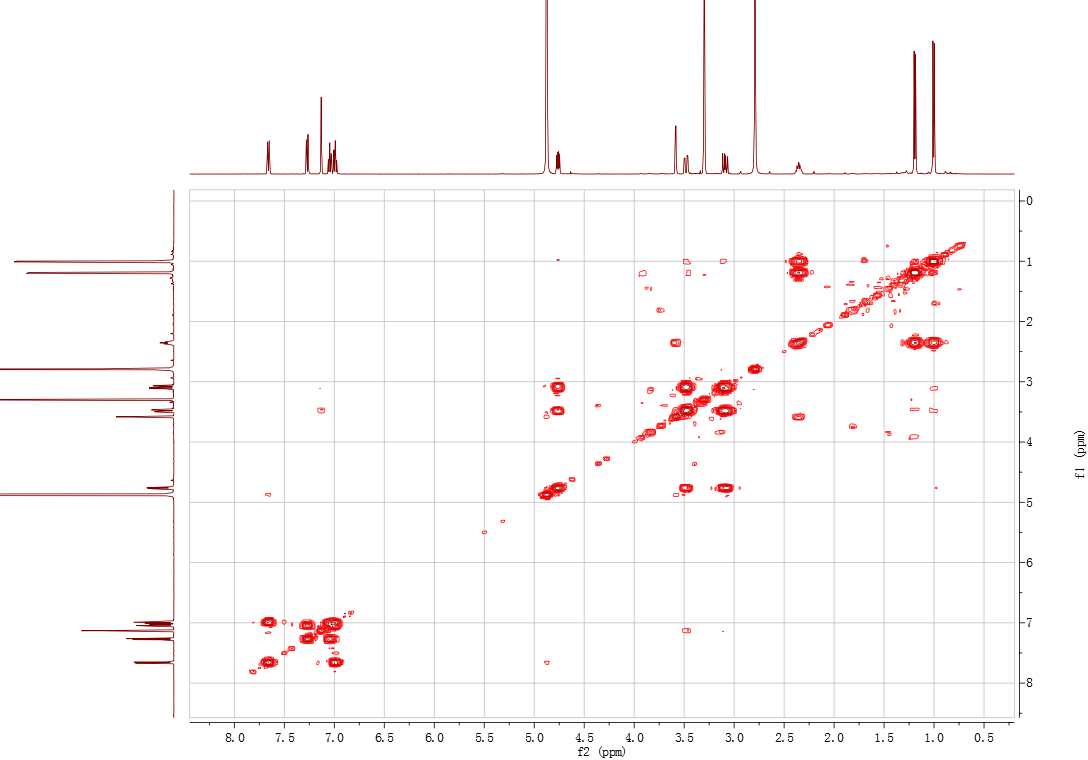


**Fig. S70.** ^1^H–^1^H COSY spectrum of **9**.


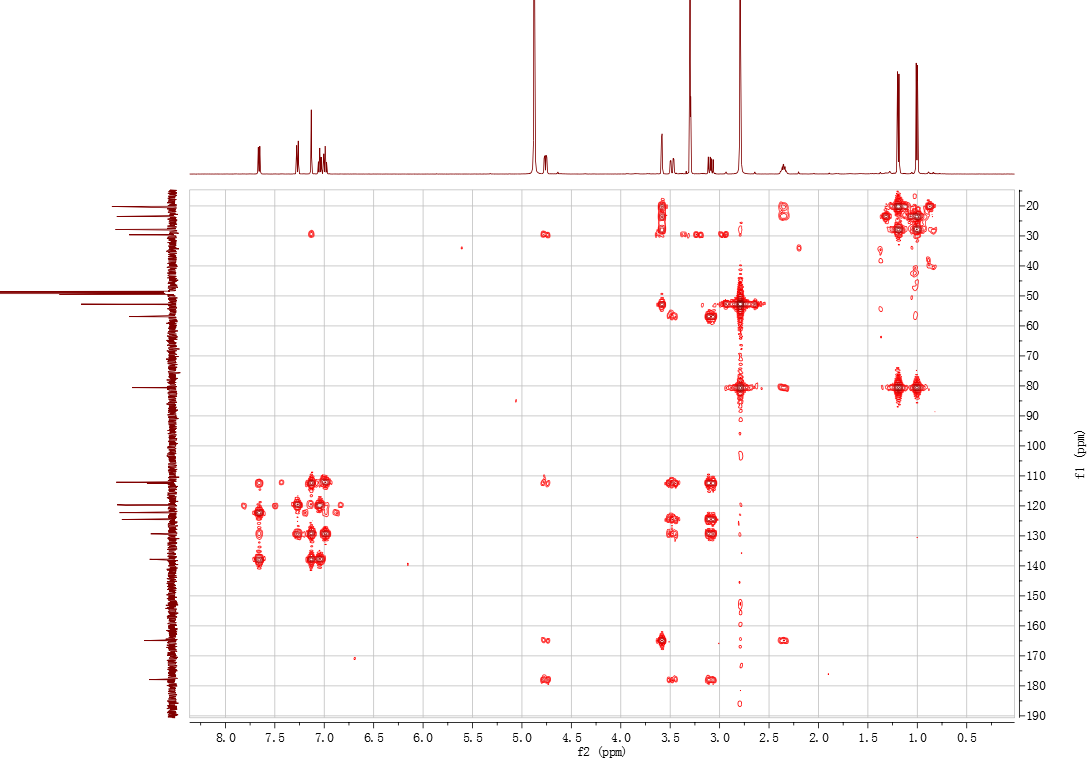


**Fig. S71.** HMBC spectrum of **9**.


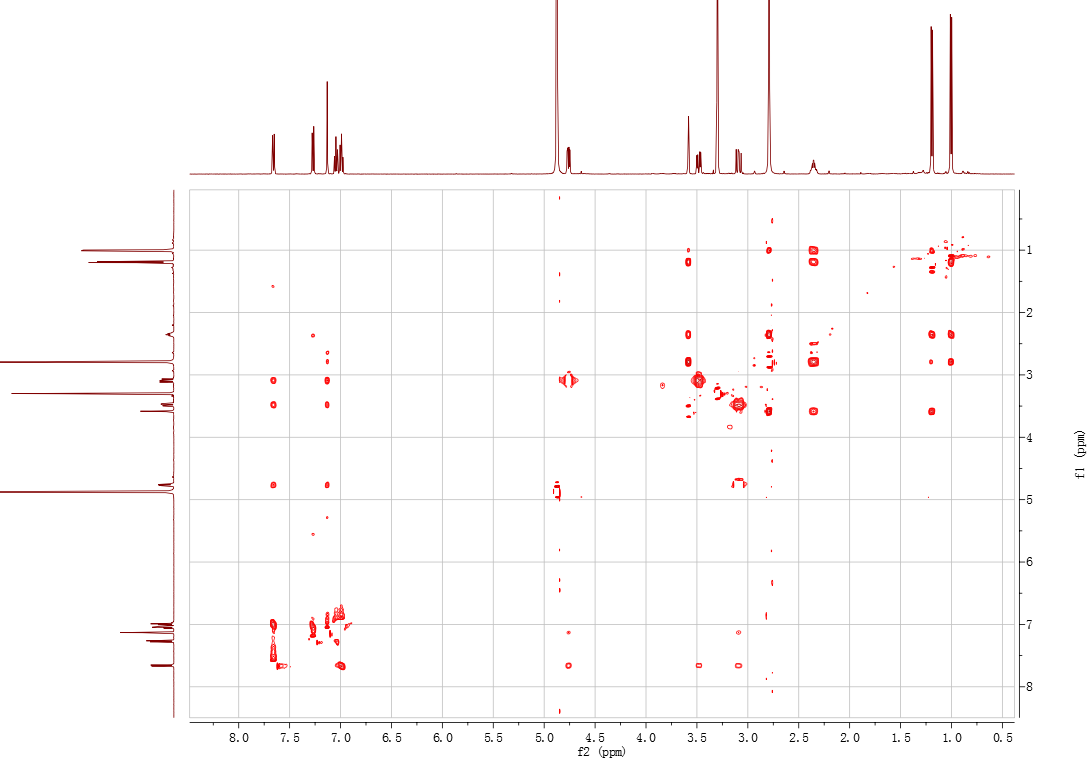


**Fig. S72.** ROESY spectrum of **9**.

**Fig. S73.** HRESIMS spectrum of **9**.


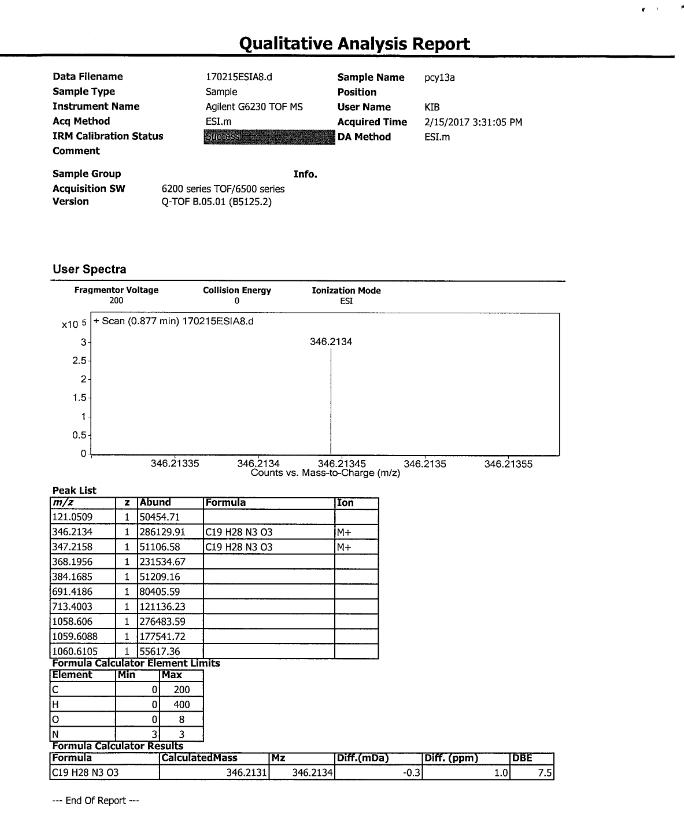


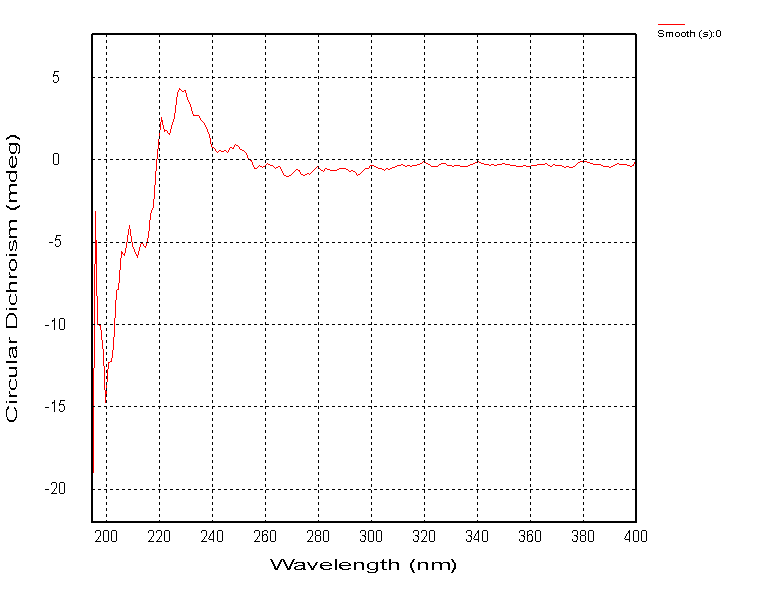


File: CD PCY13A-1mm(195-400)17012504.dsx

ProBinaryX

Attributes :

- Time Stamp :Wed Jan 25 10:16:31 2017

- File ID : {395B526F-EBD4-4394-8169-23A5BC1D78AF}

- Is CFR Compliant : false

- Original unaltered data

Remarks:

- HV (CDDC channel): 0 v

- Time per point: 1 s

- Description: Sample 1

- Concentration: 0.1344 mg/mL MeOH

- Pathlength: 1 mm

Settings:

- Time-per-point: 1s (25us x 40000)

- Wavelength: 195nm - 400nm

- Step Size: 1nm

- Bandwidth: 1nm

**Fig. S74.** ECD spectrum of **9**.
